# Synthesis, reactions, antitumor and antimicrobial activity of new 5,6-dihydropyrrolo[2,1-*a*]isoquinoline chalcones

**DOI:** 10.1186/s13065-025-01557-4

**Published:** 2025-07-08

**Authors:** Mohamed A. Mohamed Teleb, Monica G. Kamel, Madonna S. Mikhail, Hamdi M. Hassaneen, Ayman W. Erian, Mirna T. Helmy

**Affiliations:** Department of Chemistry, Faculty of Science, Cairo University, Giza, 12613 Egypt

**Keywords:** Chalcones, Dihydroisoquinoline, Hydrazonoyl halides, Claisen–Schmidt condensation, Antitumor and antimicrobial activity

## Abstract

Stirring of 2-(6,7-dimethoxy-3,4-dihydroisoquinolin-1-yl)acetonitrile **1** with thiophene-2-carbaldehyde **2** in absolute ethanol in the presence of hydrochloric acid yielded 2-(6,7-dimethoxy-3,4-dihydroisoquinolin-1-yl)-3-(thiophen-2-yl)acrylonitrile hydrochloride **3**. Refluxing of arylidene **3** with α-ketohydrazonoyl halides **4–7** in the presence of triethylamine in chloroform afforded dihydropyrrolo[2,1-*a*]isoquinolines **11–14**. Claisen–Schmidt condensation of **11** with aryl aldehydes **15a-f** or pyrazole aldehydes **17a-d** in ethanol in the presence of sodium hydroxide solution produced chalcones **16a-f** and **18a-d**. Refluxing of chalcone **16f** with hydrazine hydrate in ethanol afforded pyrazoline **19** which gave *N*-phenylcarbothioamide derivative **20** on stirring with phenyl isothiocyanate in dry ether. Also, refluxing of **19** with acetic anhydride or formic acid afforded acetyl-pyrazoline derivative **21** or formyl-pyrazoline derivative **22**, respectively. Antitumor activity for some new synthesized compounds showed that compounds **16b** and **16d** had anticancer activities. Antimicrobial activities for the newly synthesized compounds revealed the most potent compounds **16c**, **18b** and **18d** against *E. coli*, compounds **16b**, **18b** and **18d** against *B. mycoides*, and compounds **16b**, **16c** and **18b** against *C. albicans*. Moreover, compound **18b** had the lowest MIC values against *E. coli* and *B. mycoides*, with MIC values of 40 and 60 µg/ml, respectively.

**Supplementary Information:**

The online version contains supplementary material available at 10.1186/s13065-025-01557-4.

## Introduction

**Fig. 1:**
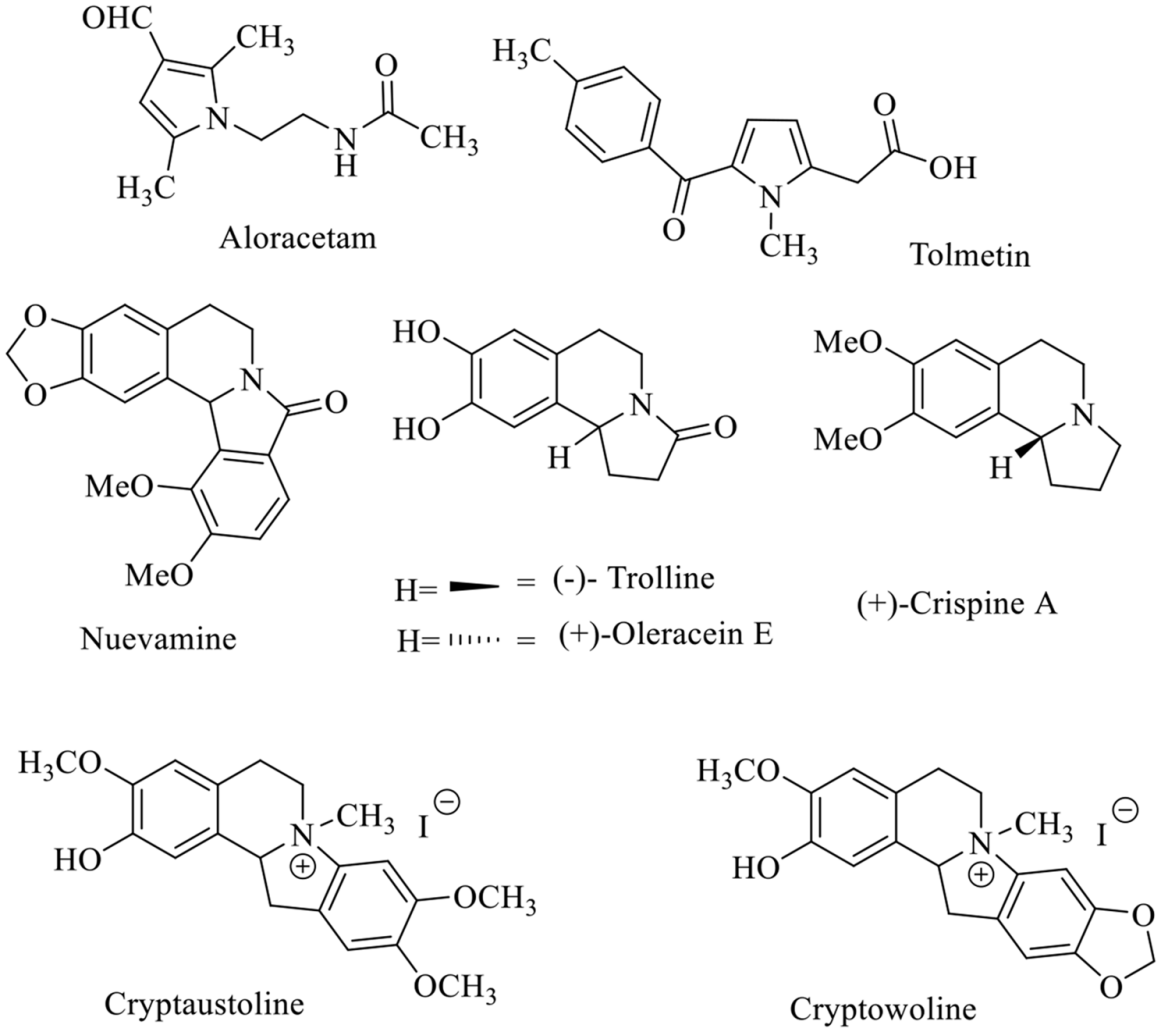
Pharmacologically active compounds

The term of chalcone was created by the two authors, Stanislaw Kostanecki and Joseph Tambor [1]. Chalcones are exist in some naturally occurring substances from the flavonoids family. There are several conjugated compositions for chalcones, that known as 1,3-diphenylprop-2-en-1-one, in which the keto-ethylenic system (an *α*,*β*-unsaturated carbonyl ketone, -CO-CH═CH-) linked the two aromatic rings [2, 3]. They can exist with either the more stable trans-form or the less stable cis-form. Moreover, chalcones are the major ingredient of a wide variety of natural goods, including fruits, vegetables, teas, and other plants that are highly valued for their biological activities [4, 5, 6, 7, 8, 9, 10, 11, 12, 13, 14, 15, 16, 17]. The most common methods for synthesizing chalcones *via* Suzuki reaction among phenyl boronic acid and cinnamoyl chloride or between phenyl vinyl boronic acid and benzoyl chloride [18], Heck reaction by coupling an aryl vinyl ketone with an aryl halide [19], Sonogashira coupling of the electron-insufficient group [20, 21], Miscellaneous reaction by coupling of phenylacetylene with benzaldehyde [22], Aldol reaction of ketones with benzaldehyde [23, 24]. Also, Claisen-Schmidt condensation for example *via* reaction between isoquinolines containing acetyl group and substituted aryl aldehydes [8, 25, 26, 27]. Chalcones exhibited a broad spectrum of biological activities such as antioxidant [4, 12, 28, 29, 30], analgesic [17], antiplatelet [16], anti-pyretic [31], antimalarial [15, 32], anti-inflammatory [11, 12, 13, 33], anticancer agents [4, 5, 6, 7, 8, 9, 10, 11, 12, 13, 14, 15, 16, 17, 34, 35, 36], antifungal [10, 37] and antibacterial [5, 9, 38]. Also, heterocycles such as thiophenes [39], pyrazoles [40–41] and isoquinolines [42], are widely exist in pharmaceuticals and play an important role in medicinal chemistry. Besides, pyrrole exist in a number of pharmaceutical products and new active agents with a variety of pharmacological effects like: Aloracetam for treatment of Alzheimer disease, and Tolmetin anti-inflammatory (Fig. 1) [43, 44]. Moreover, fused heterocycles such as pyrroloisoquinoline and isoindoloisoquinolinone are present in pharmacologically active alkaloids like Nuevamine, Trolline, Oleracein E as well as Crispine A [45]. The indolo[2,1-*a*]isoquinolines are the principal structural moieties of Cryptaustoline and Cryptowoline alkaloids which exhibit strong anticancer activity and affinity for estrogen receptors. (Fig. 1) [46]. In continuation of our related work [8, 47, 48, 49, 50, 51], our goal of the present work is the synthesis of novel chalcones bearing pyrrolo[2,1-*a*]isoquinoline moiety and investigating their biological activity in vitro as anticancer agents against two human cancer cell lines: MCF7 (human Caucasian breast adenocarcinoma) and A549 (lung carcinoma), and antimicrobial activities for the newly synthesized compounds against *E. coli*, *B. mycoides*, and *C. albicans*.

## Results and discussion

**Scheme 1:**
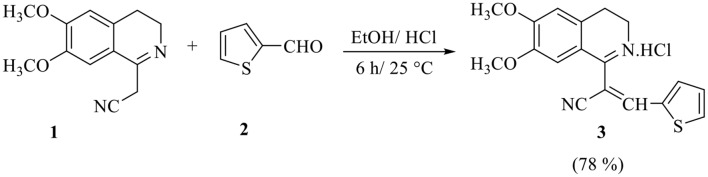
Synthesis of arylidene **3**

Stirring of a mixture of 2-(6,7-dimethoxy-3,4-dihydroisoquinolin-1-yl)acetonitrile **1** [52, 53] with thiophene-2-carbaldehyde **2** in absolute ethanol in the presence of concentrated hydrochloric acid at room temperature yielded 2-(6,7-dimethoxy-3,4-dihydroisoquinolin-1-yl)-3-(thiophen-2-yl)acrylonitrile hydrochloride **3***via* Knoevenagel condensation (Scheme 1).

**Scheme 2:**
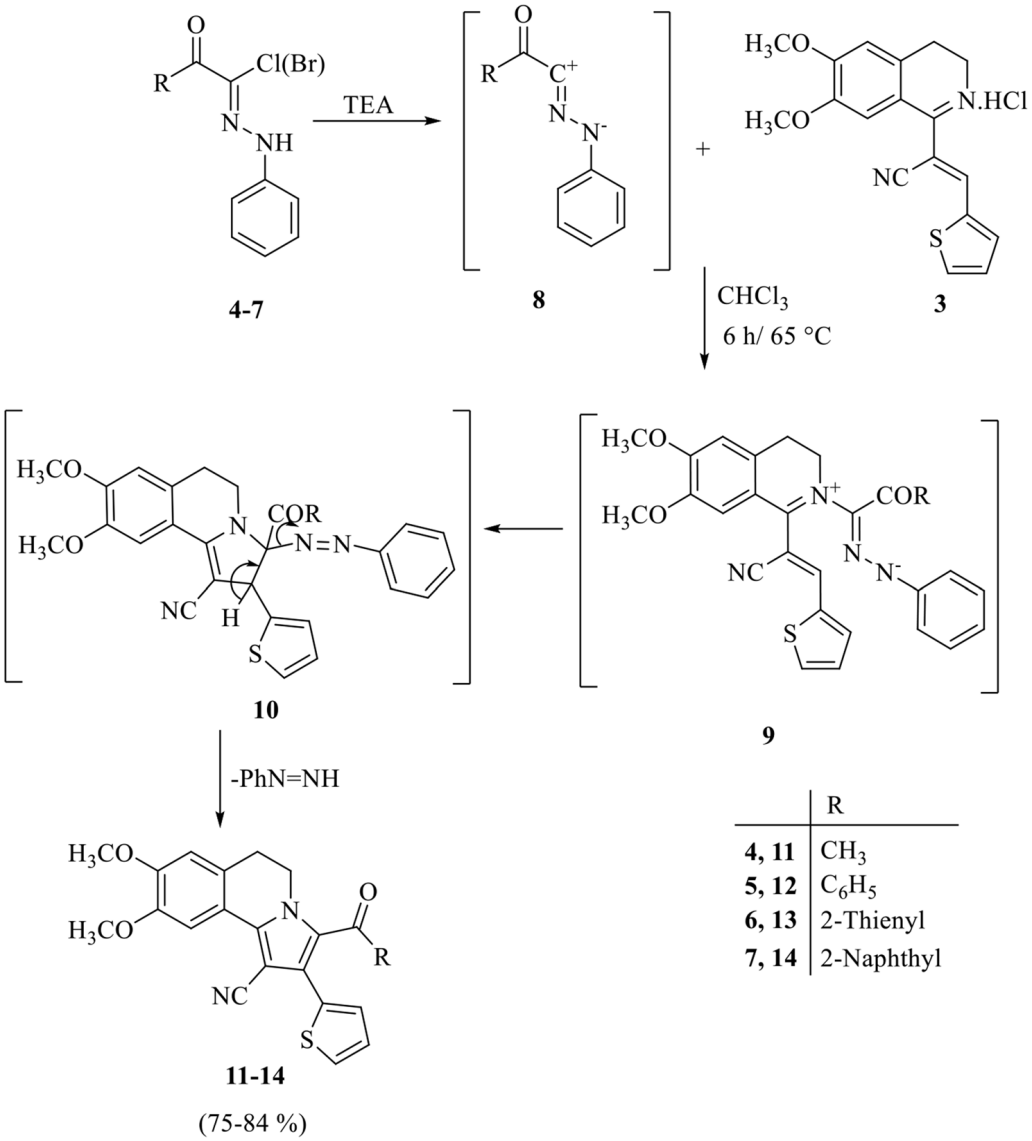
Synthesis of 3-acetyl or 3-aroyl-8,9-dimethoxy-2-(thiophen-2-yl)-5,6-dihydropyrrolo[2,1-*a*]isoquinoline-1-carbonitriles **11–14**

Reaction of arylidene **3** with *α*-ketohydrazonoyl halides **4–7** [54, 55, 56, 57] in refluxing chloroform using triethylamine as a catalyst afforded 3-acetyl or 3-aroyl-8,9-dimethoxy-2-(thiophen-2-yl)-5,6-dihydropyrrolo[2,1-*a*]isoquinoline-1-carbonitriles **11–14** (Scheme 2).

According to the suggested mechanism, nitrilimines **8**, produced in situ by reaction of *α*-ketohydrazonoyl halides **4–7** with triethylamine in chloroform, reacted with arylidene **3** to give the intermediate **9** which underwent in situ 1,5-electrocyclization then lost phenyldiazene molecule to yield products **11–14**. The skeletons of the products **11–14** were confirmed by their elemental analyses and spectral data (^1^H NMR and ^13^C NMR). The ^1^H NMR spectrum of compound **11**, as example, revealed five singlet signals at *δ* 2.12, 3.94, 3.95, 6.78 and 7.80 corresponding to acetyl group, two OCH_3_ groups, two protons at C_7_ and C_10_ of isoquinoline, respectively. In additions, three multiplet signals at *δ* 3.01–3.05, 4.59–4.63 and 7.14–7.50 corresponding to two CH_2_ groups at C_5_ and C_6_ of isoquinoline and three thienyl protons, respectively. Also, 21 signals for the asymmetric carbons appeared in its ^13^C NMR spectrum.

**Scheme 3:**
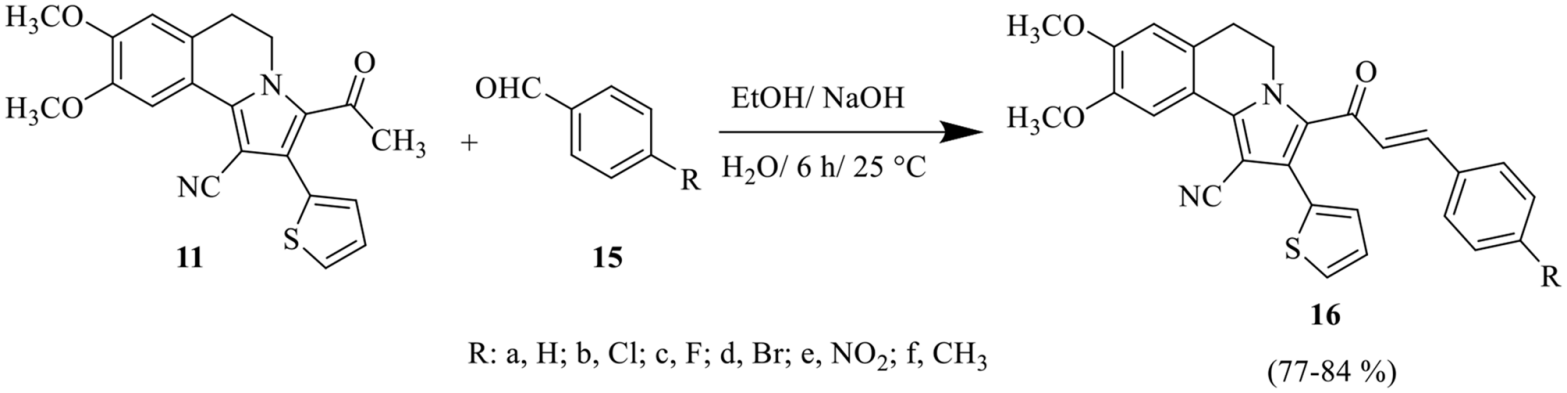
Synthesis of pyrrolyl chalcones **16a-f**

**Scheme 4:**
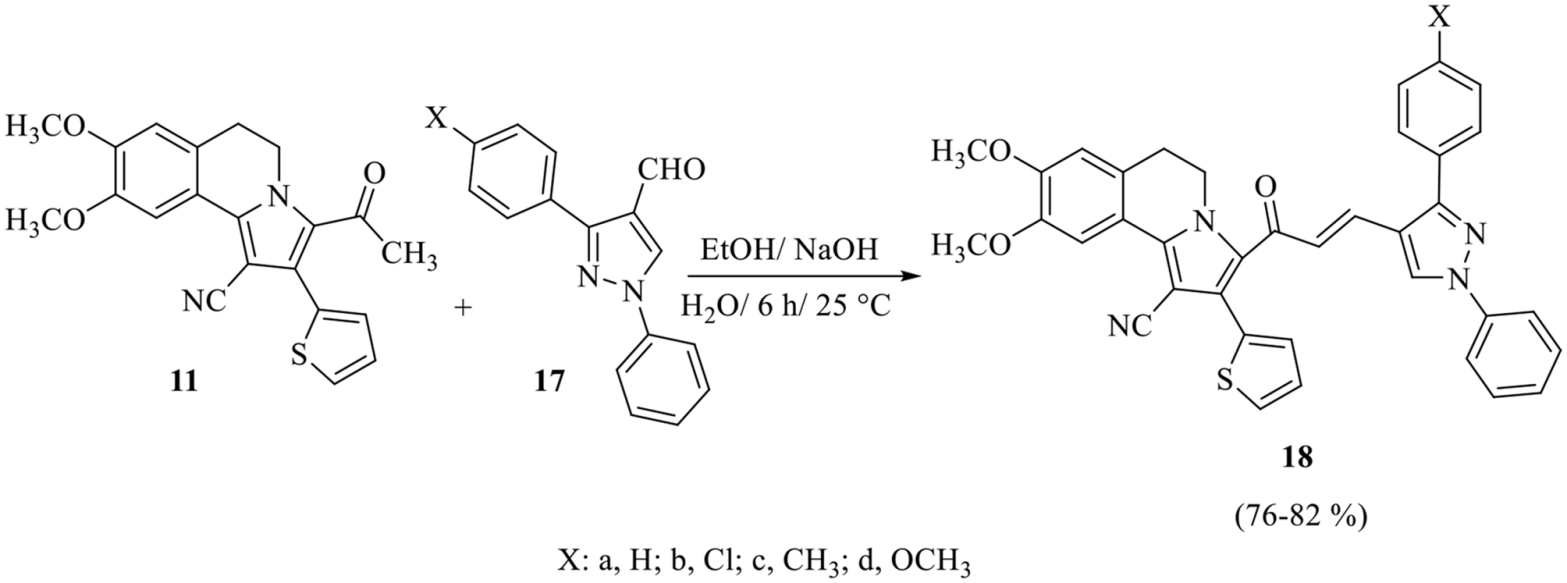
Synthesis of pyrazolyl chalcones **18a-d**

Claisen–Schmidt condensation of 3-acetyl-8,9-dimethoxy-2-(thiophen-2-yl)-5,6-dihydropyrrolo[2,1-*a*]isoquinoline-1-carbonitrile **11** with equimolar amounts of aryl aldehydes **15a-f** or pyrazolyl aldehydes **17a-d** [58] in ethanol in the presence of sodium hydroxide solution yielded the corresponding chalcones **16a-f** and **18a-d**, respectively (Scheme 3 and 4). The constitutions of these chalcones were confirmed from their spectral data and elemental analyses. The ^1^H NMR spectrum of chalcone **16f**, as example, revealed five singlet signals at δ 2.35, 3.96, 3.98, 6.80 and 7.86 corresponding to protons of CH_3_ group, protons of two OCH_3_ groups and two protons at C_7_ and C_10_ of isoquinoline, respectively, three multiplet signals at *δ* 3.06–3.10, 4.63–4.67, 7.11–7.51 corresponding to protons of two CH_2_ group at C_5_ and C_6_ of isoquinoline and seven aromatic protons, respectively. Also, it showed two doublet signals corresponding to the two vinyl protons at δ 6.66 and 7.57 with coupling constant *J* = 15.6 Hz which confirms the trans configuration of the two vinyl protons. The structure was also confirmed by ^13^C NMR which revealed 27 signals for distinct carbon atoms.

**Scheme 5:**
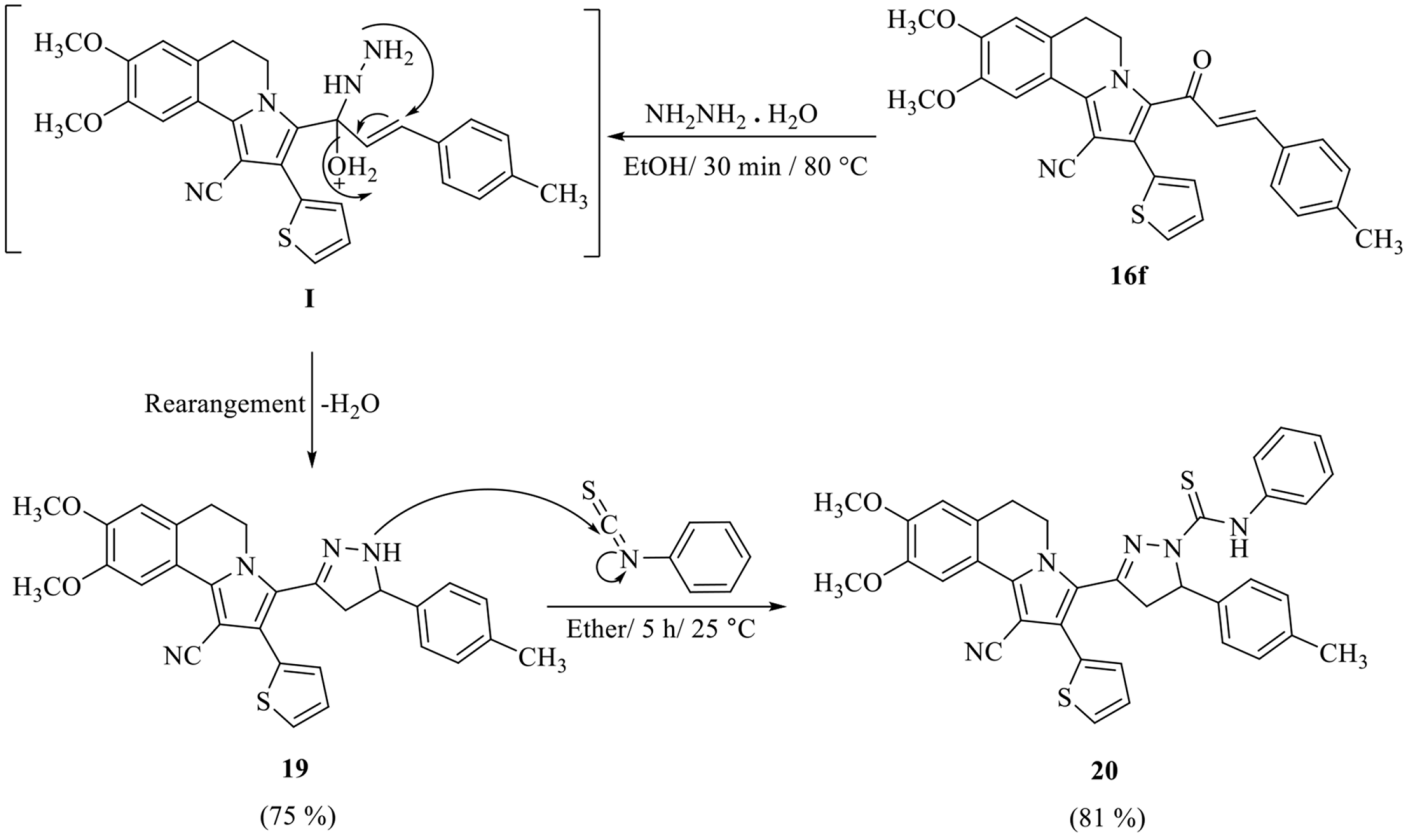
Synthesis of pyrazoline derivatives **19** and **20**

The chalcone **16f** was reacted with hydrazine hydrate in boiling ethanol to afford 8,9-dimethoxy-2-(thiophen-2-yl)-3-(5-(*p*-tolyl)-4,5-dihydro-1*H*-pyrazol-3-yl)-5,6-dihydropyrrolo[2,-1-*a*]isoquinoline-1-carbonitrile **19** which gave 3-(1-cyano-8,9-dimethoxy-2-(thiophen-2-yl)-5,6-dihydropyrrolo[2,1-*a*]isoquinolin-3-yl)-*N*-phenyl-5-(*p*-tolyl)-4,5-dihydro-1*H*-pyrazole-1-carbothioamide **20** on stirring with phenyl isothiocyante in dry ether (Scheme 5).

**Scheme 6:**
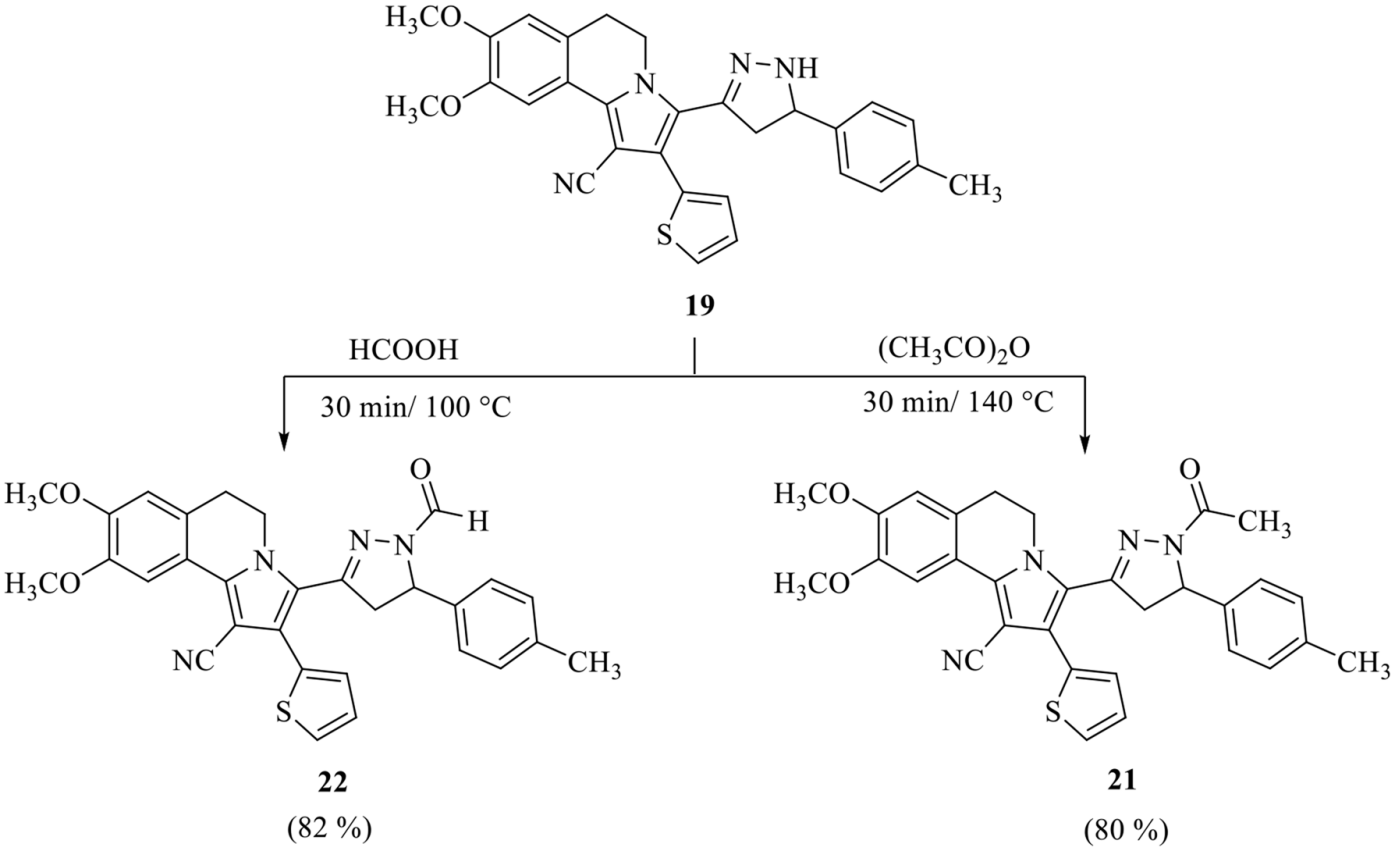
Synthesis of *N*-acetyl and *N*-formylpyrazoline derivative **21** and **22**

Moreover, 3-(1-acetyl-5-(*p*-tolyl)-4,5-dihydro-1*H*-pyrazol-3-yl)-8,9-dimethoxy-2-(thiophen-2-yl)-5,6-dihydropyrrolo[2,1-*a*]isoquinoline-1-carbonitrile **21** and 3-(1-formyl-5-(*p*-tolyl)-4,5-dihydro-1*H*-pyrazol-3-yl)-8,9-dimethoxy-2-(thiophen-2-yl)-5,6-dihydropyrrolo[2,1-*a*]isoquinoline-1-carbonitrile **22** were prepared *via* refluxing of pyrazoline derivative **19** with acetic anhydride or formic acid, respectively (Scheme 6). The structures of the isolated products were established by their spectral data and elemental analyses (see Experimental). The ^1^H NMR spectrum of compound **21**, as example, showed six singlet signals at *δ* 2.31, 2.37, 3.96, 3.97, 6.80 and 7.81 corresponding to CH_3_ group, acetyl group, two OCH_3_ groups, two protons at C_7_ and C_10_ of isoquinoline, respectively, six multiplet signals at *δ* 2.64–2.72, 3.09–3.13, 3.26–3.35, 4.61–4.67, 5.32–5.35 and 7.01–7.38 corresponding to three protons of pyrazoline, two CH_2_ group at C_5_ and C_6_ of isoquinoline and seven aromatic protons. Also, 29 signals appeared in the ^13^C NMR spectrum for the distinct carbons.

### Antitumor activity

MCF7 (breast cancer cell line isolated in 1970 from a 69-year-old Caucasian woman) and A549 (lung carcinoma) were obtained from the American type culture collection (ATCC). The cells were propagated in DMEM supplemented with 10% heat-inactivated FBS, 1% L-glutamine, HEPES buffer and 50 µg/mL gentamycin. All cells were maintained at 37 °C in humidified atmosphere with 5% CO_2_ and were subcultured two times a week. Cell toxicity was monitored by determining the effect of the test samples on cell morphology and cell viability. Cells were suspended in RPMI 1640 medium for MCF7 and in DMEM for A549, 1% antibiotic-antimycotic mixture (10,000 U/mL Potassium Penicillin, 10,000 µg/mL Streptomycin Sulfate and 25 µg/mL Amphotericin B) and 1% L-glutamine at 37 ºC under 5% CO_2_. The results showed that compounds **16b** and **16d** possess moderate antitumor activities in the range of 56.8–77.6% on MCF7 and A549 cell lines. Ther rest of the compounds showed low activity against MCF7 and A549 cell lines (Table 1).

**Table 1:** In vitro antitumor activity of some newly synthesized Chalcones

Sample Code	MCF7 [Human Caucasian breast adenocarcinoma]	A549 [Lung carcinoma cell line]
	IC_50_	IC_50_
**16b**	60.3	81.1
**16c**	----	----
**16d**	62.7	82.2
**18b**	----	----
**18c**	----	----
**18d**	----	----
Control (DMSO)	0	0
Negative control	0	0

Lethal concentration of the sample which causes the death of 50% of cells in 48 hrsIC90: Lethal concentration of the sample which causes the death of 90% of cells in 48 hrs

### MTT assay

Yellow MTT (3-(4,5-dimethylthiazol-2-yl)-2,5-diphenyl tetrazolium bromide) was reduced to purple formazan in a mitochondrial dependent method to determine cell viability [59]. 

All of the following processes were carried out in a sterile environment utilizing a Laminar flow cabinet with a biosafety rating of II.

Cells were batches generated for 10 days before being seeded at a concentration of 10 × 10^3^ cells/well in new complete medium for growth in 96-well microtiter plastic plates at 37 ºC for 24 h under 5% CO_2_ utilizing a water jacketed Carbon dioxide isolator. The media was extracted, a fresh medium (without serum) was inserted, and cells were cultured either alone (negative control) or with sample of various concentration to get the desired concentration of (100-50-25-12.5-6.25-3.125-0.78 and 1.56 ug/mL). After 48 h of the incubation process, aspirate the medium and add 40 ul of MTT salt (2.5 µg/mL) to each well. Incubate for another four hours at 37ºC with 5% CO_2_. To terminate the reaction and to dissolve the generated crystals, 200 µL of 10% SDS (Sodium dodecyl sulphate) in the presence of deionized water has been added for each well and left overnight at 37ºC. A positive control of 100 µg/mL was utilized as a recognized cytotoxic natural substance, resulting in 100% fatality within the identical conditions [60, 61]. 

**Fig. 2:**
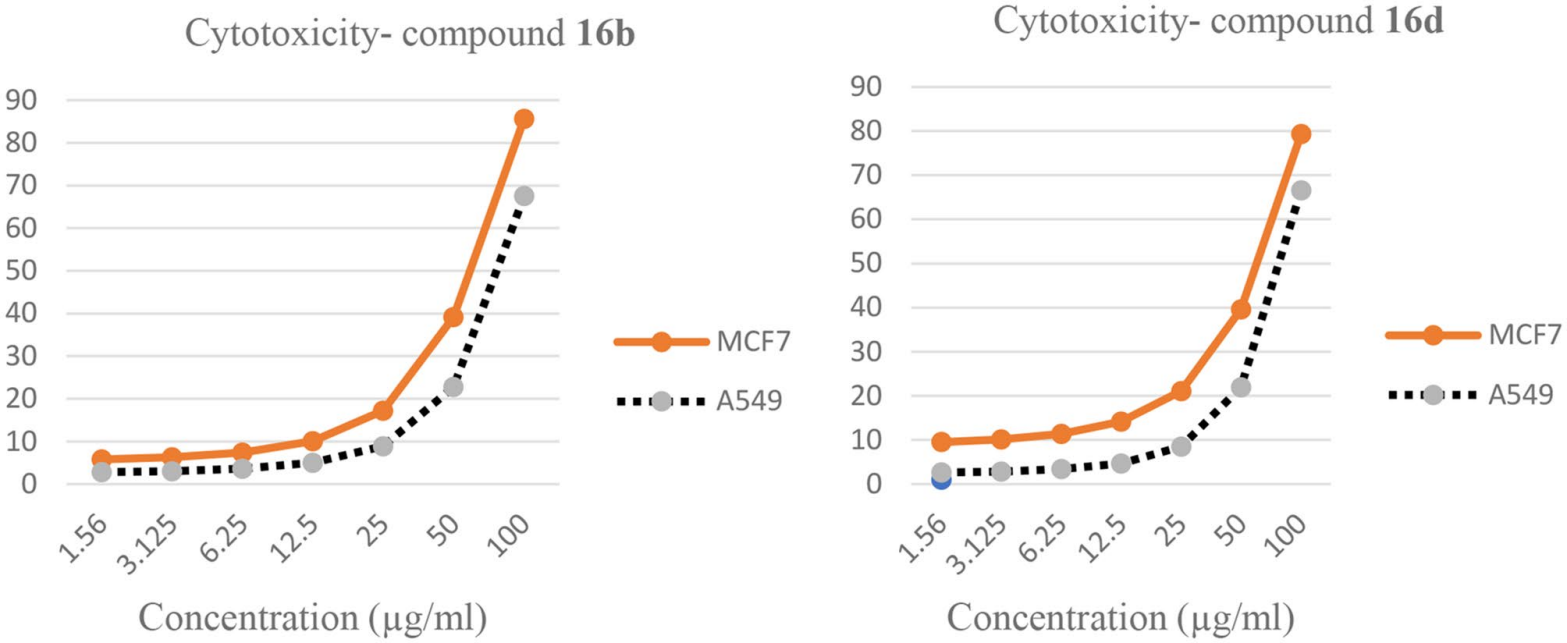
In vitro cytotoxicity of tested compounds **16b** and **16d** at different concentration using MTT test

The absorption was also determined with a microplate multi-well detector with 595 nm and a baseline wavelength at 620 nm. Statistical significance was determined among samples and negative controls (cells with vehicle) using the SPSS 11 program’s independent t-test. The percentage for change in viability was estimated using the following formula: ((Reading of extract/Reading of negative control) -1) x 100 (Fig. 2).

### Antimicrobial activity

This study used three microbiological species: Escherichia coli for Gram-negative bacteria, Bacillus mycoides for Gram-positive bacteria, and Candida albicans for yeast. The examined microbial species evolved on nutrient agar (70148 Nutrient agar, Fluka, Spain) overnight at 37 °C and used for inoculation (Table 2).

**Table 2:** Antimicrobial activity assessment of the new synthesized compounds using agar diffusion method

Compound	Inhibition zones diameter (mm)		
	G^−^	G^+^	Fungi
	E. coli	B. mycoides	C. albicans
16a	10 ± 0.00	12 ± 0.00	11 ± 0.04
16b	11 ± 0.96	17 ± 0.33	16 ± 0.44
16c	18 ± 0.85	11 ± 0.48	16 ± 1.32
16d	11 ± 0.18	11 ± 0.83	10 ± 0.07
16e	10 ± 1.07	10 ± 0.22	11 ± 0.46
16f	12 ± 0.02	12 ± 0.14	10 ± 1.00
18a	11 ± 1.06	10 ± 0.00	11 ± 0.00
18b	18 ± 1.08	17 ± 0.18	16 ± 0.46
18c	11 ± 0.68	11 ± 0.65	12 ± 0.23
18d	18 ± 1.11	17 ± 1.00	10 ± 0.44
19	10 ± 0.05	12 ± 0.57	11 ± 0.08
20	12 ± 0.26	10 ± 0.00	10 ± 0.00
21	11 ± 0.15	11 ± 0.07	12 ± 0.23
22	10 ± 0.35	11 ± 0.71	11 ± 0.00
Gentamicin (Antibacterial agent) Amphotericin B (Antifungal agent)	11 ± 0.05	12 ± 0.33	15 ± 0.25

In the inoculated agar plates, 100 µL of dissolved chemicals (10 mg/mL) in DMSO were administered to 12 mm holes, and incubated overnight at 37 °C

To investigate the efficacy of the produced compounds as antimicrobial agents, 100 µL of resuspended overnight cultures at 37 °C (1 × 10^7^ CFU/100 µL) were administered into the nutrient agar medium (70148 Nutrient agar, Fluka). The well diffusion experiment involved distributing the relevant compounds in wells (12 mm in diameter) perforated with a sterile cork borer in nutrient agar medium (70148 Nutrient agar, Fluka, Spain). 100 µL of each antiseptic solution (10 mg/mL) was inserted into each well. Gentamicin (10 mcg) was used as the positive control. Clear zone diameters were determined in millimeters following appropriate incubation (overnight at 37 °C) [47, 62]. 

### Minimum inhibitory concentration (MIC) evaluation

The MIC values were determined using the broth dilution method [63, 64]. A stock solution (10.24 mg/mL) of each investigated compound in dimethyl sulfoxide (DMSO) was produced and subsequently diluted to 1024 µg/mL using Mueller-Hinton broth. In Mueller-Hinton medium, the strains were briefly utilized at 37 °C. After 5 h of bacterial development and growth, the culture became diluted to a concentration of 5 × 105 cells per milliliter. Bacterial solutions (150 µL) have been added to each well of a 96-well tissue culture plate with flat bottom. Two-fold series of dilutions were performed from the first to tenth wells, with final drug concentrations ranging from 1 to 512 µg/mL. Excess medium (150 µL) was removed from the final well. The plates were visually examined after being incubated at 37 °C for 24 h in an electro-heating standing temperature cultivator. The MIC of the sample with no turbidity was determined as the lowest concentration of a substance that totally inhibited bacterial growth. Each assay was done in triplicate (Table 3).

**Table 3:** MIC of compounds **16b**,** 16c**,** 18b**,** 18d** against the sensitive microorganism

Compound	MIC (µg/ml)		
	G^−^	G^+^	Fungi
	*E. coli*	*B. mycoides*	*C. albicans*
16b	----	125	420
16c	175	132	250
18b	40	60	600
18d	154	102	----

Abbreviation: G^−^ = Gram negative; G^+^ = gram positive; MIC = minimum inhibitory concentration

### Structure–activity relationship

**Fig. 3:**
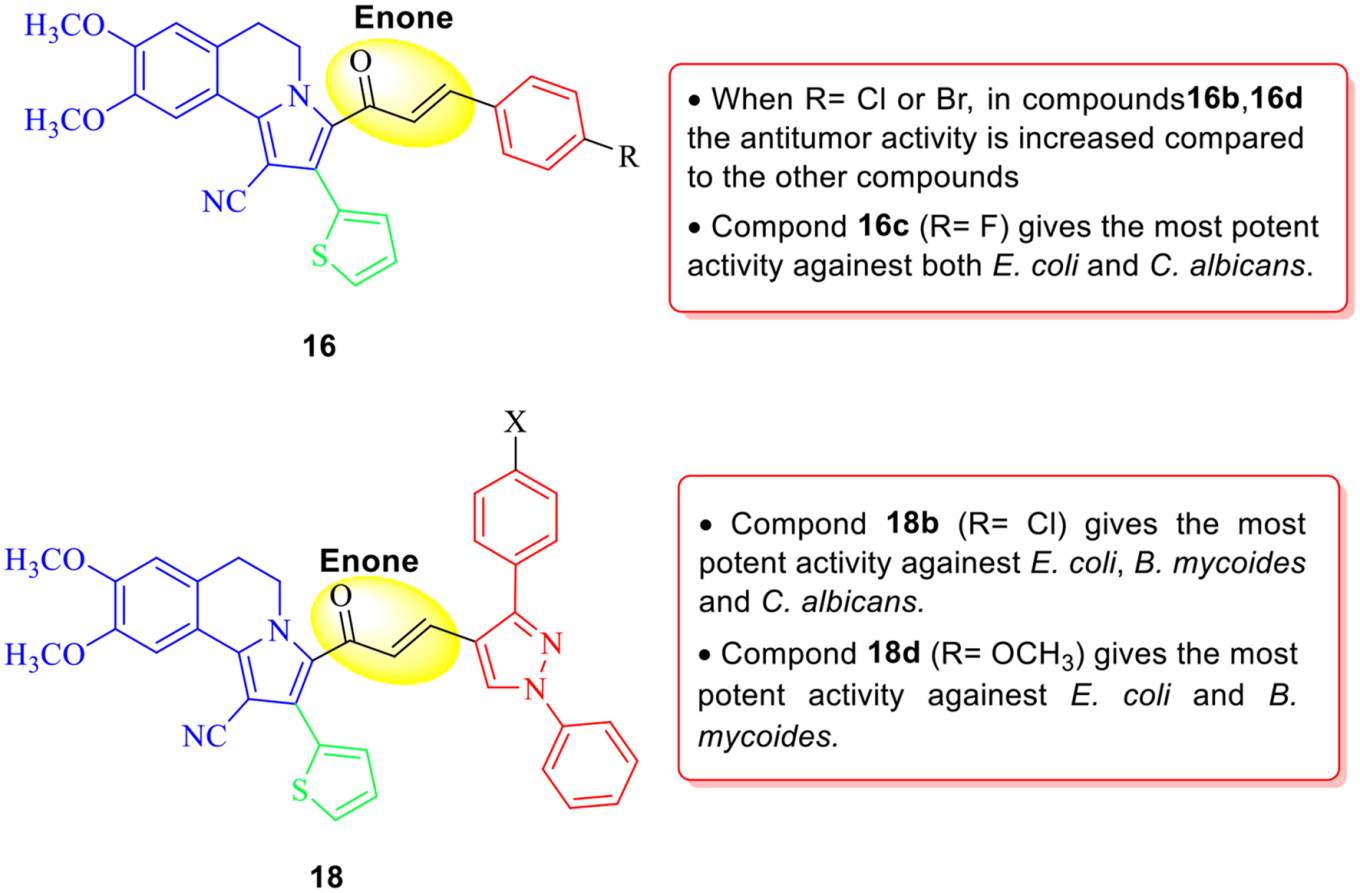
Structure–activity relationship (SAR) study of the prepared chalcones **16** and **18**

As shown in Fig. 3, the anticancer activity of the prepared chalcones **16** and **18** against MCF7 and A549 cancer cell lines increased when R = Cl and Br as shown in chalcones **16b** and **16d** as compared to the other chalcones. In addition, the presence of fluorine atom gave chalcone **16c** the most potant activity against both *E. coli* and *C. albicans*. Also, chalcone **18b** which contain a pyrazole moiety and chlorine atom has the most potant activity against *E. coli*, *B. mycoides* and *C. albicans*.

## Conclusion

In summary, synthesis of novel dihydropyrrolo[2,1-*a*] isoquinoline chalcones was reported using Claisen–Schmidt condensation. We could manage to confirm the constitutions of these chalcones using different spectral data. The antitumor activity against two human cancer cell lines, namely, MCF7 (human Caucasian breast adenocarcinoma) and A549 (lung carcinoma) were evaluated. The results showed that compounds **16b** and **16d** possess antitumor activities. The antimicrobial activity of the newly synthesized compounds revealed the most potent compounds **16b**, **16c**, **18b** and **18d**. Moreover, compound **18b** had the lowest MIC values.

**Supplementary file**.

The ^1^H and ^13^C NMR spectra of all new compounds are represented in the supplementary file.

“**Experimental**.

“The melting points were determined using an Electrothermal 9100 apparatus, and no corrections were applied. Infrared (IR) spectra were obtained with a Bruker Vector 22 FTIR spectrophotometer, utilizing KBr pellets. Nuclear Magnetic Resonance (NMR) spectra, including both ¹H and ¹³C, were recorded in CDCl₃ or DMSO-*d₆* as solvents on a Varian Gemini NMR spectrometer operating at 300 MHz and 75 MHz, respectively, with tetramethylsilane (TMS) serving as the internal reference. Chemical shifts were expressed as δ values in parts per million (ppm). Mass spectrometry analysis was conducted using a Shimadzu GCMS-QP-1000 EX spectrometer under Electron Ionization (EI) at 70 eV. Elemental analyses were carried out at the Microanalytical Center of Cairo University. 2-(6,7-Dimethoxy-3,4-dihydroisoquinolin-1-yl)acetonitrile **1** [52, 53], hydrazonoyl halides **4–7** [54, 55, 56, 57], and pyrazole aldehydes **17** [58] were prepared according to reported procedures”.

***Synthesis of 2-(6***,***7-dimethoxy-3***,***4-dihydroisoquinolin-1-yl)-3-(thiophen-2-yl)acrylonitrile hydrochloride*(3)**.

To a mixture of 2-(6,7-dimethoxy-3,4-dihydroisoquinolin-1-yl)acetonitrile **1** (11.5 g, 50 mmol) and thiophene-2-carbaldehyde **2** (5.6 g, 50 mmol) in absolute ethanol (100 mL), concentrated hydrochloric acid (10 mL) was added at room temperature. The reaction mixture was stirred for 6 h. The formed precipitate was collected and crystallized from *N*,*N*-dimethylformamide to afford arylidene **3**. Brown crystals; yield (78%); mp 209–211 ºC; IR (*KBr*) $\:\stackrel{-}{\nu\:}$ 2204 (CN) cm^− 1^; ^1^H NMR (300 MHz, DMSO–*d*_6_) *δ* 2.74–2.78 (m, 2 H, CH_2_), 3.81 (s, 3 H, OCH_3_), 3.82 (s, 3 H, OCH_3_), 3.87–3.95 (m, 2 H, CH_2_), 6.92–6.96 (m, 1H, Thienyl-H), 6.98 (s, 1H, Isoq-H), 7.16–8.14 (m, 4 H, Ar-H), 9.94 (s, 1H, N-HCl); ^13^C NMR (75 MHz, DMSO–*d*_6_) *δ* 26.4, 43.6, 55.9, 56.1, 99.1, 110.9, 114.3, 116.0, 122.0, 128.5, 129.0, 130.4, 134.7, 137.7, 147.0, 151.7, 152.2, 164.4; MS (EI, 70 eV) m/z (%): 360 (M^+^, 100). Anal. Calcd. For C_18_H_17_ClN_2_O_2_S (360.86): C, 59.91; H, 4.75; Cl, 9.82; N, 7.76; S, 8.88. Found: C, 59.80; H, 4.81; Cl, 9.90; N, 7.70; S, 8.95.

***Synthesis of 8***,***9-dimethoxy-2-(thiophen-2-yl)-5***,***6-dihydropyrrolo[2***,***1-a]isoquinoline-1-carbonitrile derivatives*** **(11–14)**.

To a mixture of 2-(6,7-dimethoxy-3,4-dihydroisoquinolin-1-yl)-3-(thiophen-2-yl)acrylonitrile hydrochloride **3** (0.72 g, 2 mmol) and *α*-ketohydrazonoyl halides **4–7** (2 mmol) in chloroform (20 mL), triethylamine (2 mL) was added at room temperature. The reaction mixture was refluxed for 6 h and then cooled, the excess chloroform was removed under reduced pressure and the residue was treated with ethanol (10 mL). The solid that precipitated was collected and crystallized from suitable solvent to give **11–14**. The compounds prepared with their physical data are listed below:

***3-Acetyl-8***,***9-dimethoxy-2-(thiophen-2-yl)-5***,***6-dihydropyrrolo[2***,***1-a]isoquinoline-1-carbonitrile*** **(11)**:

Light brown crystals; CH_3_CN; yield (75%); mp 221–223 °C; IR (*KBr*) $\:\stackrel{-}{\nu\:}$ 2212 (CN), 1634 (CO) cm^− 1^; ^1^H NMR (300 MHz, CDCl_3_) *δ* 2.12 (s, 3 H, COCH_3_), 3.01–3.05 (m, 2 H, CH_2_), 3.94 (s, 3 H, OCH_3_), 3.95 (s, 3 H, OCH_3_), 4.59–4.63 (m, 2 H, CH_2_), 6.78 (s, 1H, Isoq-H), 7.14–7.50 (m, 3 H, Thienyl-H), 7.80 (s, 1H, Isoq-H); ^13^C NMR (75 MHz, CDCl_3_) *δ* 28.1, 29.8, 43.3, 55.9, 56.0, 91.5, 107.7, 110.5, 116.1, 117.6, 126.6, 127.5, 127.8, 128.8, 129.0, 129.2, 132.3, 139.0, 148.2, 150.3, 190.5; MS (EI, 70 eV) m/z (%): 378 (M^+^, 100). Anal. Calcd. For C_21_H_18_N_2_O_2_S (378.45): C, 66.65; H, 4.79; N, 7.40; S, 8.47. Found: C, 66.58; H, 4.71; N, 7.48; S, 8.37.

***3-Benzoyl-8***,***9-dimethoxy-2-(thiophen-2-yl)-5***,***6-dihydropyrrolo[2***,***1-a]isoquinoline-1-carbonitrile*** **(12)**:

Golden crystals; DMF + EtOH; yield (84%); mp 259–261 °C; ^1^H NMR (300 MHz, CDCl_3_) *δ* 3.07–3.12 (m, 2 H, CH_2_), 3.96 (s, 3 H, OCH_3_), 4.00 (s, 3 H, OCH_3_), 4.39–4.44 (m, 2 H, CH_2_), 6.76–7.38 (m, 7 H, Ar-H), 7.66 (d, 2 H, Ar-H, *J* = 9 Hz), 7.90 (s, 1H, Isoq-H); ^13^C NMR (75 MHz, CDCl_3_) *δ* 28.2, 43.1, 55.9, 56.1, 89.0, 107.6, 110.7, 116.7, 117.9, 126.2, 127.0, 127.1, 127.4, 127.8, 127.9, 128.6, 129.5, 132.2, 132.7, 137.6, 139.3, 148.3, 150.1, 187.2; MS (EI, 70 eV) m/z (%): 440 (M^+^, 100). Anal. Calcd. For C_26_H_20_N_2_O_3_S (440.52): C, 70.89; H, 4.58; N, 6.36; S, 7.28. Found: C, 70.95; H, 4.50; N, 6.30; S, 7.17.

***8***,***9-Dimethoxy-2-(thiophen-2-yl)-3-(thiophene-2-carbonyl)-5***,***6-dihydropyrrolo[2***,***1-a]isoquinoline-1-carbonitrile*** **(13)**:

Light brown crystals; DMF + EtOH; yield (83%); mp 264–266 ºC; ^1^H NMR (300 MHz, CDCl_3_) *δ* 3.06–3.11 (m, 2 H, CH_2_), 3.95 (s, 3 H, OCH_3_), 3.99 (s, 3 H, OCH_3_), 4.32–4.36 (m, 2 H, CH_2_), 6.79 (s, 1H, Isoq-H), 6.82–6.85 (m, 1H, Thienyl-H), 6.90–6.92 (m, 1H, Thienyl-H), 7.09 (d, 1H, Thienyl-H, *J* = 5.1 Hz), 7.20 (d, 1H, Thienyl-H, *J* = 5.1), 7.33 (d, 1H, Thienyl-H, *J* = 4.8), 7.54 (d, 1H, Thienyl-H, *J* = 4.8), 7.89 (s, 1H, Isoq-H); ^13^C NMR (75 MHz, CDCl_3_) *δ* 28.3, 43.1, 56.0, 56.1, 88.8, 107.6, 110.8, 116.8, 118.0, 126.0, 126.1, 127.1, 127.3, 127.7, 128.0, 132.8, 134.6, 134.7, 139.1, 144.1, 148.4, 150.2, 161.2, 178.9; MS (EI, 70 eV) m/z (%): 446 (M^+^, 100). Anal. Calcd. for C_24_H_18_N_2_O_3_S_2_ (446.54): C, 64.56; H, 4.06; N, 6.27; S, 14.36. Found: C, 64.67; H, 4.12; N, 6.20; S, 14.44.

***3-(2-Naphthoyl)-8***,***9-dimethoxy-2-(thiophen-2-yl)-5***,***6-dihydropyrrolo[2***,***1-a]isoquinoline-1-carbonitrile*** **(14)**:

Yellow crystals; CH_3_CN; yield (78%); mp 219–221 °C; ^1^H NMR (300 MHz, CDCl_3_) *δ* 3.08–3.12 (m, 2 H, CH_2_), 3.95 (s, 3 H, OCH_3_), 4.00 (s, 3 H, OCH_3_), 4.40–4.44 (m, 2 H, CH_2_), 6.60–6.63 (m, 1H, Ar-H), 6.81 (s, 1H, Isoq-H), 6.93–7.92 (m, 9 H, Ar-H), 8.17 (s, 1H, Isoq-H); ^13^C NMR (75 MHz, CDCl_3_) *δ* 28.2, 43.1, 55.9, 56.0, 88.9, 107.5, 110.7, 116.8, 117.9, 124.6, 126.1, 126.4, 126.9, 127.0, 127.3, 127.4, 127.9, 128.0, 128.3, 128.4, 129.2, 131.8, 131.9, 132.3, 134.8, 135.1, 139.3, 148.3, 150.1, 186.9; MS (EI, 70 eV) m/z (%): 490 (M^+^, 100). Anal. Calcd. for C_30_H_22_N_2_O_3_S (490.58): C, 73.45; H, 4.52; N, 5.71; S, 6.54. Found: C, 73.38; H, 4.60; N, 5.60; S, 6.47.

***Synthesis of chalcone derivatives*** **(16a-f)** ***and*** **(18a-d)**.

To a stirred mixture of 3-acetyl-8,9-dimethoxy-2-(thiophen-2-yl)-5,6-dihydropyrrolo[2,1-*a*]isoquinoline-1-carbonitrile **3** (1 mmol) and the appropriate aryl aldehydes **15a-f** or pyrazole aldehydes **17a-d** (1 mmol) in ethanol (30 mL), sodium hydroxide solution 20% (10 mL) was added, the reaction mixture was stirred for 6 h at room temperature, and left overnight. The resulting solid product that precipitated was filtered, washed with water and crystallized from a suitable solvent to give the corresponding chalcone derivatives **16a-f** and **18a-d**. The compounds prepared with their physical and chemical properties as follow:

***3-Cinnamoyl-8***,***9-dimethoxy-2-(thiophen-2-yl)-5***,***6-dihydropyrrolo[2***,***1-a]isoquinoline-1-carbonitrile*(16a)**:

White crystals; DMF + EtOH; yield (77%); mp 204–206 °C; IR (*KBr*) $\:\stackrel{-}{\nu\:}$ 2210 (CN), 1652 (CO) cm^− 1^; ^1^H NMR (300 MHz, CDCl_3_) *δ* 3.06–3.11 (m, 2 H, CH_2_), 3.96 (s, 3 H, OCH_3_), 3.98 (s, 3 H, OCH_3_), 4.64–4.68 (m, 2 H, CH_2_), 6.70 (d, 1H, CH, *J* = 15.6 Hz), 6.80 (s, 1H, Isoq-H), 7.16–7.53 (m, 8 H, Ar-H), 7.59 (d, 1H, CH, *J* = 15.6 Hz), 7.86 (s, 1H, Isoq-H); ^13^C NMR (75 MHz, CDCl_3_) *δ* 28.2, 43.3, 56.0, 56.1, 90.6, 107.8, 110.7, 116.4, 117.8, 125.3, 126.7, 127.7, 128.1, 128.2, 128.7, 129.6, 129.9, 130.2, 132.5, 134.7, 139.5, 141.8, 145.2, 148.4, 150.4, 181.8; MS (EI, 70 eV) m/z (%): 466 (M^+^, 100). Anal. Calcd. for C_28_H_22_N_2_O_3_S (466.56): C, 72.08; H, 4.75; N, 6.00; S, 6.87. Found: C, 72.16; H, 4.64; N, 6.09; S, 6.79.

***3-(3-(4-Chlorophenyl)acryloyl)-8***,***9-dimethoxy-2-(thiophen-2-yl)-5***,***6-dihydropyrrolo[2***,***1-a]isoquinoline-1-carbonitrile*** **(16b)**:

Yellow crystals; CH_3_CN; yield (82%); mp 227–229 °C; ^1^H NMR (300 MHz, CDCl_3_) *δ* 3.05–3.09 (m, 2 H, CH_2_), 3.95 (s, 3 H, OCH_3_), 3.97 (s, 3 H, OCH_3_), 4.63–4.67 (m, 2 H, CH_2_), 6.63 (d, 1H, CH, *J* = 15.9 Hz), 6.80 (s, 1H, Isoq-H), 7.09–7.56 (m, 8 H, Ar-H), 7.84 (s, 1H, Isoq-H); ^13^C NMR (75 MHz, CDCl_3_) *δ* 28.1, 43.2, 55.9, 56.0, 90.6, 107.6, 110.5, 116.2, 117.6, 125.7, 126.6, 127.6, 128.1, 128.2, 128.9, 129.1, 129.6, 132.3, 133.1, 135.9, 139.5, 140.0, 145.2, 148.2, 150.3, 181.2; MS (EI, 70 eV) m/z (%): 500 (M^+^, 100), 501 (M^+^, 30). Anal. Calcd. for C_28_H_21_ClN_2_O_3_S (501.00): C, 67.13; H, 4.23; Cl, 7.08; N, 5.59; S, 6.40. Found: C, 67.02; H, 4.30; Cl, 7.16; N, 5.67; S, 6.33.

***3-(3-(4-Fluorophenyl)acryloyl)-8***,***9-dimethoxy-2-(thiophen-2-yl)-5***,***6-dihydropyrrolo[2***,***1-a]isoquinoline-1-carbonitrile*** **(16c)**:

Orange crystals; DMF + EtOH; yield (84%); mp 220–222 °C; ^1^H NMR (300 MHz, CDCl_3_) *δ* 3.06–3.10 (m, 2 H, CH_2_), 3.96 (s, 3 H, OCH_3_), 3.98 (s, 3 H, OCH_3_), 4.63–4.68 (m, 2 H, CH_2_), 6.60 (d, 1H, CH, *J* = 15.6 Hz), 6.80 (s, 1H, Isoq-H), 6.96–7.59 (m, 8 H, Ar-H), 7.85 (s, 1H, Isoq-H); ^13^C NMR (75 MHz, CDCl_3_) *δ* 28.4, 43.1, 55.7, 56.2, 90.7, 107.6, 110.4, 116.2, 117.6, 124.6, 126.5, 127.6, 127.7, 127.8, 128.3, 129.5, 129.7, 130.0, 131.7, 132.3, 139.1, 140.6, 141.6, 148.1, 150.5, 181.6; MS (EI, 70 eV) m/z (%): 484 (M^+^, 100). Anal. Calcd. for C_28_H_21_FN_2_O_3_S (484.55): C, 69.41; H, 4.37; F, 3.92; N, 5.78: S, 6.62. Found: C, 69.34; H, 4.30; F, 3.81; N, 5.70; S, 6.55.

***3-(3-(4-Bromophenyl)acryloyl)-8***,***9-dimethoxy-2-(thiophen-2-yl)-5***,***6-dihydropyrrolo[2***,***1-a]isoquinoline-1-carbonitrile*** **(16d)**:

Orange crystals; DMF; yield (80%); mp 243–245 °C; ^1^H NMR (300 MHz, CDCl_3_) *δ* 3.05–3.10 (m, 2 H, CH_2_), 3.95 (s, 3 H, OCH_3_), 3.97 (s, 3 H, OCH_3_), 4.63–4.67 (m, 2 H, CH_2_), 6.65 (d, 1H, CH, *J* = 15.6 Hz), 6.80 (s, 1H, Isoq-H), 7.02 (d, 2 H, Ar-H, *J* = 8.1 Hz), 7.15–7.54 (m, 6 H, Ar-H), 7.85 (s, 1H, Isoq-H); ^13^C NMR (75 MHz, CDCl_3_) *δ* 28.4, 43.1, 55.9, 56.2, 90.8, 107.6, 110.8, 116.6, 117.6, 125.1, 126.8, 127.5, 128.3, 128.4, 128.6, 129.5, 129.9, 130.1, 132.7, 134.8, 139.4, 141.9, 145.4, 148.2, 150.6, 181.6; MS (EI, 70 eV) m/z (%): 544 (100), 545 (M^+^, 97). Anal. Calcd. for C_28_H_21_BrN_2_O_3_S (545.45): C, 61.66; H, 3.88; Br, 14.65; N, 5.14; S, 5.88. Found: C, 61.59; H, 3.80; Br, 14.53; N, 5.06; S, 5.81.

***8***,***9-Dimethoxy-3-(3-(4-nitrophenyl)acryloyl)-2-(thiophen-2-yl)-5***,***6-dihydropyrrolo[2***,***1-a]isoquinoline-1-carbonitrile*** **(16e)**:

Red crystals; CH_3_CN; yield (83%); mp 227–229 °C; ^1^H NMR (300 MHz, CDCl_3_) *δ* 3.08–3.12 (m, 2 H, CH_2_), 3.96 (s, 3 H, OCH_3_), 3.98 (s, 3 H, OCH_3_), 4.66–4.71 (m, 2 H, CH_2_), 6.76–6.81 (m, 2 H, Ar-H), 7.15–7.32 (m, 4 H, Ar-H), 7.57 (d, 2 H, Ar-H, *J* = 15.6 Hz), 7.86 (s, 1H, Isoq-H), 8.14 (d, 2 H, Ar-H, *J* = 8.7 Hz); ^13^C NMR (75 MHz, CDCl_3_) *δ* 28.2, 43.4, 55.7, 55.9, 90.4, 107.7, 110.7, 116.1, 117.4, 125.8, 126.4, 127.8, 128.2, 128.4, 128.8, 129.2, 129.8, 132.2, 133.3, 135.7, 139.4, 139.8, 145.4, 148.1, 150.1, 181.4; MS (EI, 70 eV) m/z (%): 511 (M^+^, 100). Anal. Calcd. For C_28_H_21_N_3_O_5_S (511.55): C, 65.74; H, 4.14; N, 8.21; S, 6.27. Found: C, 65.66; H, 4.26; N, 8.14; S, 6.20.

***8***,***9-Dimethoxy-2-(thiophen-2-yl)-3-(3-(p-tolyl)-acryloyl)-5***,***6-dihydropyrrolo[2***,***1-a]isoquinoline-1-carbonitrile*** **(16f)**:

Orange crystals; DMF + EtOH; yield (78%); mp 262–264 °C; ^1^H NMR (300 MHz, CDCl_3_) *δ* 2.35 (s, 3 H, CH_3_), 3.06–3.10 (m, 2 H, CH_2_), 3.96 (s, 3 H, OCH_3_), 3.98 (s, 3 H, OCH_3_), 4.63–4.67 (m, 2 H, CH_2_), 6.66 (d, 1H, CH, *J* = 15.6 Hz), 6.80 (s, 1H, Isoq-H), 7.11–7.51 (m, 7 H, Ar-H), 7.57 (d, 1H, CH, *J* = 15.6 Hz), 7.86 (s, 1H, Isoq-H); ^13^C NMR (75 MHz, CDCl_3_) *δ* 21.4, 28.2, 43.2, 55.9, 56.1, 90.5, 107.7, 110.6, 116.4, 117.8, 124.4, 126.6, 127.6, 127.8, 127.9, 128.1, 129.4, 129.5, 129.9, 131.9, 132.5, 139.3, 140.7, 141.8, 148.3, 150.3, 181.8; MS (EI, 70 eV) m/z (%): 480 (M^+^, 100). Anal. Calcd. for C_29_H_24_N_2_O_3_S (480.58): C, 72.48; H, 5.03; N, 5.83; S, 6.67. Found: C, 72.40; H, 5.15; N, 5.76; S, 6.74.

***3-(3-(1***,***3-Diphenyl-1 H-pyrazol-4-yl)acryloyl)-8***,***9-dimethoxy-2-(thiophen-2-yl)-5***,***6-dihydropyrrolo[2***,***1-a]isoquinoline-1-carbonitrile*** **(18a)**:

Yellow crystals; DMF + EtOH; yield (81%); mp 257–259 °C; ^1^H NMR (300 MHz, CDCl_3_) *δ* 3.17–3.19 (m, 2 H, CH_2_), 3.96 (s, 3 H, OCH_3_), 3.99 (s, 3 H, OCH_3_), 4.71–4.79 (m, 2 H, CH_2_), 6.65 (d, 1H, CH, *J* = 9 Hz), 6.72 (s, 1H, Isoq-H), 7.11 (d, 1H, CH, *J* = 9 Hz), 7.25–7.84 (m, 14 H, Ar-H), 7.97 (s, 1H, Isoq-H); ^13^C NMR (75 MHz, CDCl_3_) *δ* 28.4, 43.5, 56.0, 56.2, 90.3, 107.5, 110.8, 114.3, 116.5, 117.7, 117.9, 118.7, 124.6, 124.9, 126.4, 126.7, 126.9, 127.6, 127.8, 127.9, 129.6, 129.8, 129.9, 132.6, 133.2, 139.4, 139.5, 148.6, 150.4, 153.7, 156.4, 159.9, 181.6; MS (EI, 70 eV) m/z (%): 608 (M^+^, 100). Anal. Calcd. for C_37_H_28_N_4_O_3_S (608.72): C, 73.01; H, 4.64; N, 9.20; S, 5.27. Found: C, 73.10; H, 4.53; N, 9.27; S, 5.20.

***3-(3-(3-(4-Chlorophenyl)-1-phenyl-1 H-pyrazol-4-yl)acryloyl)-8***,***9-dimethoxy-2-(thiophen-2-yl)-5***,***6-dihydropyrrolo[2***,***1-a]isoquinoline-1-carbonitrile*** **(18b)**:

Yellow crystals; DMF; yield (82%); mp 262–264 °C; ^1^H NMR (300 MHz, CDCl_3_) *δ* 3.05–3.10 (m, 2 H, CH_2_), 3.95 (s, 3 H, OCH_3_), 3.98 (s, 3 H, OCH_3_), 4.64–4.68 (m, 2 H, CH_2_), 6.53 (d, 1H, CH, *J* = 15.6 Hz), 6.80 (s, 1H, Isoq-H), 7.22–7.70 (m, 14 H, Ar-H), 7.84 (s, 1H, Isoq-H); ^13^C NMR (75 MHz, CDCl_3_) *δ* 28.4, 43.1, 55.8, 56.4, 90.5, 107.8, 110.5, 116.3, 117.6, 118.3, 118.8, 124.9, 126.4, 126.5, 127.1, 127.4, 127.5, 127.8, 128.3, 129.1, 129.2, 129.3, 129.4, 129.7, 132.7, 133.0, 138.7, 139.3, 139.5, 148.2, 150.4, 153.4, 181.7; MS (EI, 70 eV) m/z (%): 642 (100), 643 (M^+^, 40). Anal. Calcd. for C_37_H_27_ClN_4_O_3_S (643.16): C, 69.10; H, 4.23; Cl, 5.51; N, 8.71; S, 4.98. Found: C, 69.18; H, 4.15; Cl, 5.43; N, 8.80; S, 4.86.

***8***,***9-Dimethoxy-3-(3-(1-phenyl-3-(p-tolyl)-1 H-pyrazol-4-yl)acryloyl)-2-(thiophen-2-yl)-5***,***6-dihydropyrrolo[2***,***1-a]isoquinoline-1-carbonitrile*** **(18c)**:

Orange crystals; DMF; yield (79%); mp 254–256 °C; ^1^H NMR (300 MHz, CDCl_3_) *δ* 2.42 (s, 3 H, CH_3_), 3.04–3.09 (m, 2 H, CH_2_), 3.95 (s, 3 H, OCH_3_), 3.98 (s, 3 H, OCH_3_), 4.62–4.67 (m, 2 H, CH_2_), 6.54 (d, 1H, CH, *J* = 15.6 Hz), 6.79 (s, 1H, Isoq-H), 7.22–7.71 (m, 14 H, Ar-H), 7.85 (s, 1H, Isoq-H); ^13^C NMR (75 MHz, CDCl_3_) *δ* 21.3, 28.2, 43.2, 56.0, 56.1, 90.5, 107.7, 110.6, 116.4, 117.8, 118.1, 119.0, 124.9, 126.1, 126.6, 127.0, 127.5, 127.7, 127.9, 128.4, 129.1, 129.4, 129.5, 129.6, 129.8, 132.5, 133.1, 138.4, 139.2, 139.3, 148.3, 150.3, 153.6, 181.5; MS (EI, 70 eV) m/z (%): 622 (M^+^, 100). Anal. Calcd. for C_38_H_30_N_4_O_3_S (622.74): C, 73.29; H, 4.86; N, 9.00; S, 5.15. Found: C, 73.20; H, 4.79; N, 9.08; S, 5.04.

***8***,***9-Dimethoxy-3-(3-(3-(4-methoxyphenyl)-1-phenyl-1 H-pyrazol-4-yl)acryloyl)-2-(thiophen-2-yl)-5***,***6-dihydropyrrolo[2***,***1-a]isoquinoline-1-carbonitrile*** **(18d)**:

Yellow crystals; DMF; yield (76%); mp 257–259 °C; ^1^H NMR (300 MHz, CDCl_3_) *δ* 3.05–3.09 (m, 2 H, CH_2_), 3.87 (s, 3 H, OCH_3_), 3.95 (s, 3 H, OCH_3_), 3.98 (s, 3 H, OCH_3_), 4.63–4.68 (m, 2 H, CH_2_), 6.54 (d, 1H, CH, *J* = 15.6 Hz), 6.79 (s, 1H, Isoq-H), 6.99 (d, 1H, CH, *J* = 8.7 Hz), 7.23–7.71 (m, 13H, Ar-H), 7.85 (s, 1H, Isoq-H); ^13^C NMR (75 MHz, CDCl_3_) *δ* 28.2, 43.2, 55.3, 55.9, 56.1, 90.5, 107.6, 110.6, 114.1, 116.4, 117.8, 118.0, 118.9, 124.5, 124.8, 126.1, 126.6, 127.0, 127.5, 127.7, 127.8, 129.5, 129.6, 129.7, 129.9, 132.5, 133.1, 139.2, 139.3, 148.3, 150.3, 153.3, 159.9, 181.4; MS (EI, 70 eV) m/z (%): 638 (M^+^, 100). Anal. Calcd. for C_38_H_30_N_4_O_4_S (638.74): C, 71.46; H, 4.73; N, 8.77; S, 5.02. Found: C, 71.52; H, 4.65; N, 8.66; S, 5.10.

***Synthesis of 8***,***9-dimethoxy-2-(thiophen-2-yl)-3-(5-(p-tolyl)-4***,***5-dihydro-1 H-pyrazol-3-yl)-5***,***6-dihydropyrrolo[2***,***1-a]isoquinoline-1-carbonitrile*** **(19)**.

A mixture of 8,9-dimethoxy-2-(thiophen-2-yl)-3-(3-(*p*-tolyl)acryloyl)-5,6-dihydropyrrolo[2,1-*a*]isoquinoline-1-carbonitrile **16f** (0.48 g, 1 mmol) and hydrazine hydrate (0.21 mL, 1 mmol) in ethanol (10 mL) was refluxed for 30 min. The reaction mixture was cooled and the solid formed was collected, washed with ethanol and crystallized from acetonitrile to give the corresponding pyrazoline derivative **19**. Orange crystals; yield (75%); mp 209–211 °C; IR (*KBr*) $\:\stackrel{-}{\nu\:}$ 3314 (NH), 2202 (CN) cm^− 1^; ^1^H NMR (300 MHz, CDCl_3_) *δ* 2.35 (s, 3 H, CH_3_), 2.58–2.67 (m, 1H, CH-Pyrazoline), 2.95–3.01 (m, 1H, CH-Pyrazoline), 3.02–3.07 (m, 2 H, CH_2_), 3.94 (s, 3 H, OCH_3_), 3.97 (s, 3 H, OCH_3_), 4.52–4.57 (m, 2 H, CH_2_), 4.69–4.75 (m, 1H, CH-Pyrazoline), 6.78 (s, 1H, Isoq-H), 6.99–7.35 (m, 7 H, Ar-H), 7.81 (s, 1H, Isoq-H), 9.49 (s, 1H, NH); ^13^C NMR(75 MHz, CDCl_3_) *δ* 20.9, 28.3, 42.6, 43.0, 55.8, 55.9, 63.5, 89.4, 107.0, 110.5, 117.0, 118.6, 121.4, 124.1, 125.3, 126.6, 127.0, 127.6, 128.1, 128.3, 129.2, 132.7, 137.3, 138.5, 143.9, 148.1, 149.3; MS (EI, 70 eV) m/z (%): 494 (M^+^, 100). Anal. Calcd. for C_29_H_26_N_4_O_2_S (494.61): C, 70.42; H, 5.30; N, 11.33; S, 6.48. Found: C, 70.35; H, 5.38; N, 11.22; S, 6.40.

***Synthesis of 3-(1-cyano-8***,***9-dimethoxy-2-(thiophen-2-yl)-5***,***6-dihydropyrrolo[2***,***1-a]isoquinolin-3-yl)-N-phenyl-5-(p-tolyl)-4***,***5-dihydro-1 H-pyrazole-1-carbothioamide*** **(20)**.

To a solution of 8,9-dimethoxy-2-(thiophen-2-yl)-3-(5-(*p*-tolyl)-4,5-dihydro-1*H*-pyrazol-3-yl)-5,6-dihydropyrrolo[2,1-*a*]isoquinoline-1-carbonitrile **19** (0.49 g, 1 mmol) in dry ether (10 mL), phenyl isothiocyanate (0.135 g, 1 mmol) was added. The reaction mixture was stirred for 5 h. The solid product separated was collected, washed with ethanol and crystallized from *N*,*N*-dimethylformamide to give compound **20**. White crystals; yield (81%); mp 229–231 °C; IR (*KBr*) $\:\stackrel{-}{\nu\:}$ 3530 (NH), 2208 (CN) cm^− 1^; ^1^H NMR (300 MHz, CDCl_3_) *δ* 2.33 (s, 3 H, CH_3_), 2.67–2.74 (m, 1H, CH-Pyrazoline), 3.10–3.15 (m, 2 H, CH_2_), 3.41–3.51 (m, 1H, CH-Pyrazoline), 3.96 (s, 3 H, OCH_3_), 3.97 (s, 3 H, OCH_3_), 4.51–4.59 (m, 2 H, CH_2_), 5.94–5.99 (m, 1H, CH-Pyrazoline), 6.80 (s, 1H, Isoq-H), 7.00-7.63 (m, 12 H, Ar-H), 7.81 (s, 1H, Isoq-H), 9.00 (s, 1H, NH); ^13^C NMR (75 MHz, CDCl_3_) *δ* 21.0, 28.3, 43.4, 43.9, 56.0, 56.1, 62.2, 91.1, 107.4, 110.7, 116.2, 118.0, 120.0, 122.3, 124.1, 125.3, 125.4, 125.5, 127.3, 127.6, 128.5, 129.1, 129.3, 131.4, 137.2, 138.0, 138.5, 138.7, 147.7, 148.4, 150.1, 173.6; MS (EI, 70 eV) m/z (%): 629 (M^+^, 100). Anal. Calcd. for C_36_H_31_N_5_O_2_S_2_ (629.80): C, 68.66; H, 4.96; N, 11.12; S, 10.18. Found: C, 68.59; H, 4.87; N, 11.24; S, 10.10.

***Synthesis of 3-(5-(p-tolyl)-4***,***5-dihydro-1 H-pyrazol-3-yl)-8***,***9-dimethoxy-2-(thiophen-2-yl)-5***,***6-dihydropyrrolo[2***,***1-a]isoquinoline-1-carbonitrile derivatives*** **(21 and 22)**.

Reflux of 8,9-dimethoxy-2-(thiophen-2-yl)-3-(5-(*p*-tolyl)-4,5-dihydro-1*H*-pyrazol-3-yl)-5,6-dihydropyrrolo[2,1-*a*]isoquinoline-1-carbonitrile **19** (0.49 g, 1 mmol) in acetic anhydride or formic acid (10 mL) for 30 min and the mixture was cooled, diluted with water and the resulting solid product was collected and crystallized from ethanol to give compounds **21** and **22**.

***3-(1-Acetyl-5-(p-tolyl)-4***,***5-dihydro-1 H-pyrazol-3-yl)-8***,***9-dimethoxy-2-(thiophen-2-yl)-5***,***6-dihydropyrrolo[2***,***1-a]isoquinoline-1-carbonitrile*** **(21)**:

Green crystals; yield (80%); mp 216–218 °C; IR (*KBr*) $\:\stackrel{-}{\nu\:}$ 2189 (CN), 1657 (CO) cm^− 1^; ^1^H NMR (300 MHz, CDCl_3_) *δ* 2.31 (s, 3 H, CH_3_), 2.37 (s, 3 H, COCH_3_), 2.64–2.72 (m, 1H, CH-Pyrazoline), 3.09–3.13 (m, 2 H, CH_2_), 3.26–3.35 (m, 1H, CH-Pyrazoline), 3.96 (s, 3 H, OCH_3_), 3.97 (s, 3 H, OCH_3_), 4.61–4.67 (m, 2 H, CH_2_), 5.32–5.35 (m, 1H, CH-Pyrazoline), 6.80 (s, 1H, Isoq-H), 7.01–7.38 (m, 7 H, Ar-H), 7.81 (s, 1H, Isoq-H); ^13^C NMR (75 MHz, CDCl_3_) *δ* 20.8, 21.8, 28.2, 43.3, 43.4, 55.8, 55.9, 58.5, 90.6, 107.1, 110.5, 116.3, 118.1, 122.8, 124.1, 125.3, 125.5, 127.0, 127.2, 129.0, 129.1, 131.7, 137.1, 137.9, 138.1, 146.5, 148.1, 149.8, 168.0; MS (EI, 70 eV) m/z (%): 536 (M^+^, 100). Anal. Calcd. for C_31_H_28_N_4_O_3_S (536.65): C, 69.38; H, 5.26; N, 10.44; S, 5.97. Found: C, 69.30; H, 5.19; N, 10.32; S, 5.90.

***3-(1-Formyl-5-(p-tolyl)-4***,***5-dihydro-1 H-pyrazol-3-yl)-8***,***9-dimethoxy-2-(thiophen-2-yl)-5***,***6-dihydropyrrolo[2***,***1-a]isoquinoline-1-carbonitrile*** **(22)**:

White crystals; yield (82%); mp 237–239 °C;; IR (*KBr*) $\:\stackrel{-}{\nu\:}$ 2195 (CN), 1659 (CO) cm^− 1^; ^1^H NMR (300 MHz, CDCl_3_) *δ* 2.32 (s, 3 H, CH_3_), 2.69–2.76 (m, 1H, CH-Pyrazoline), 3.09–3.13 (m, 2 H, CH_2_), 3.29–3.39 (m, 1H, CH-Pyrazoline), 3.96 (s, 3 H, OCH_3_), 3.97 (s, 3 H, OCH_3_), 4.64–4.67 (m, 2 H, CH_2_), 5.24–5.30 (m, 1H, CH-Pyrazoline), 6.81 (s, 1H, Isoq-H), 7.02–7.38 (m, 7 H, Ar-H), 7.81 (s, 1H, Isoq-H), 8.92 (s, 1H, CHO); ^13^C NMR (75 MHz, CDCl_3_) *δ* 20.9, 28.2, 43.6, 43.7, 55.9, 56.0, 57.7, 91.0, 107.3, 110.6, 116.3, 118.1, 122.5, 124.7, 125.6, 125.7, 127.2, 127.4, 129.2, 129.4, 131.7, 136.9, 137.6, 138.4, 148.3, 148.5, 150.0, 159.6; MS (EI, 70 eV) m/z (%): 522 (M^+^, 100). Anal. Calcd. for C_30_H_26_N_4_O_3_S (522.62): C, 68.95; H, 5.01; N, 10.72; S, 6.13. Found: C, 68.87; H, 5.08; N, 10.83; S, 6.06.

## Electronic supplementary material

Below is the link to the electronic supplementary material.

Supplementary Material 1

## Data Availability

All data generated or analyzed during this study are included in this published article and its supplementary information file.
