# Supplementary material for: Synthesis, reactions, antitumor and antimicrobial activity of new 5,6-dihydropyrrolo[2,1-a]isoquinoline chalcones
Source: BMC Chem. 2025 Jul 8;19(1):204. doi: 10.1186/s13065-025-01557-4 (PMC12239273; doi:10.1186/s13065-025-01557-4)

**Supplementary File**

**Synthesis, Reactions, Antitumor and Antimicrobial Activity of New 5,6-Dihydropyrrolo[2,1-*a*]isoquinoline Chalcones**

Mohamed A. Mohamed Teleb, Monica G. Kamel, Madonna S. Mikhail, Hamdi M. Hassaneen*, Ayman W. Erian*, Mirna T. Helmy

Department of Chemistry, Faculty of Science, Cairo University, Giza 12613, Egypt.

Hamdi M Hassaneen, [hhassaneen@sci.cu.edu.eg](mailto:hhassaneen@sci.cu.edu.eg), [hamdi_251@yahoo.com](mailto:hamdi_251@yahoo.com)*.*

Ayman W Erian, [erian11@hotmail.com](mailto:erian11@hotmail.com)


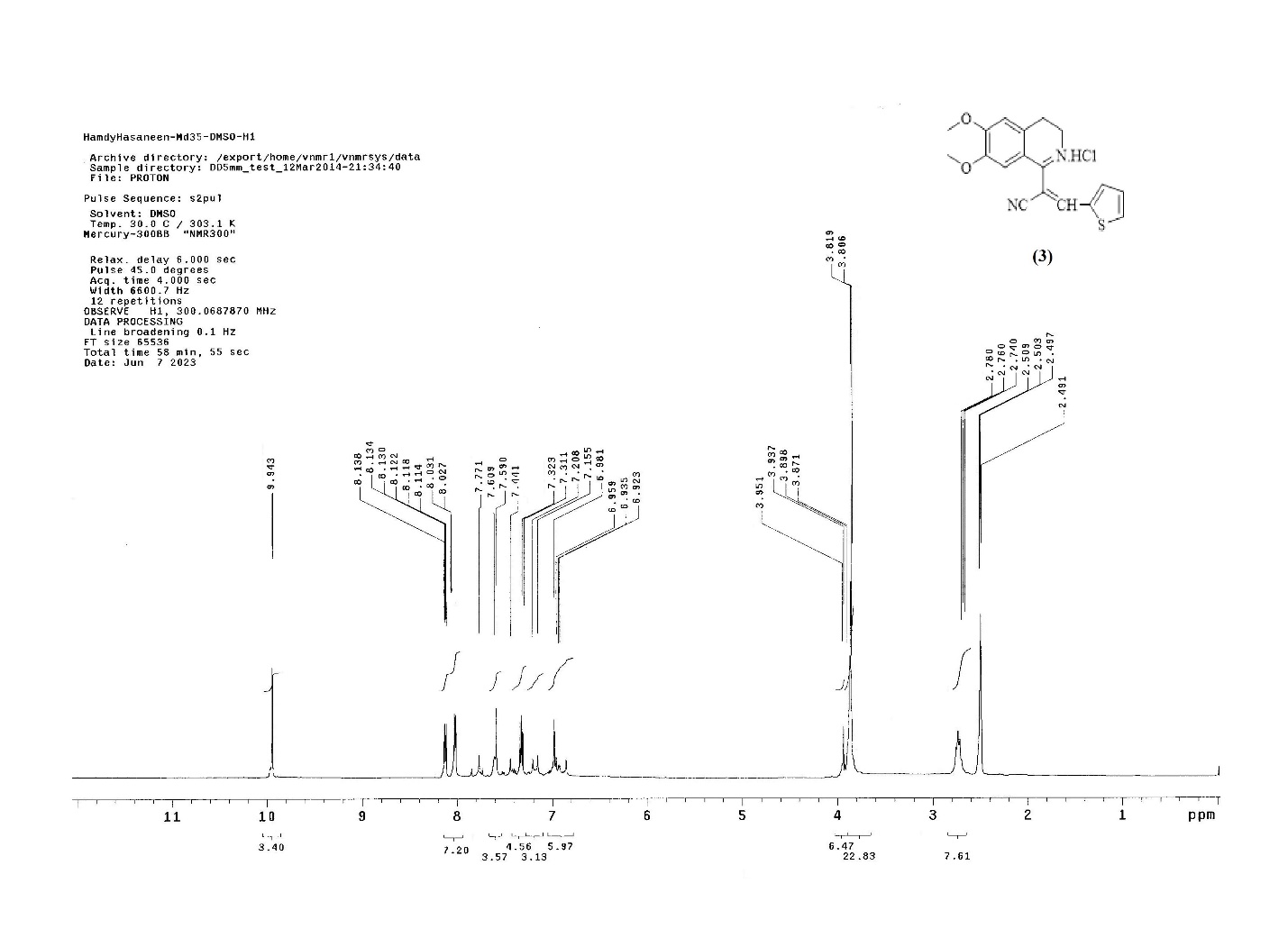


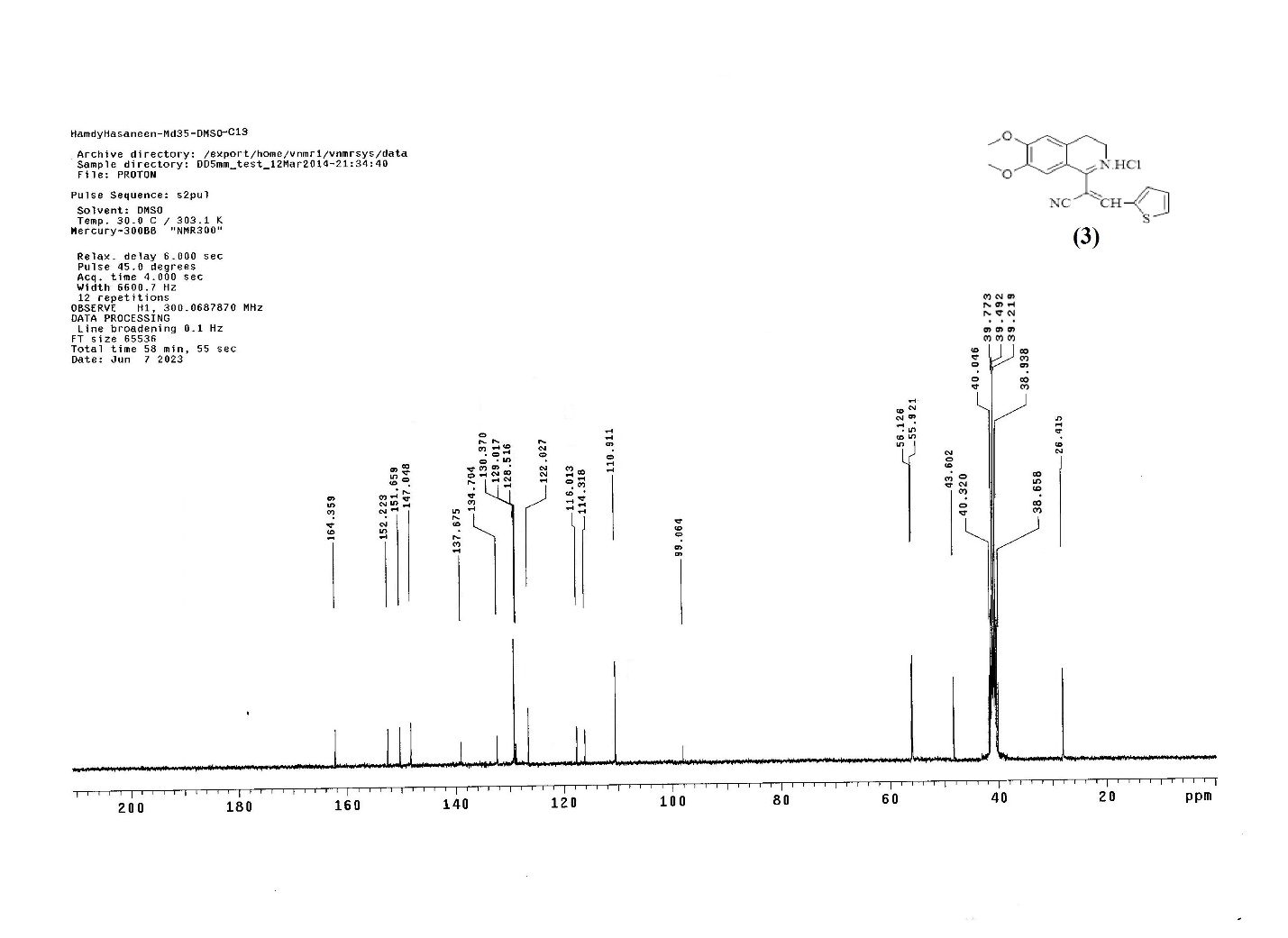


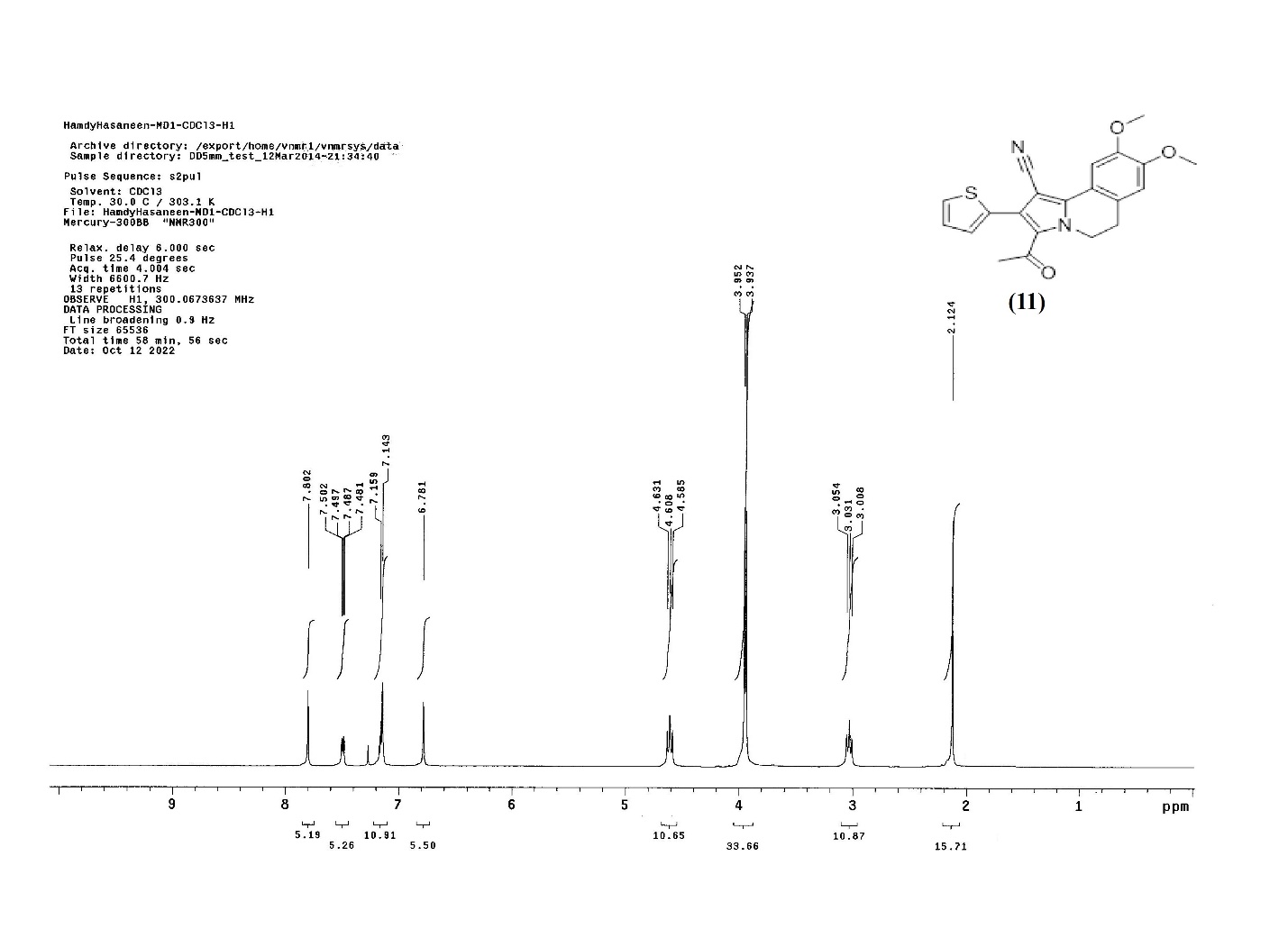

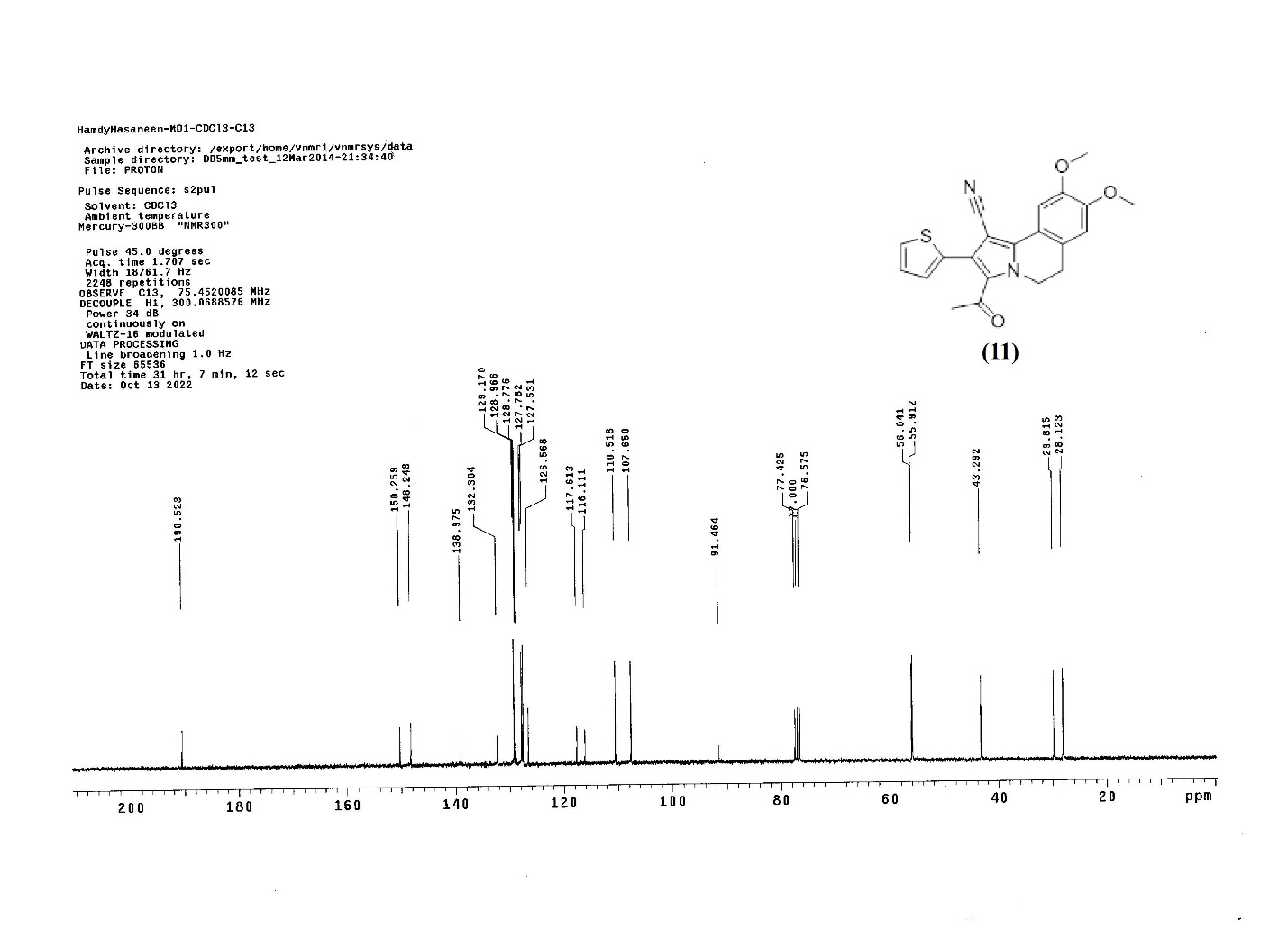

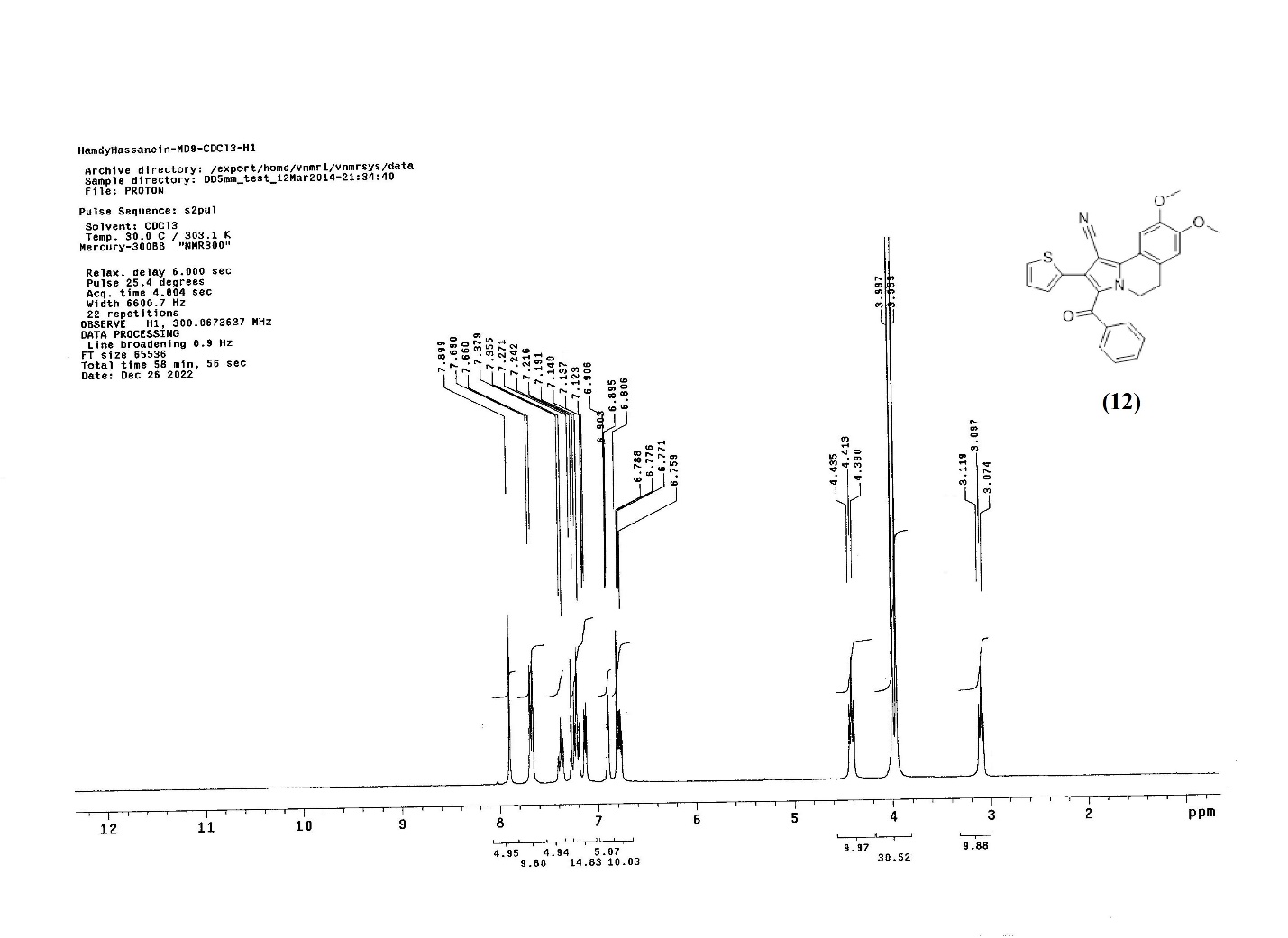

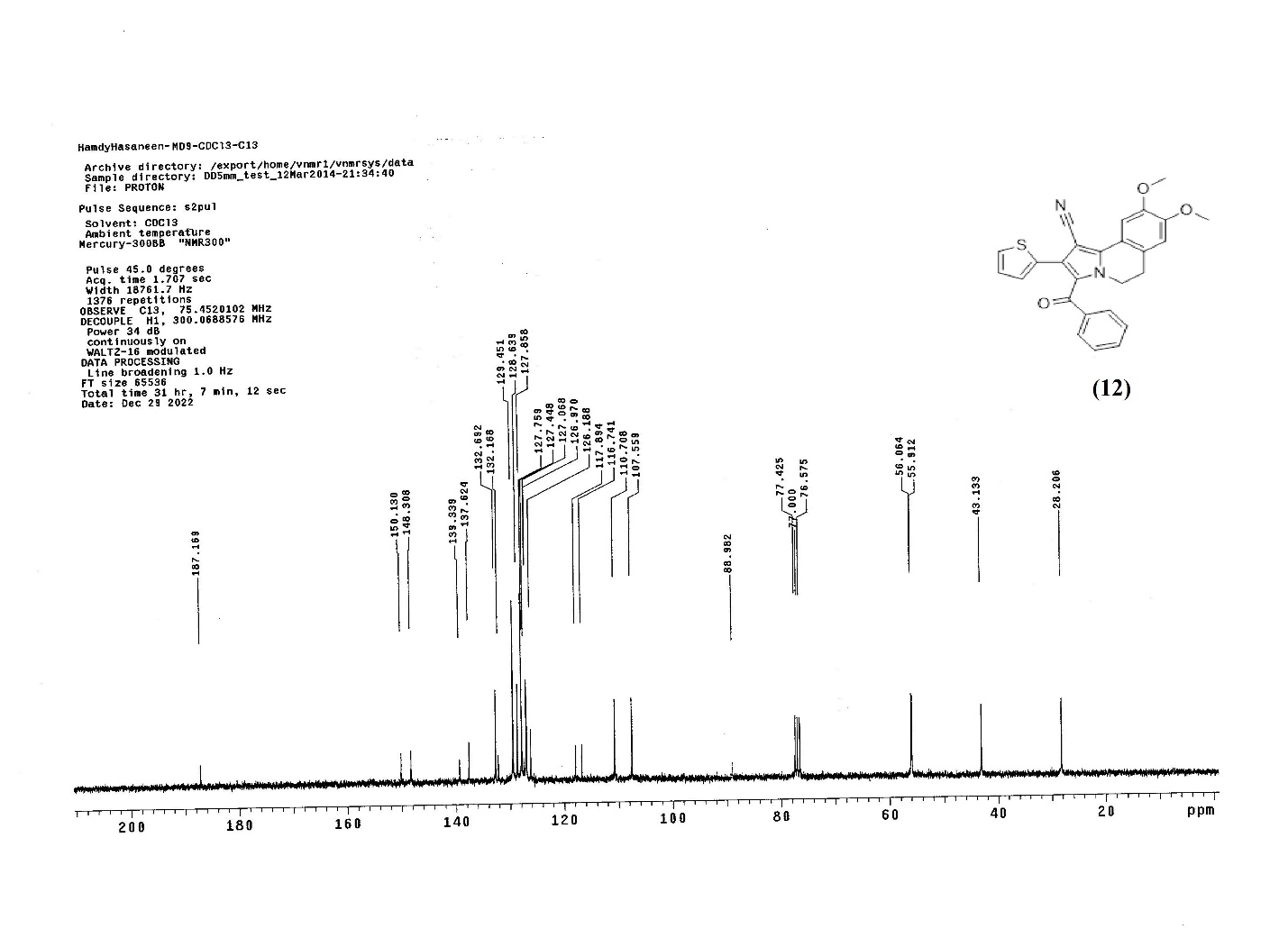


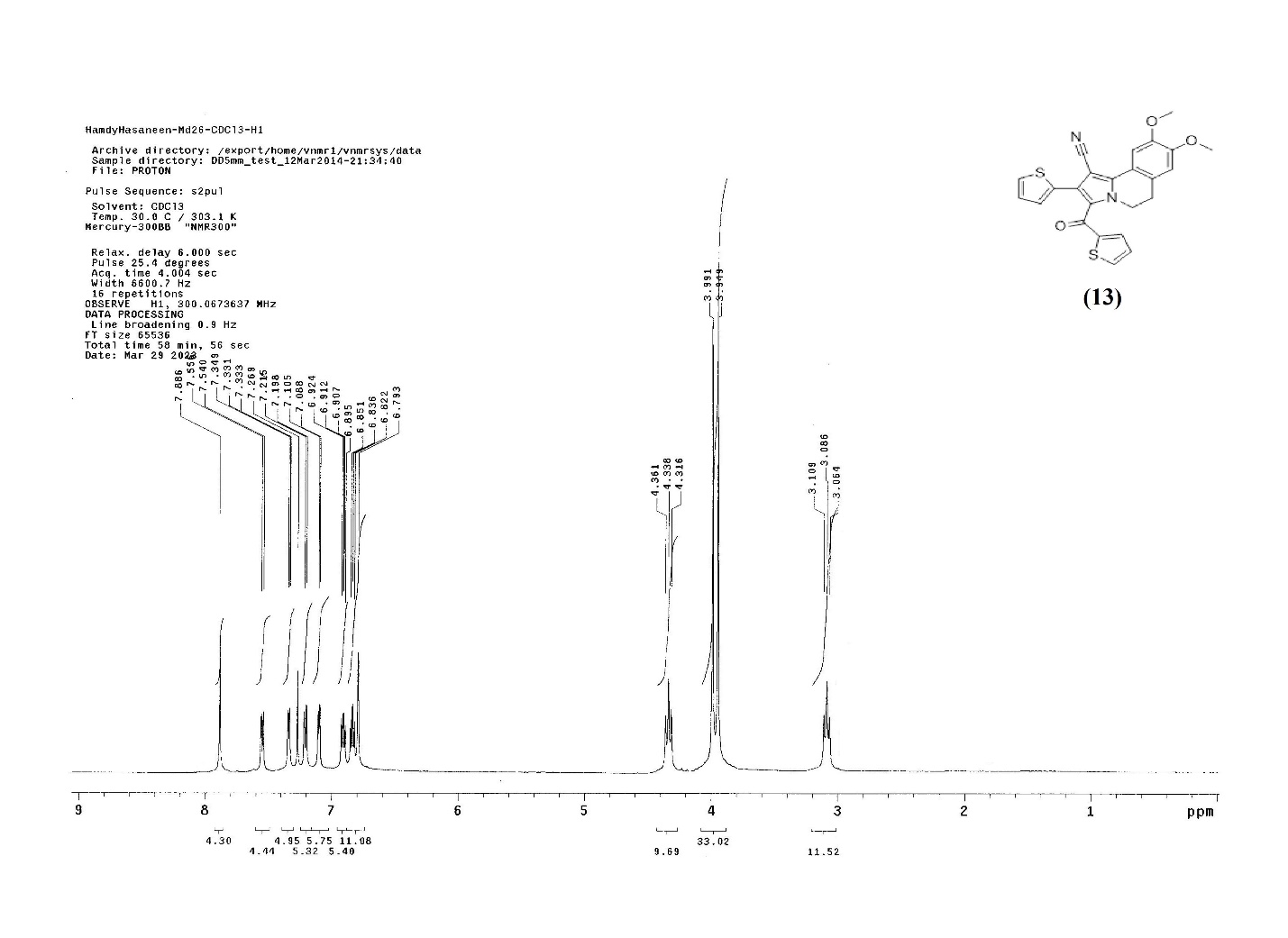

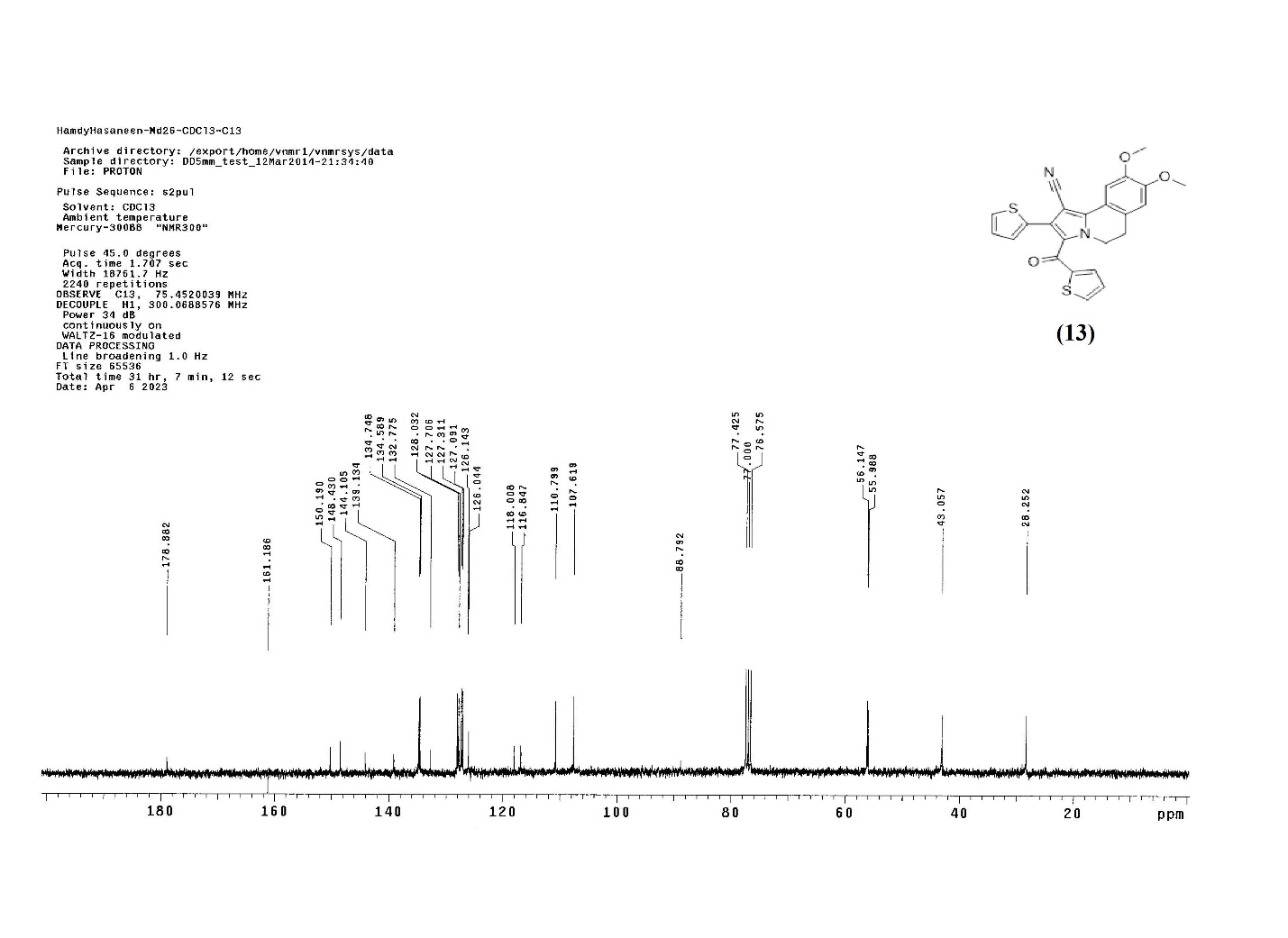

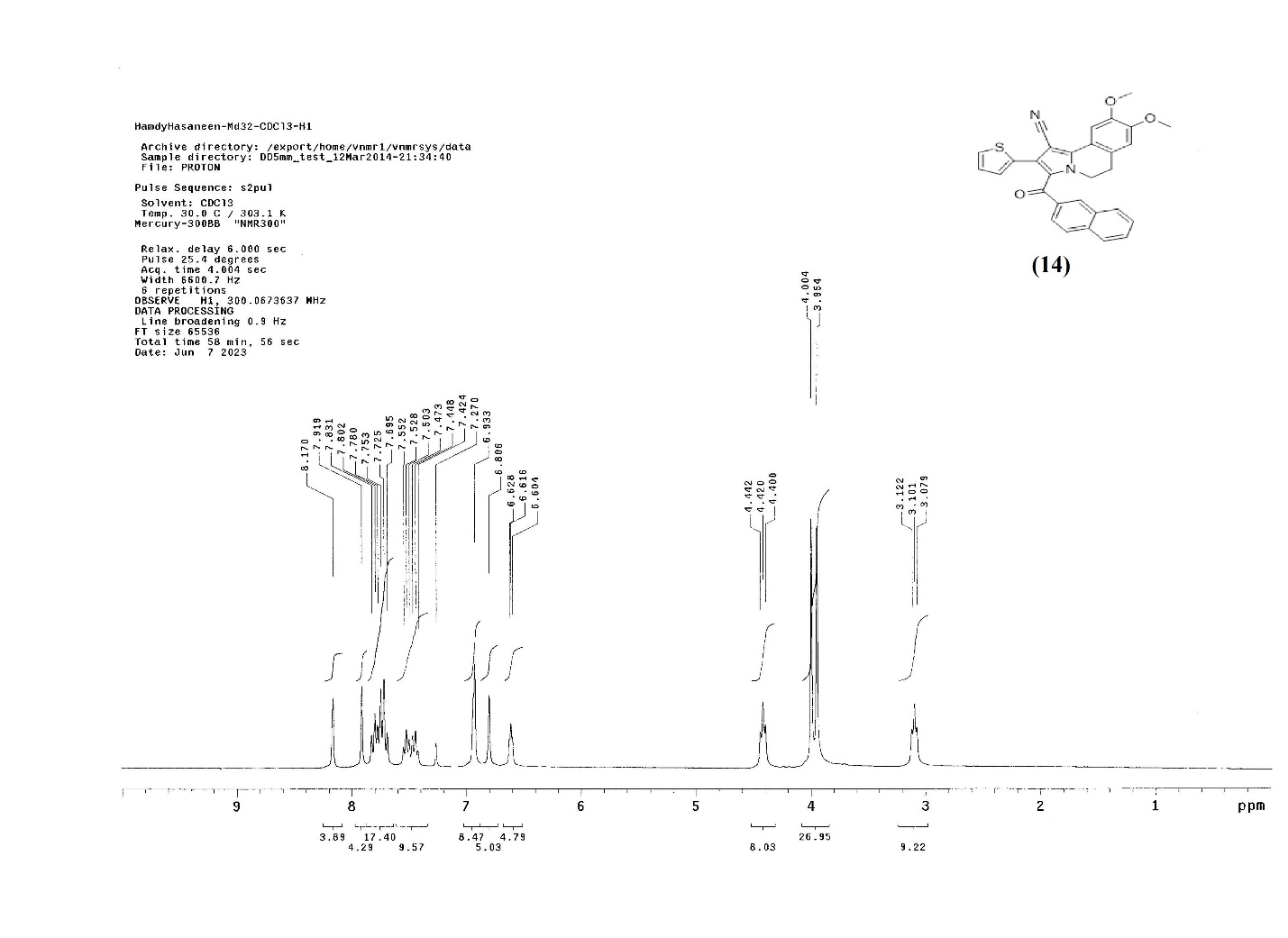

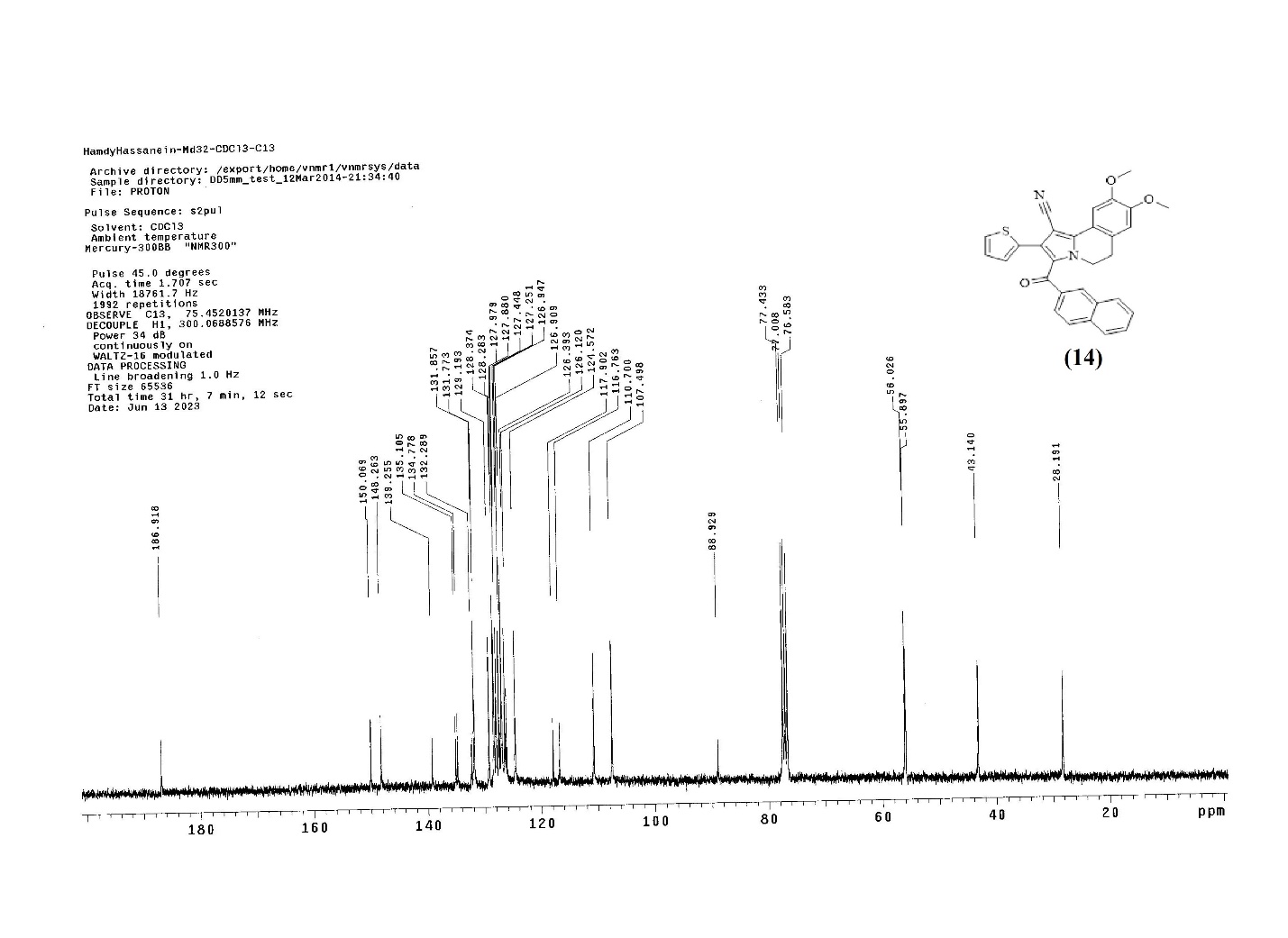

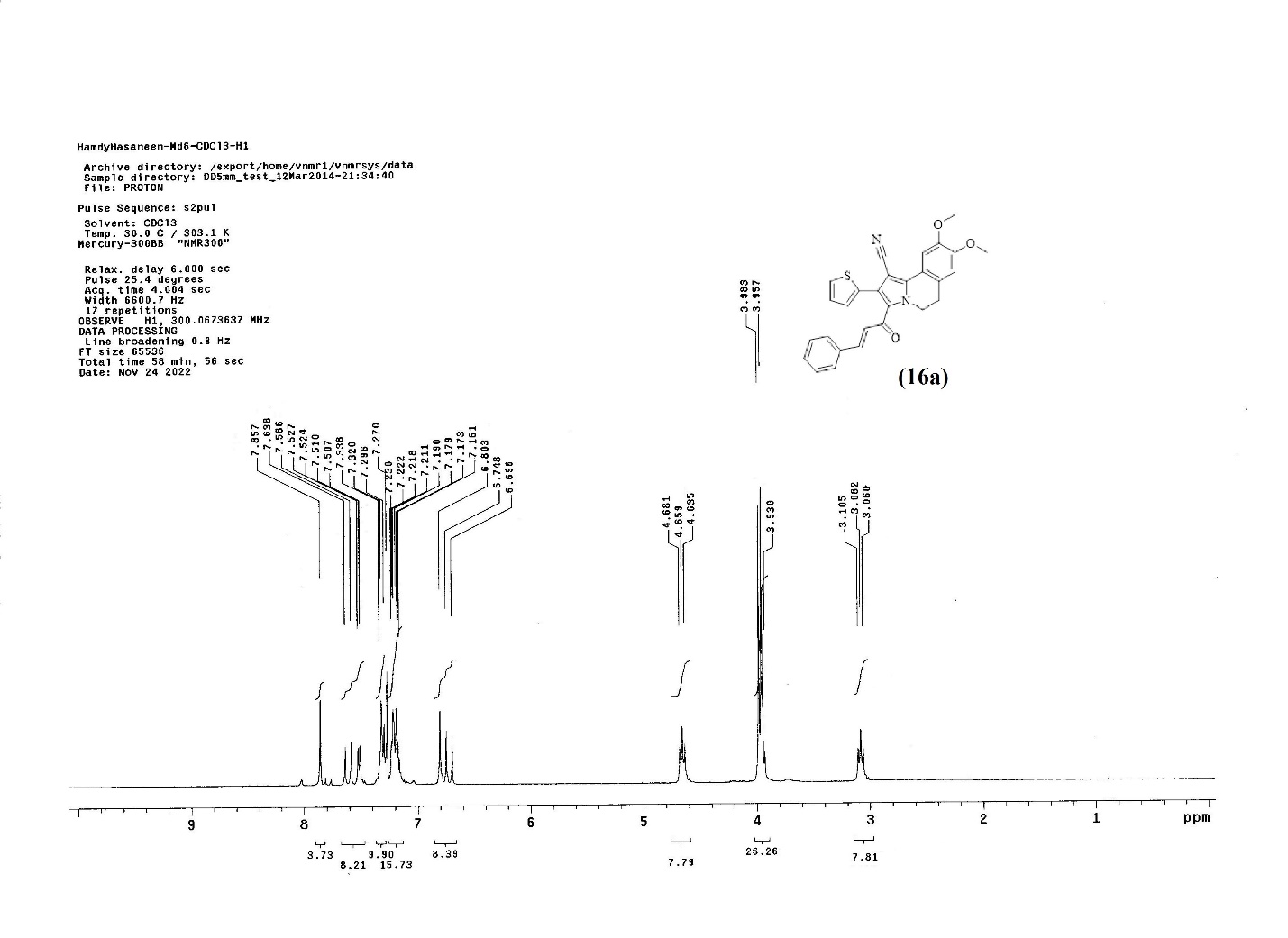


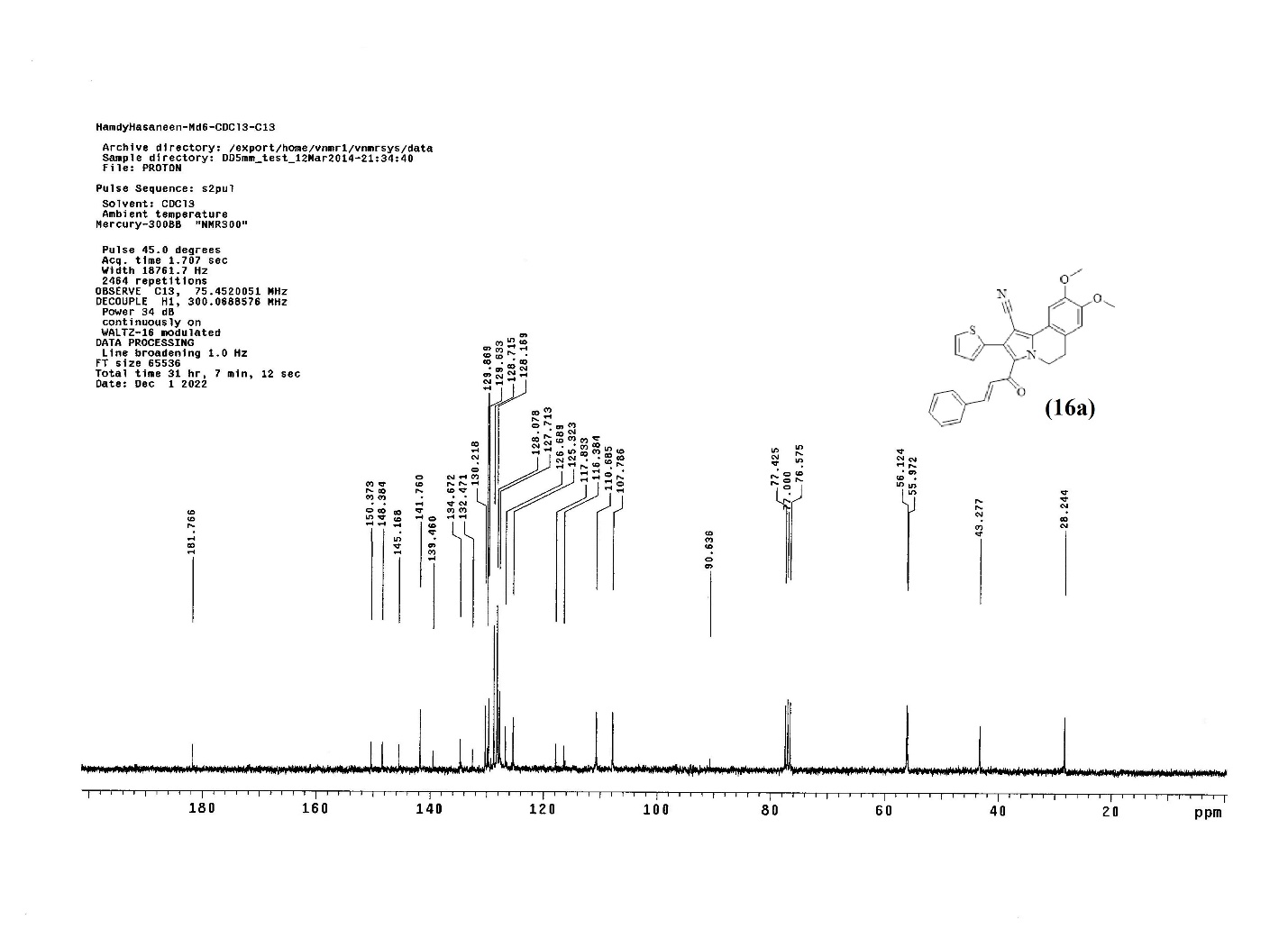

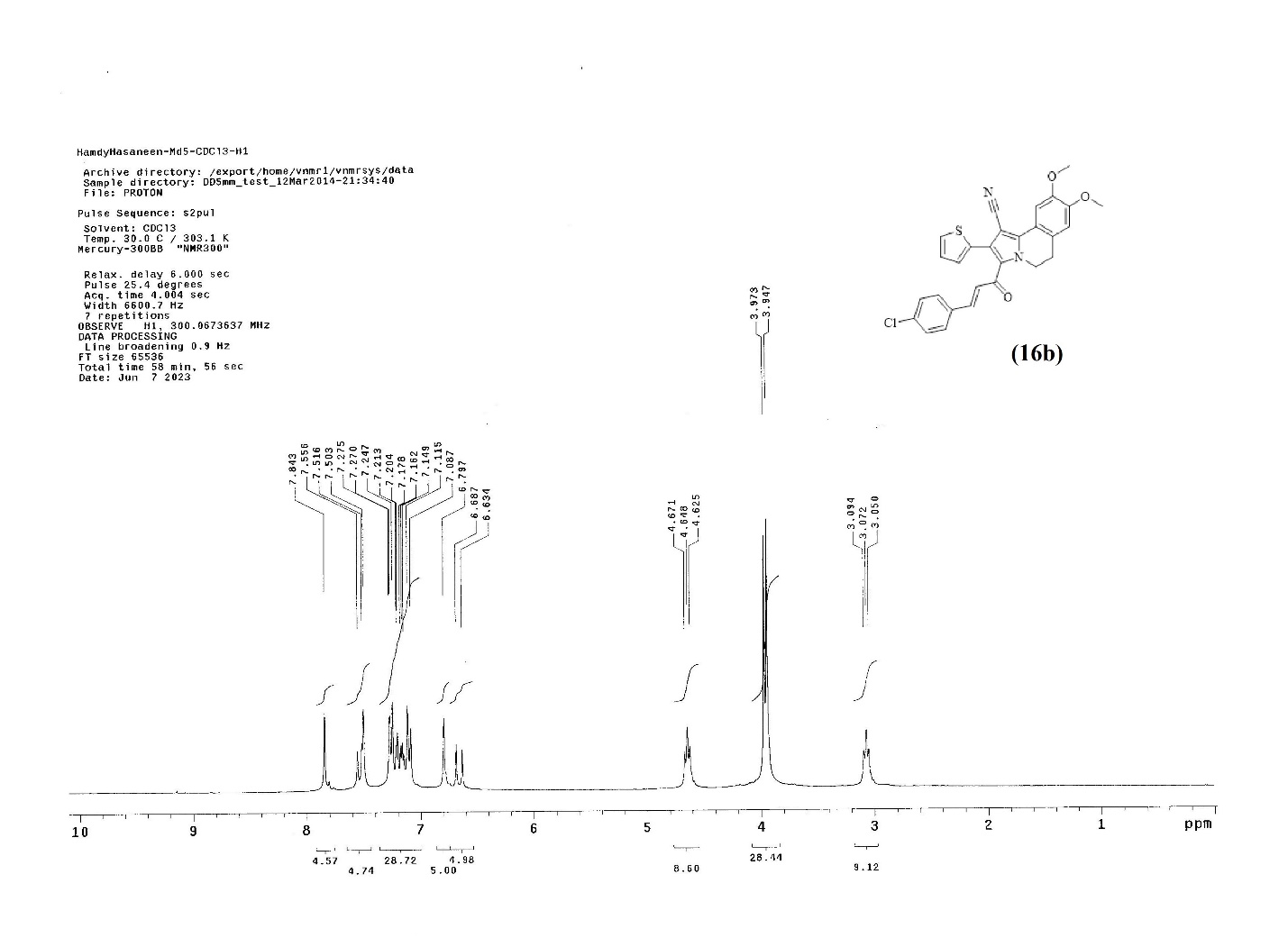

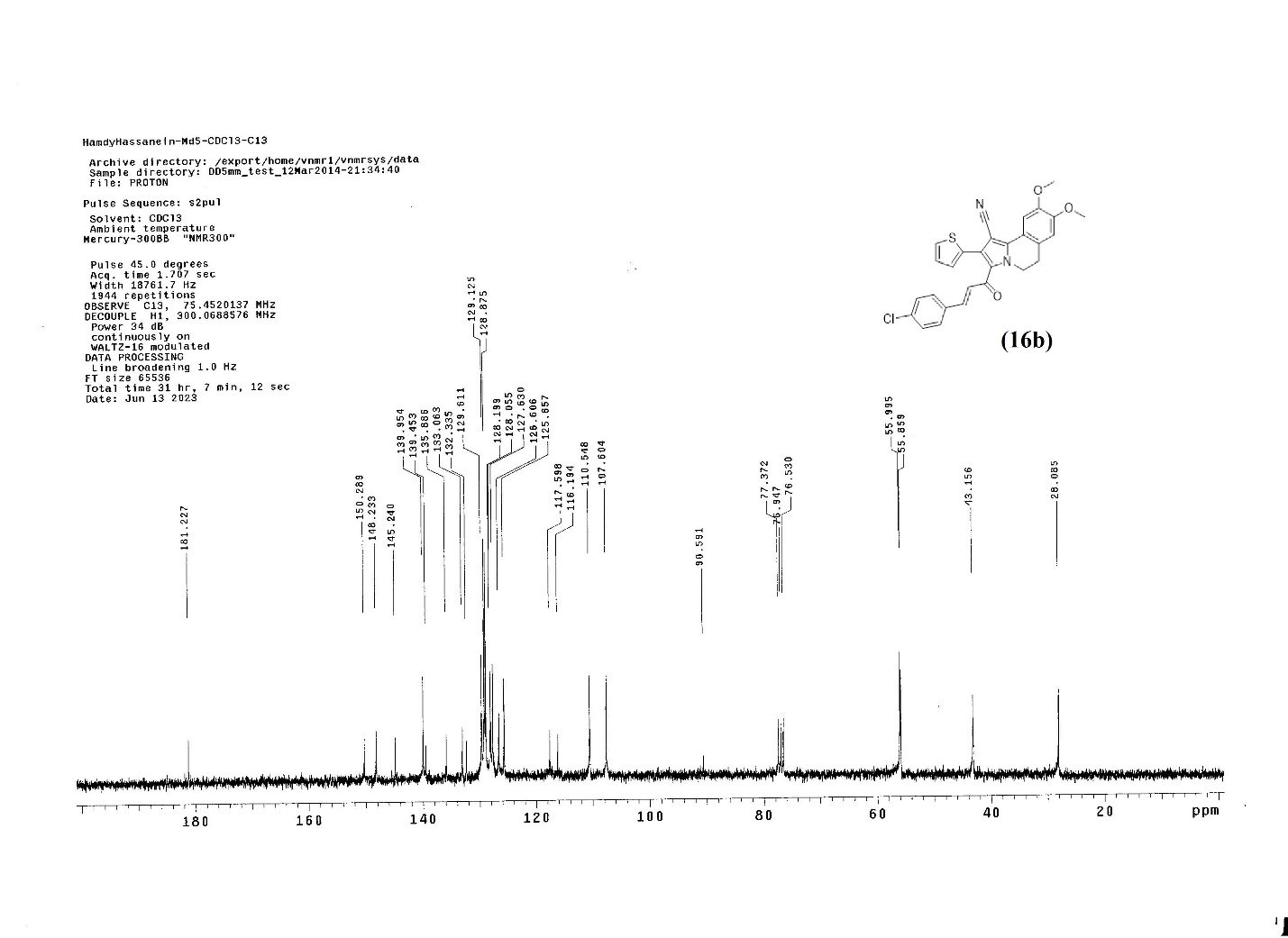

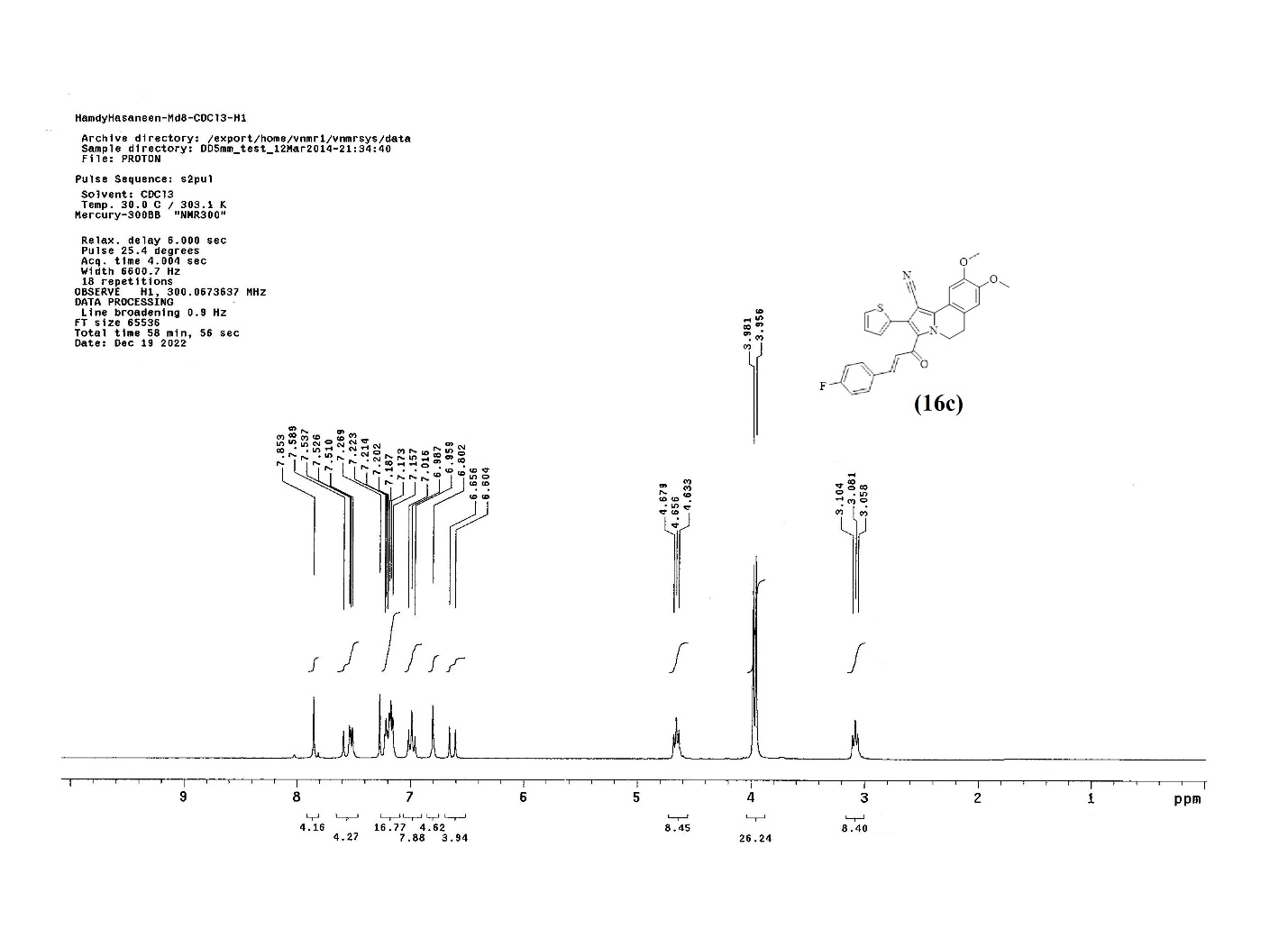

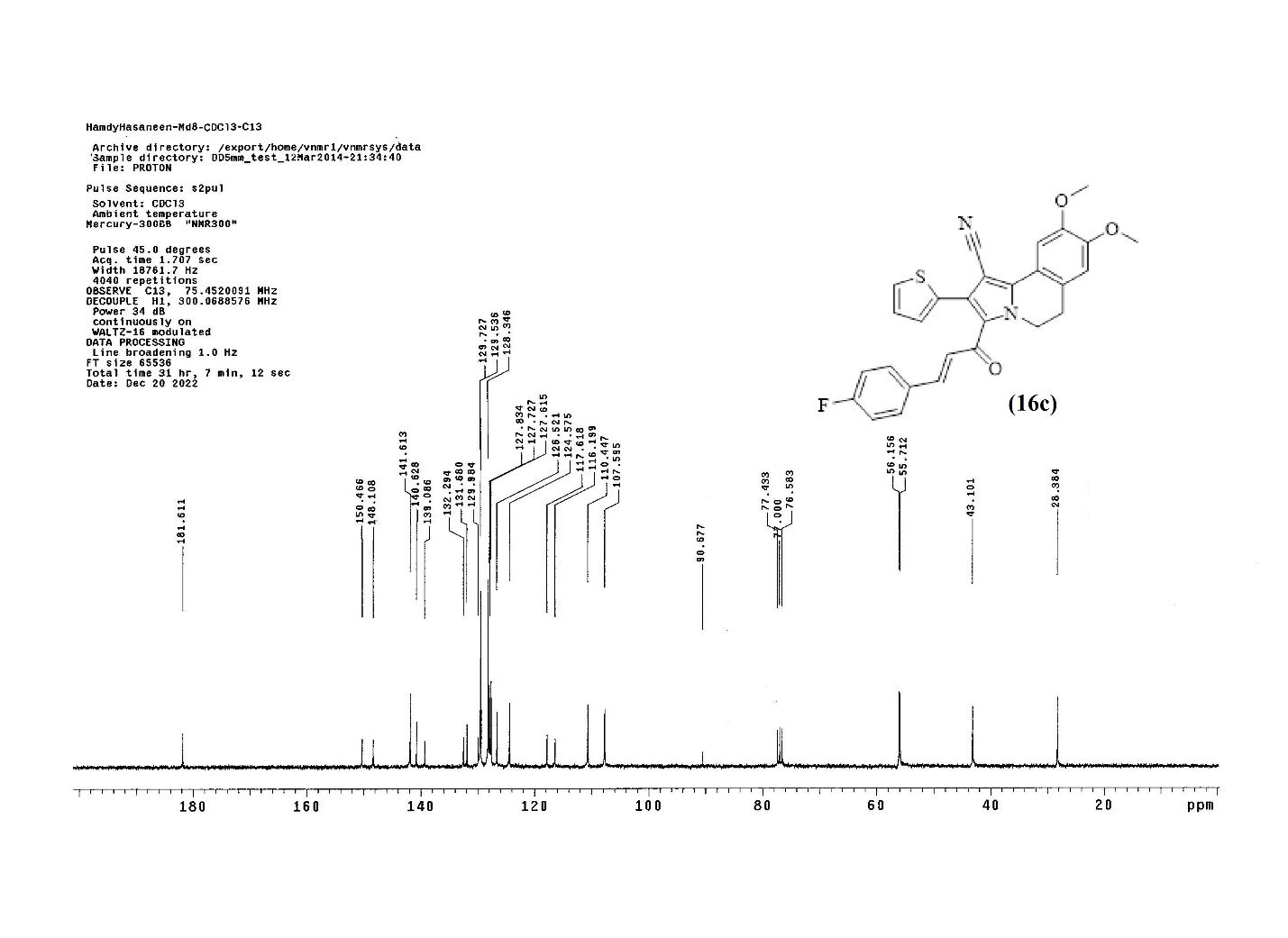

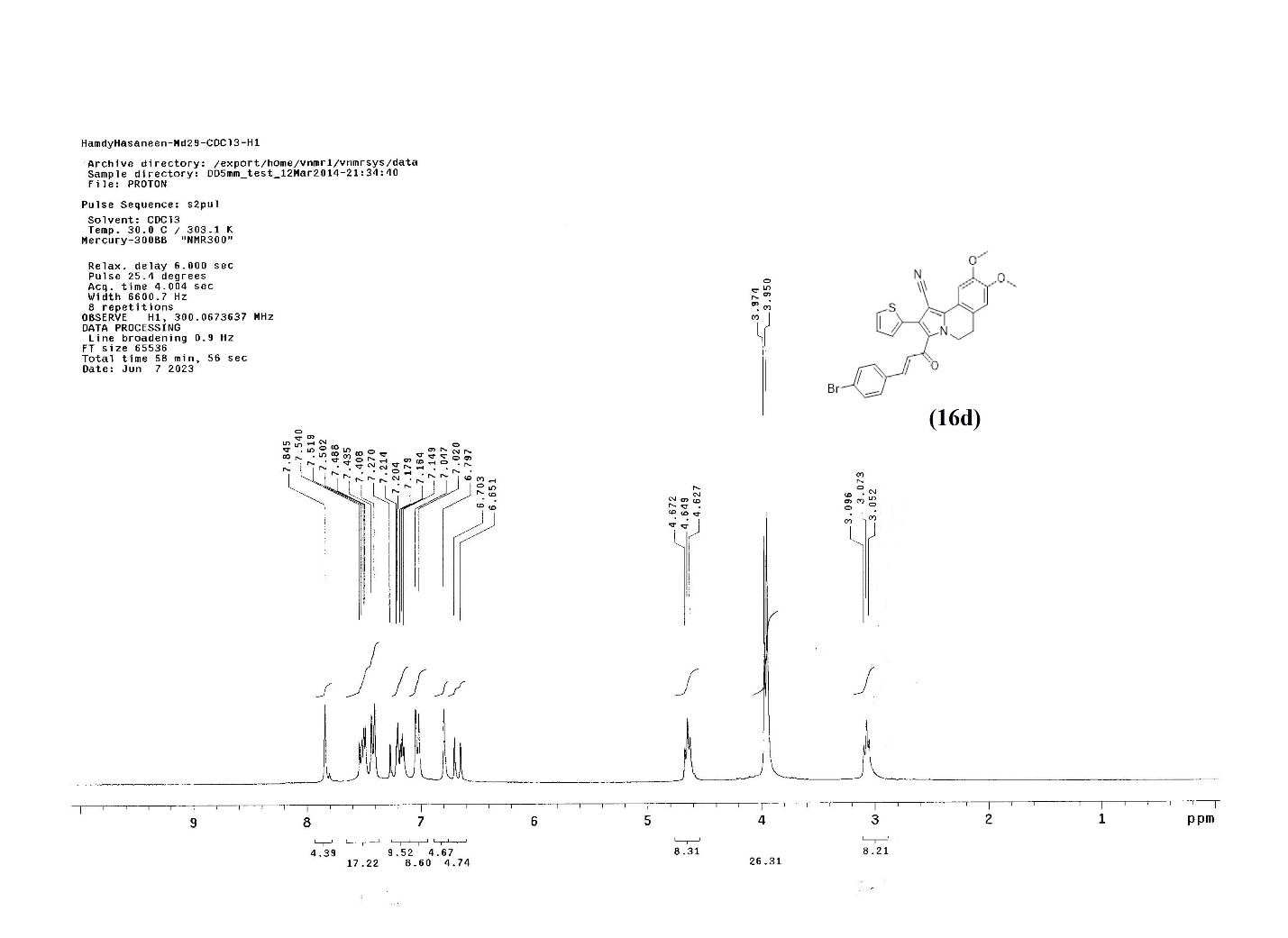

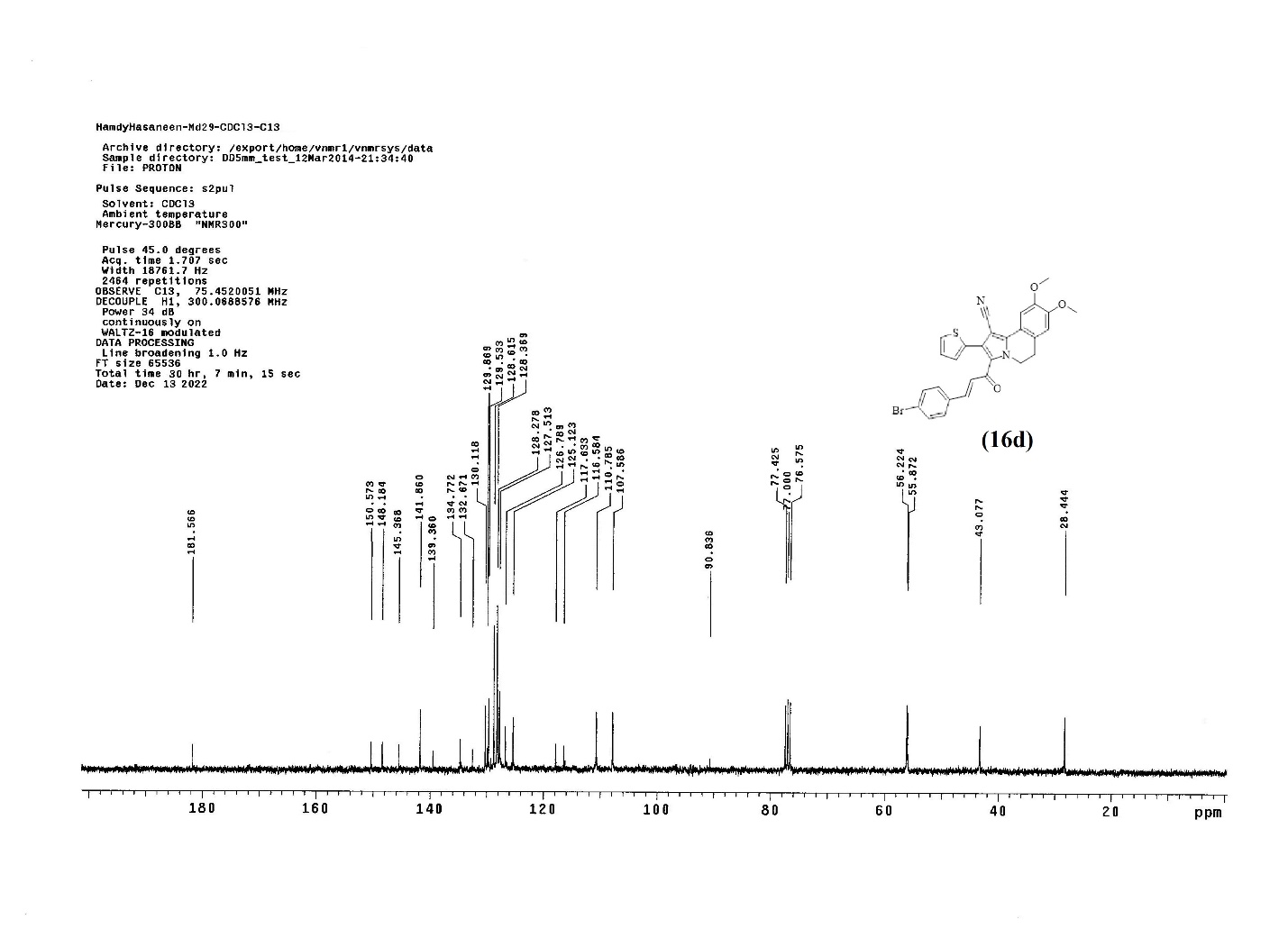

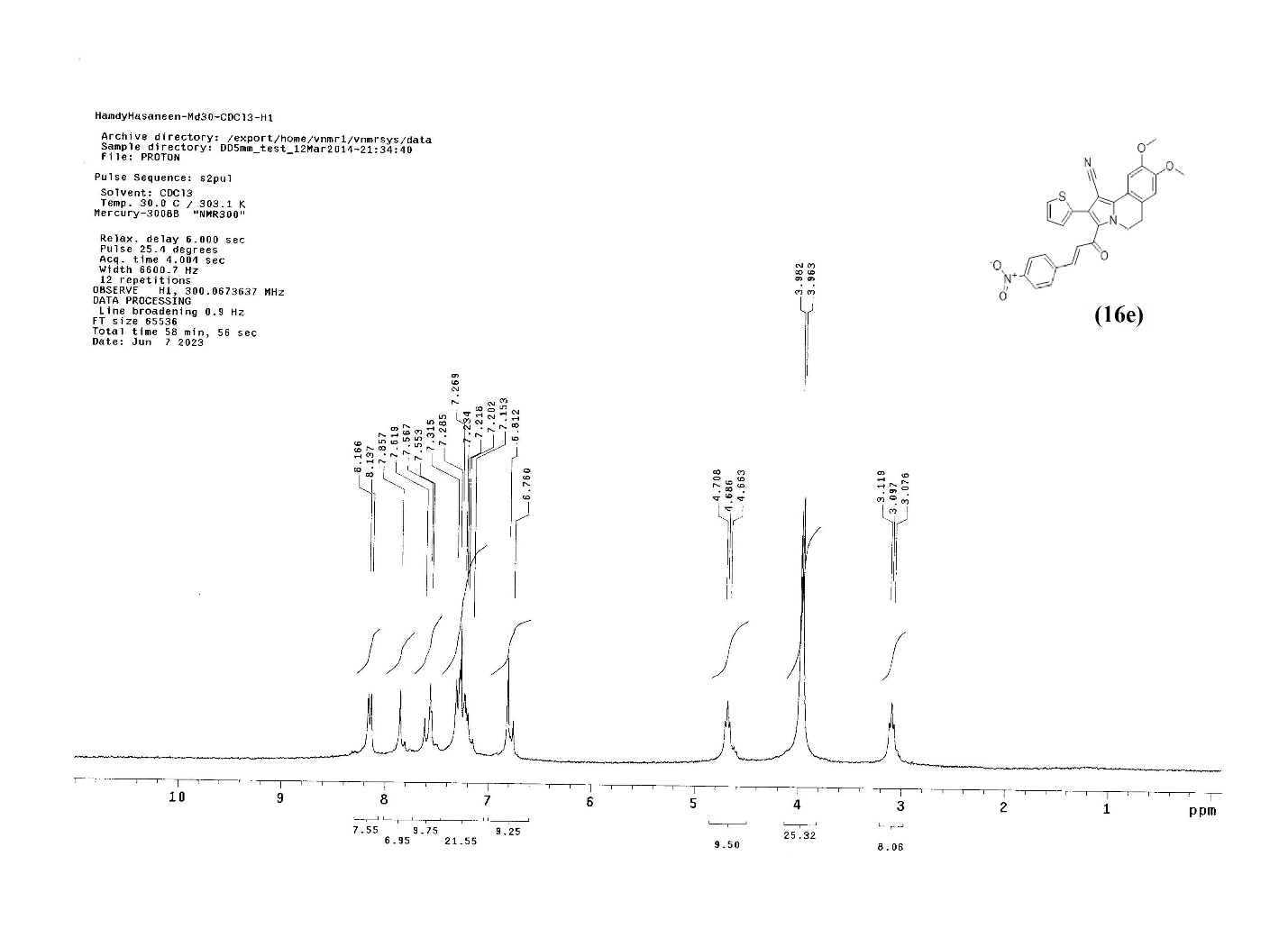

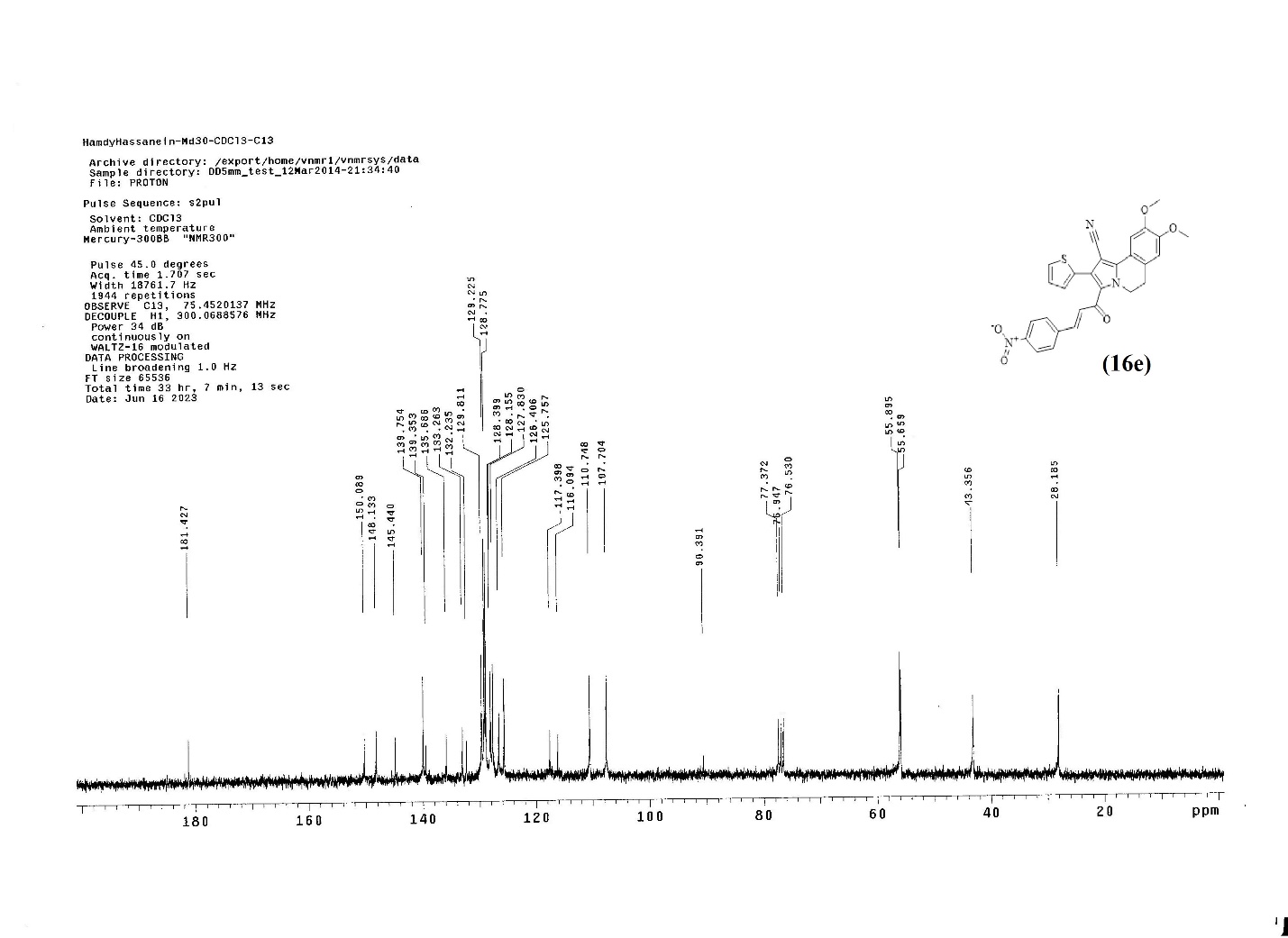

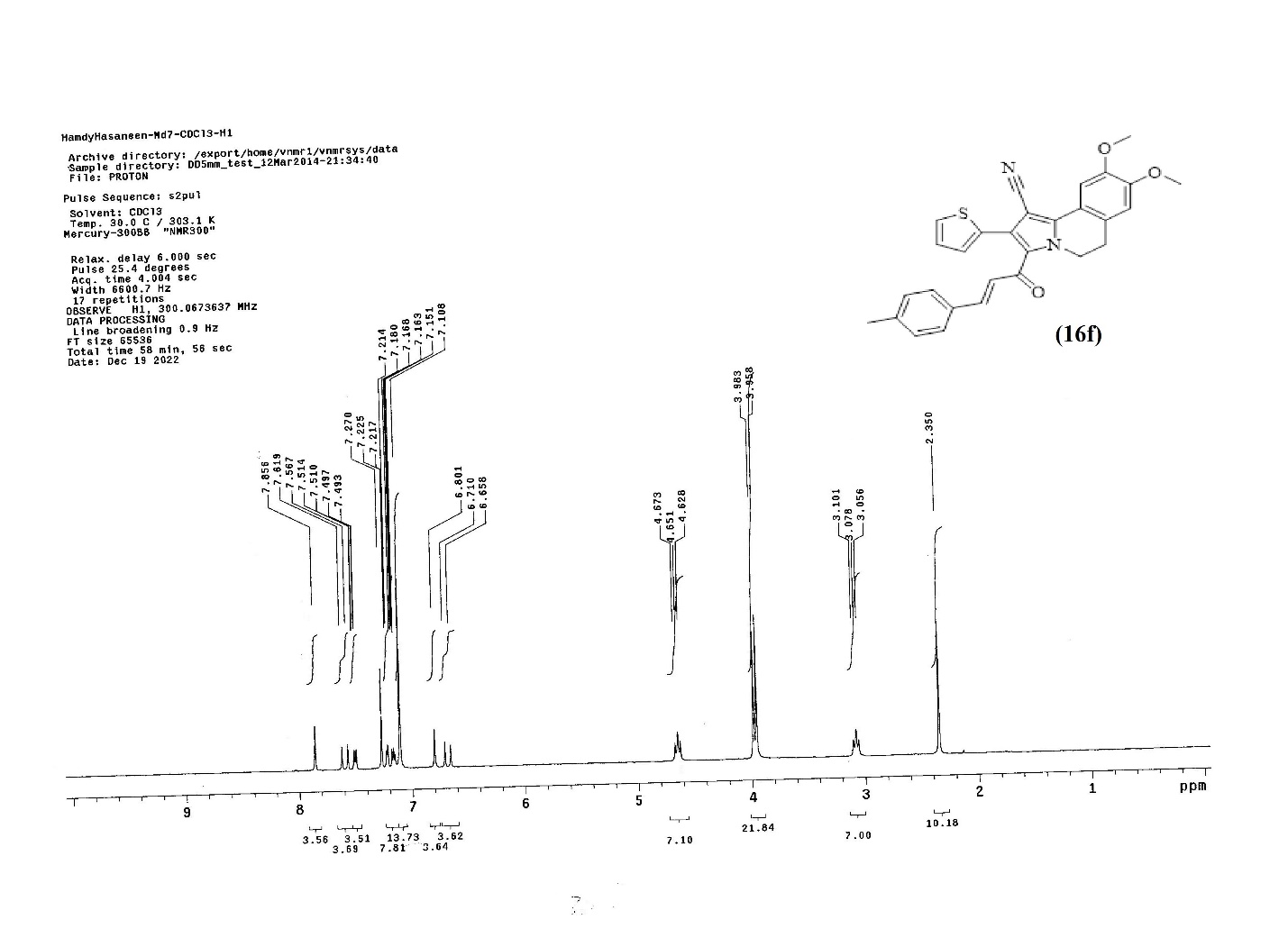

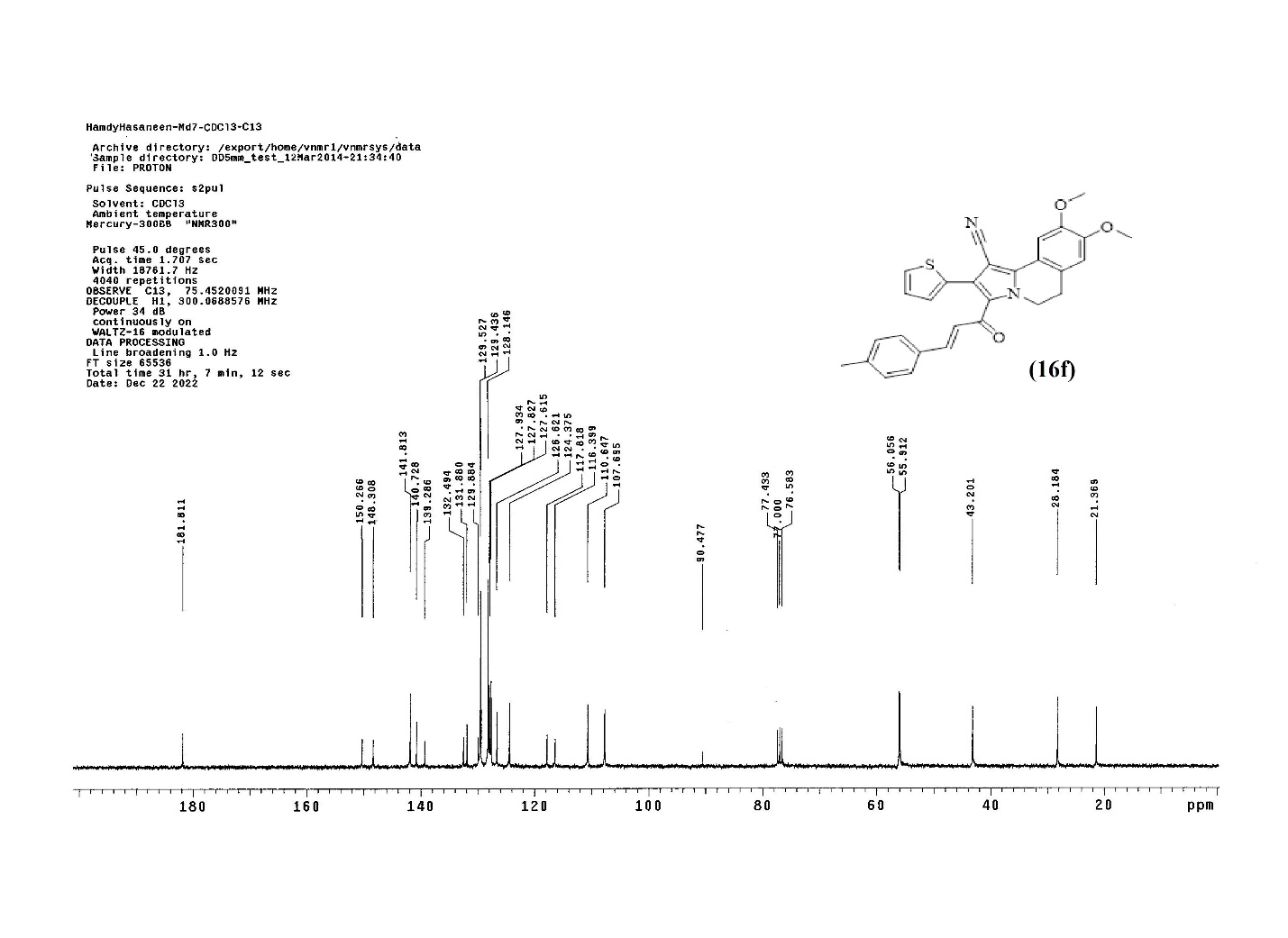

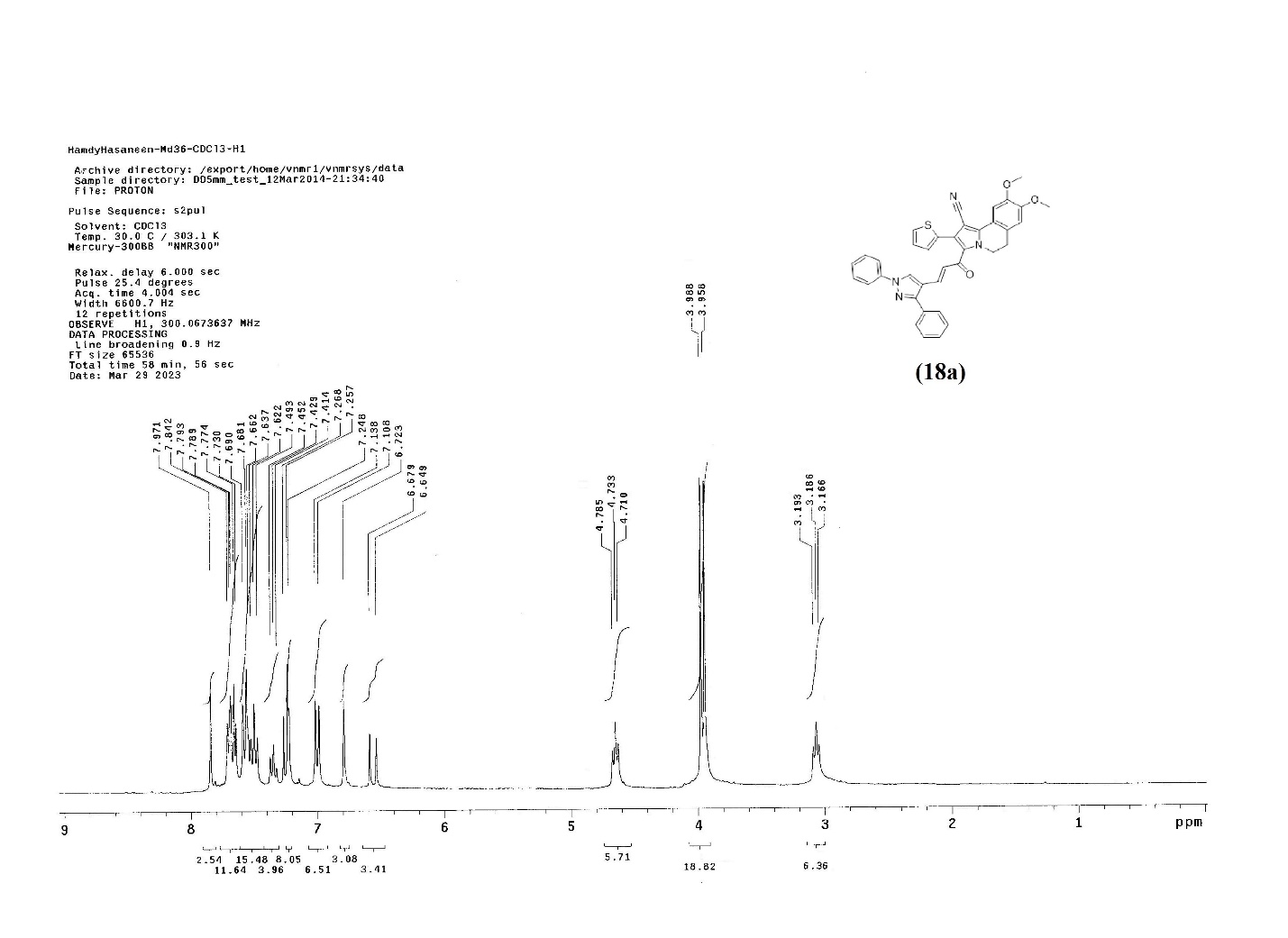

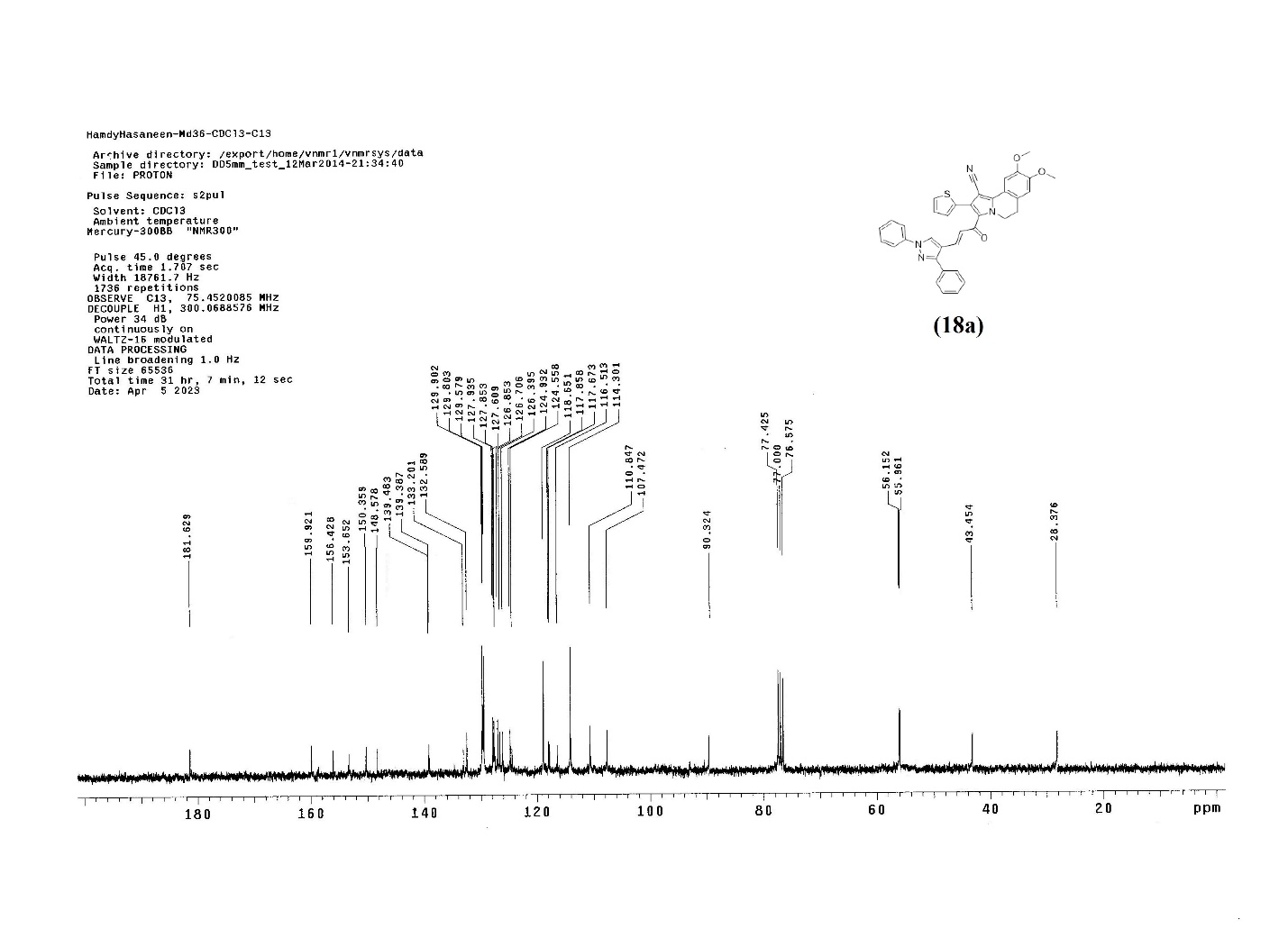

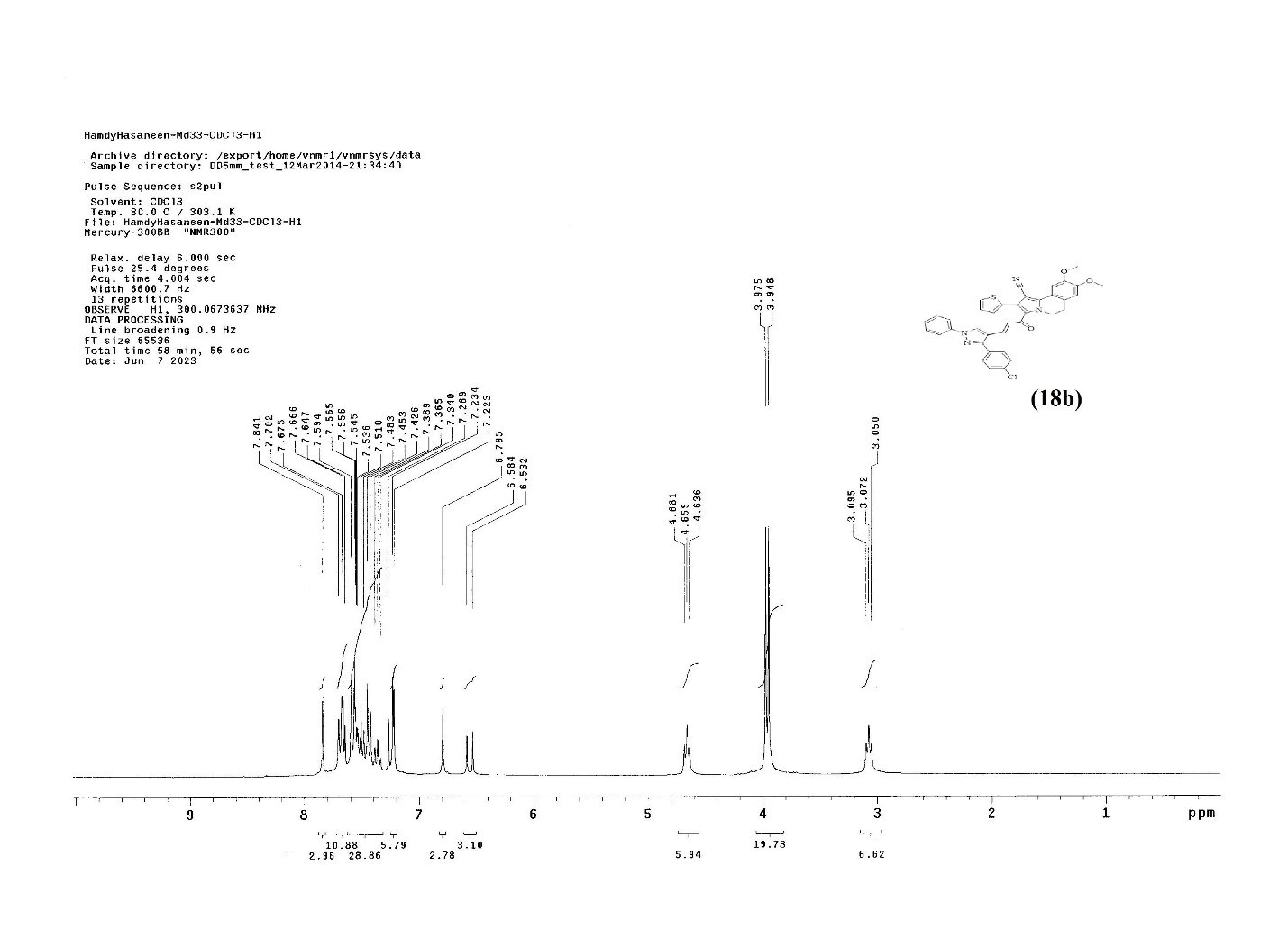

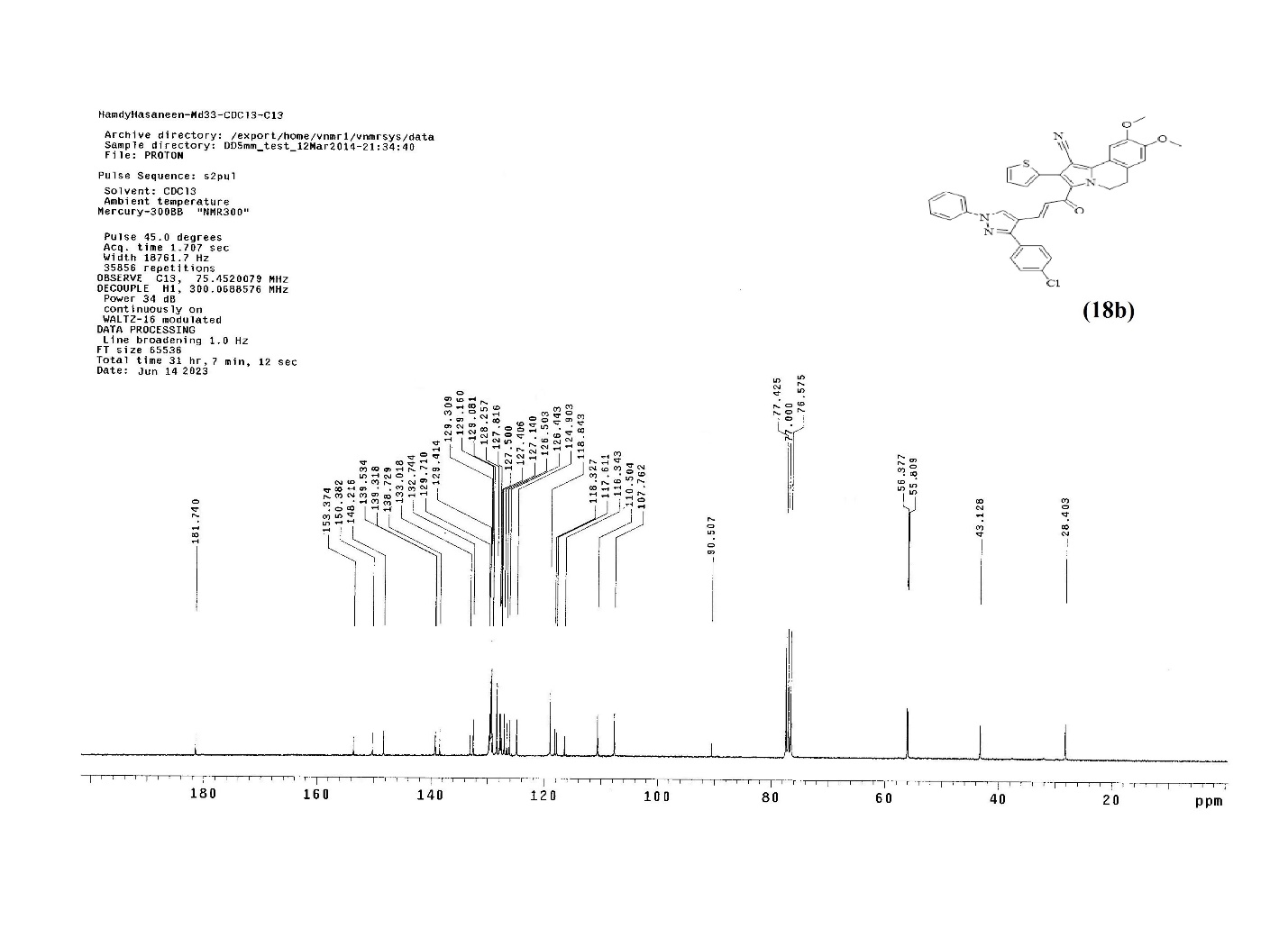

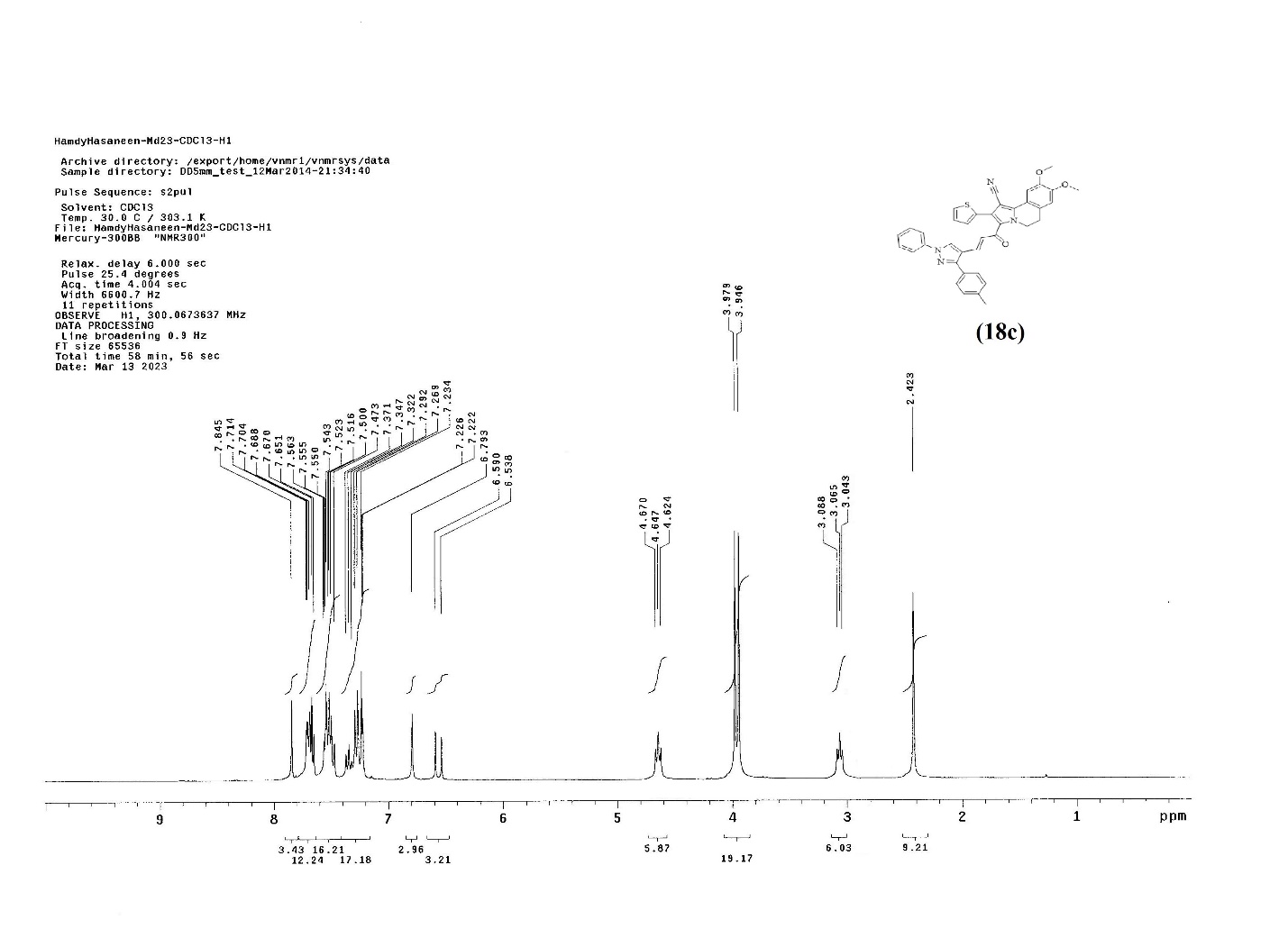

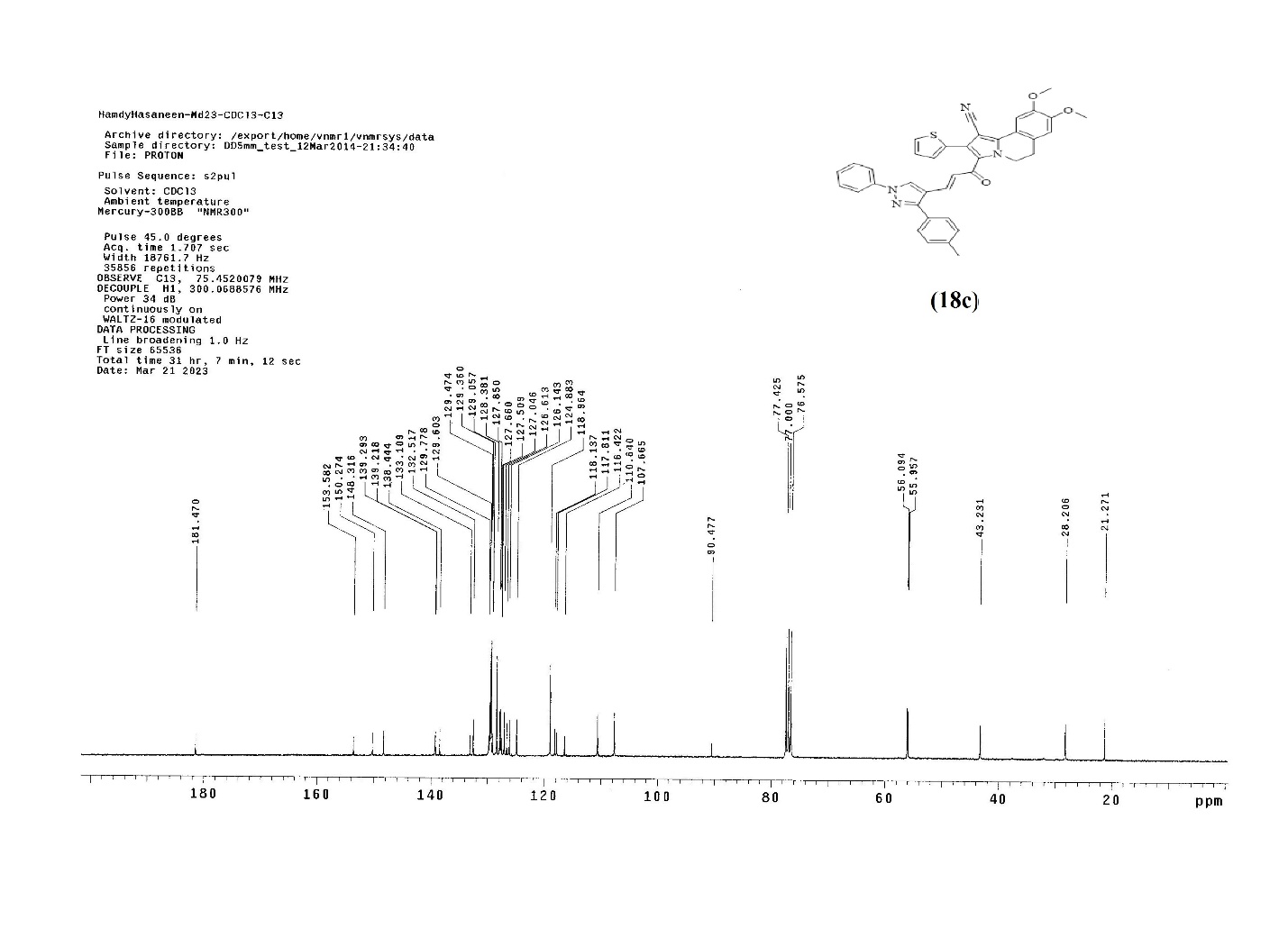

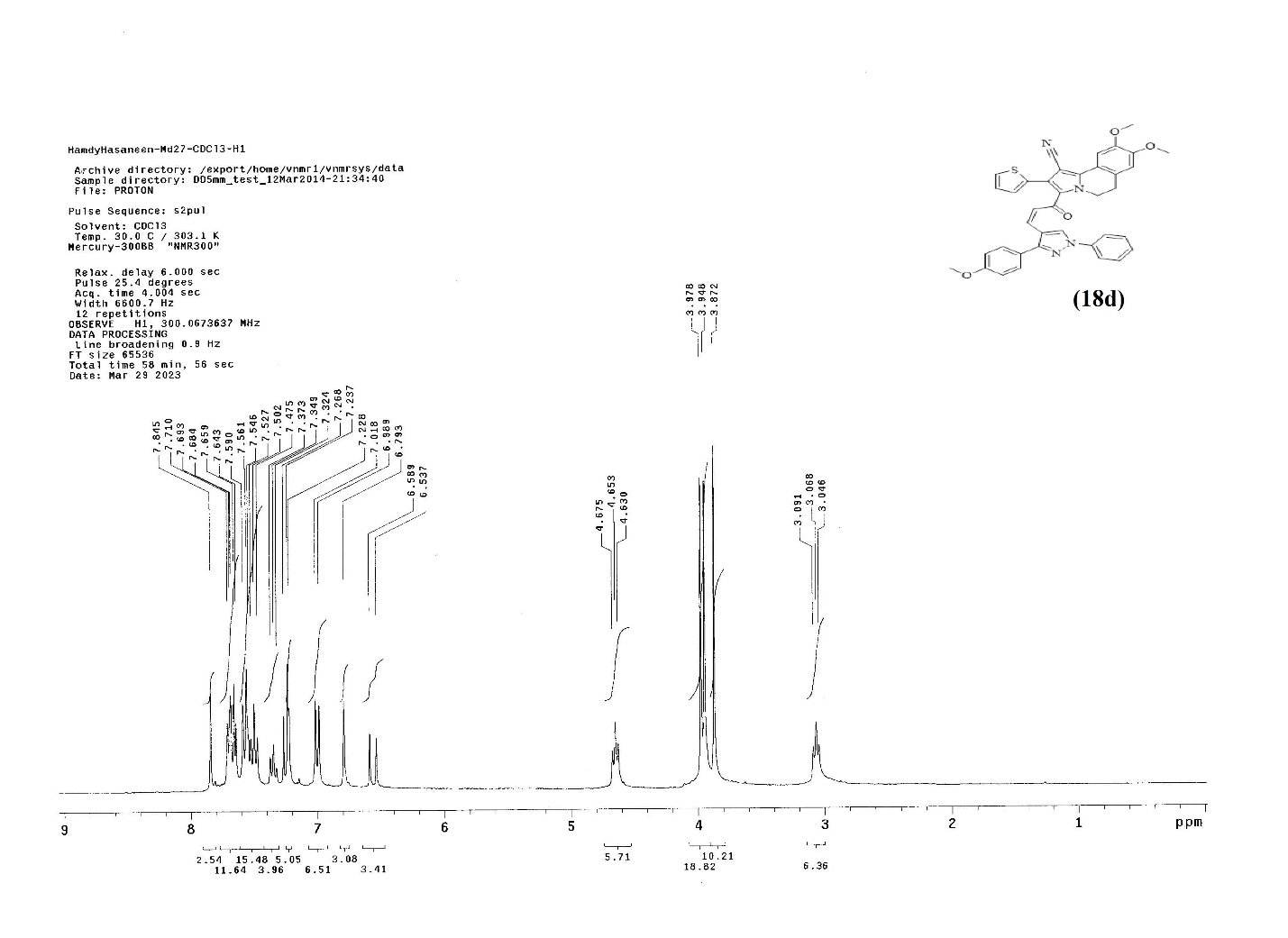

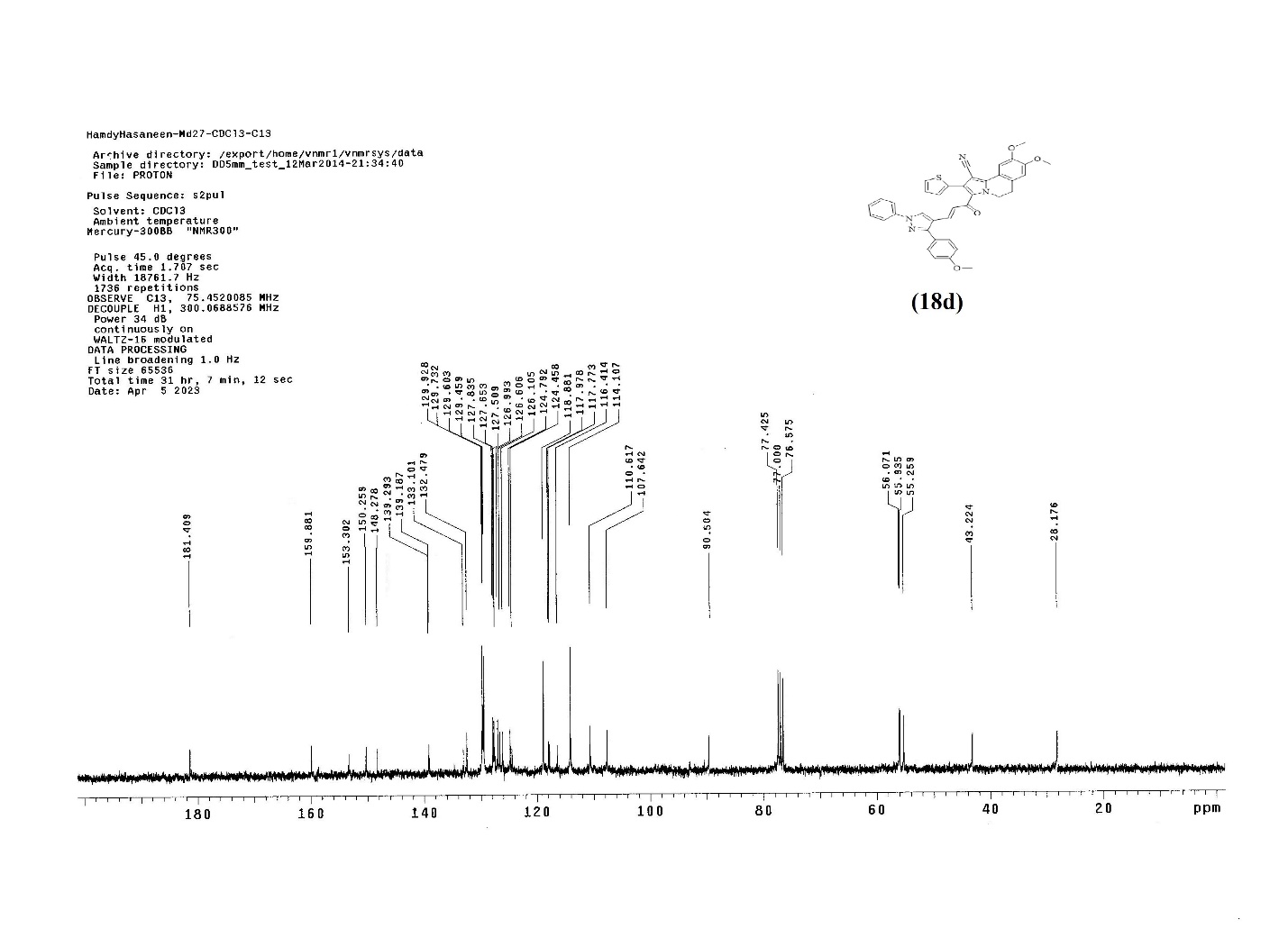

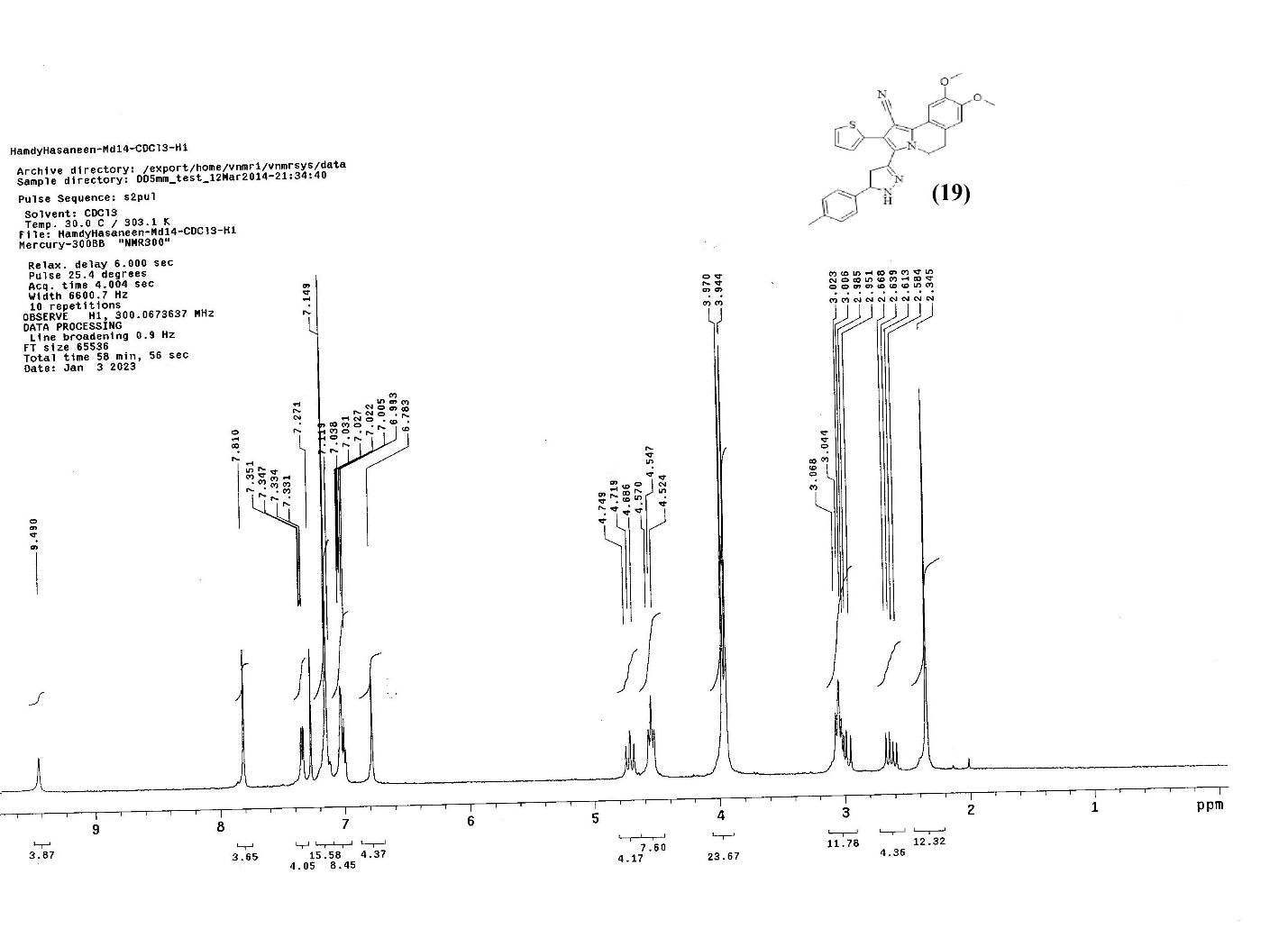

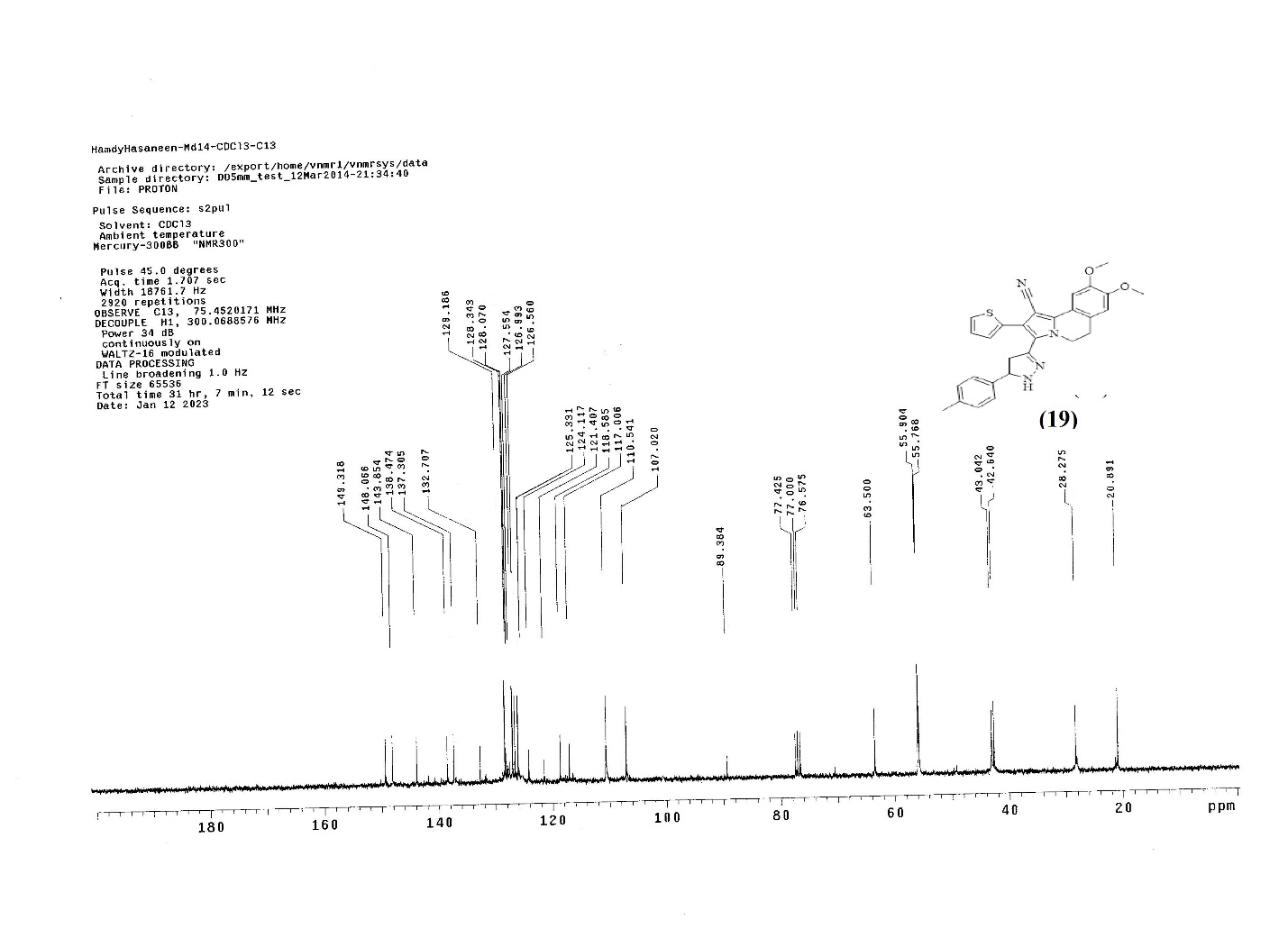

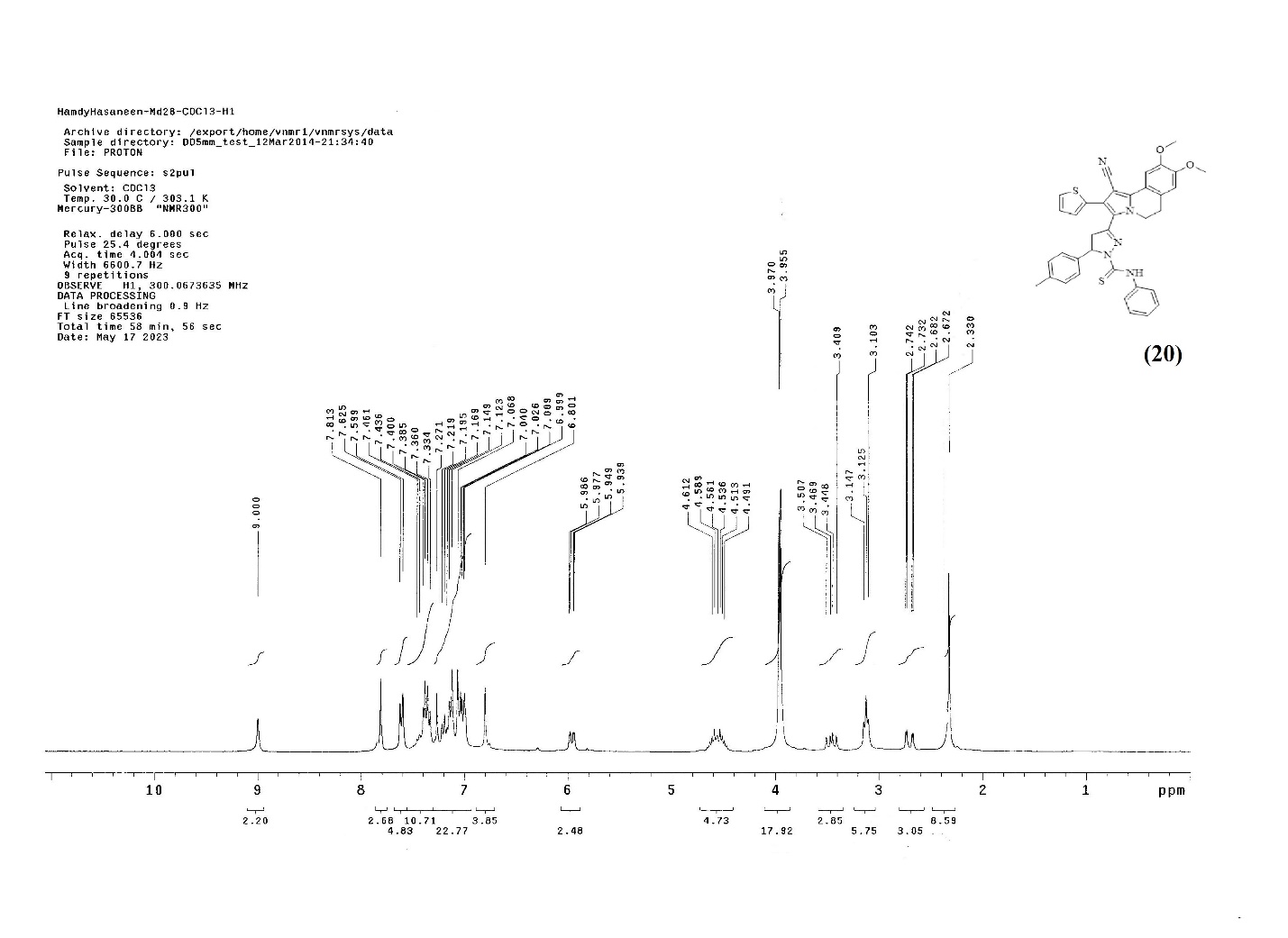

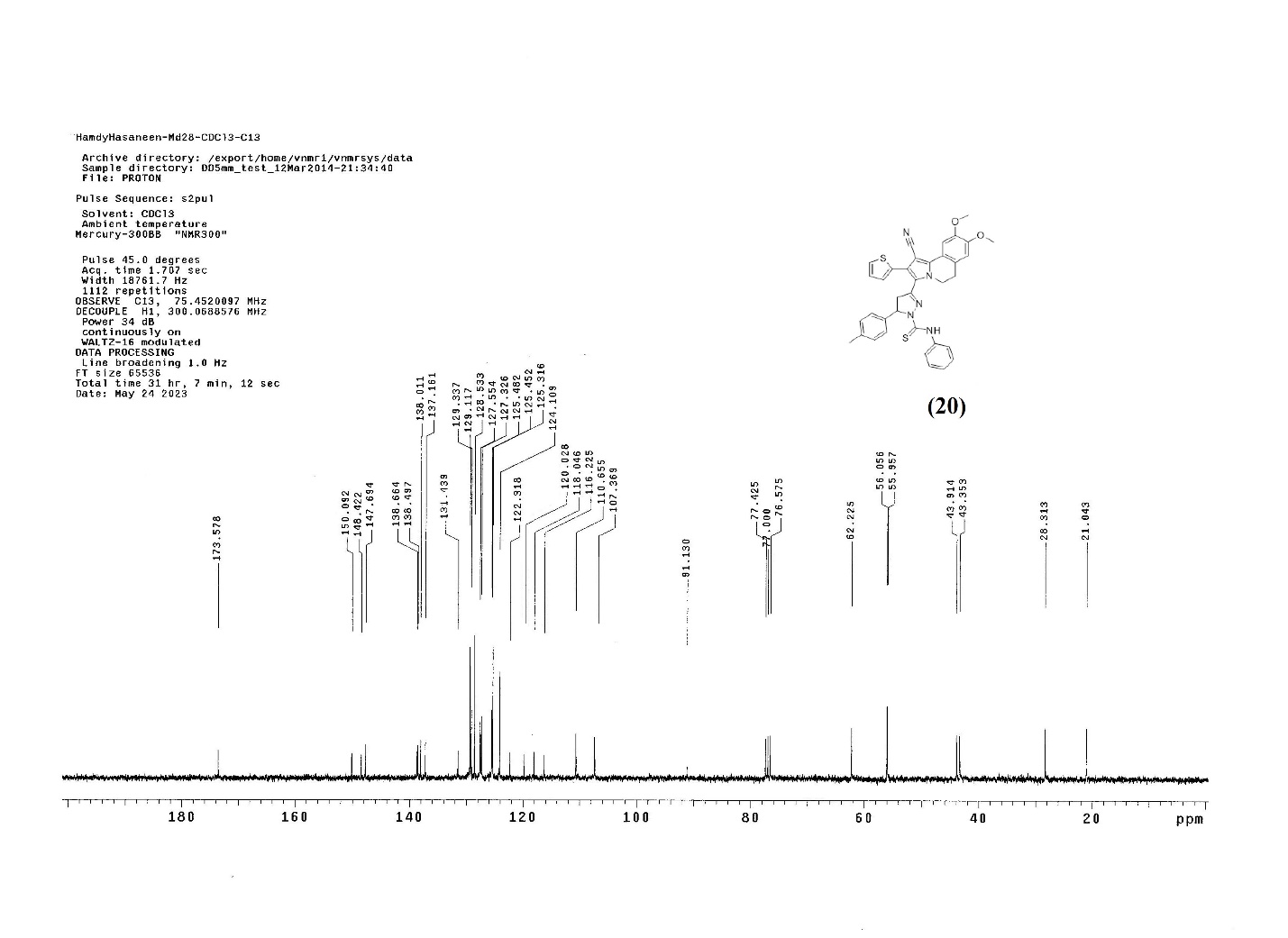

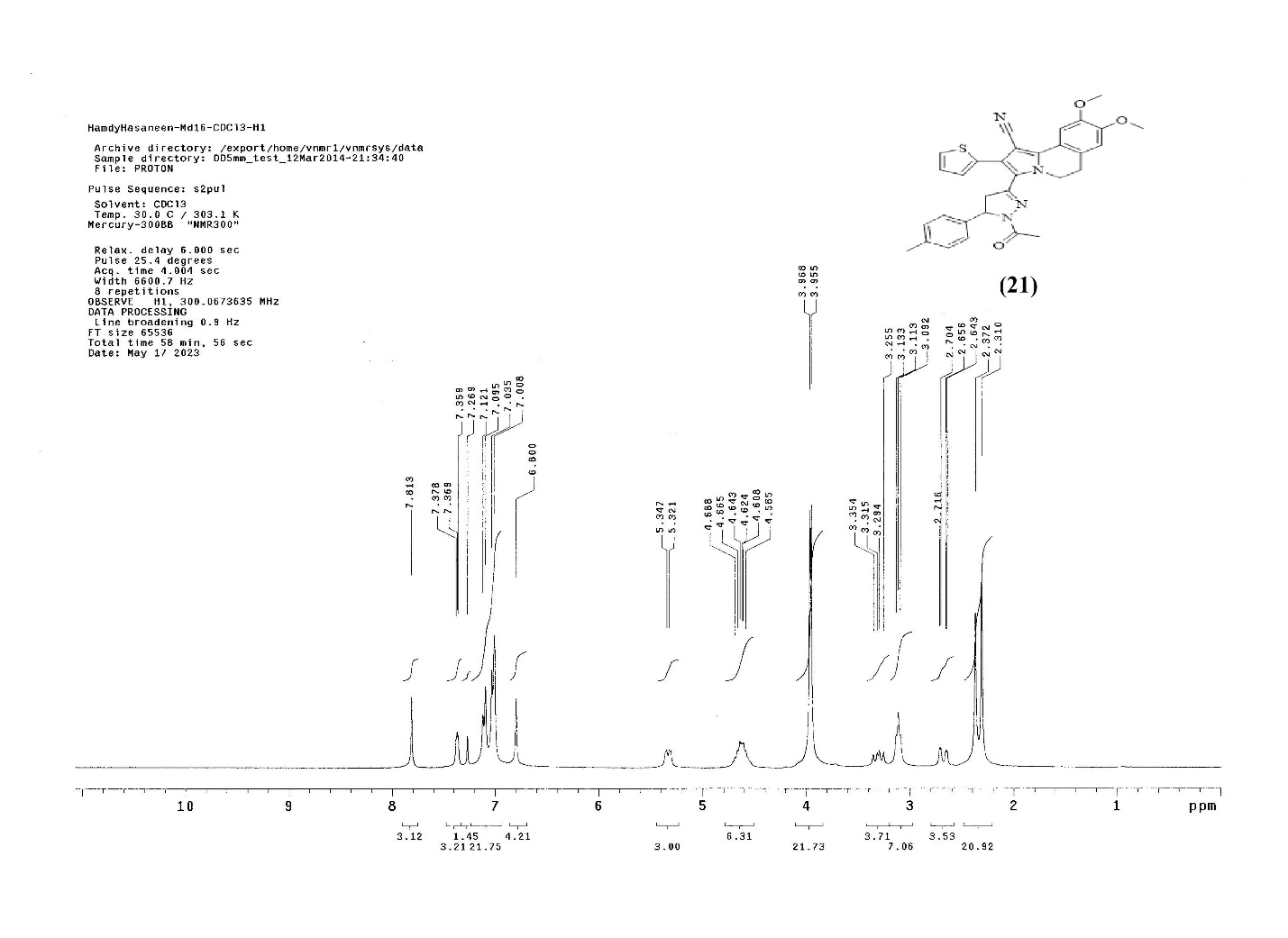

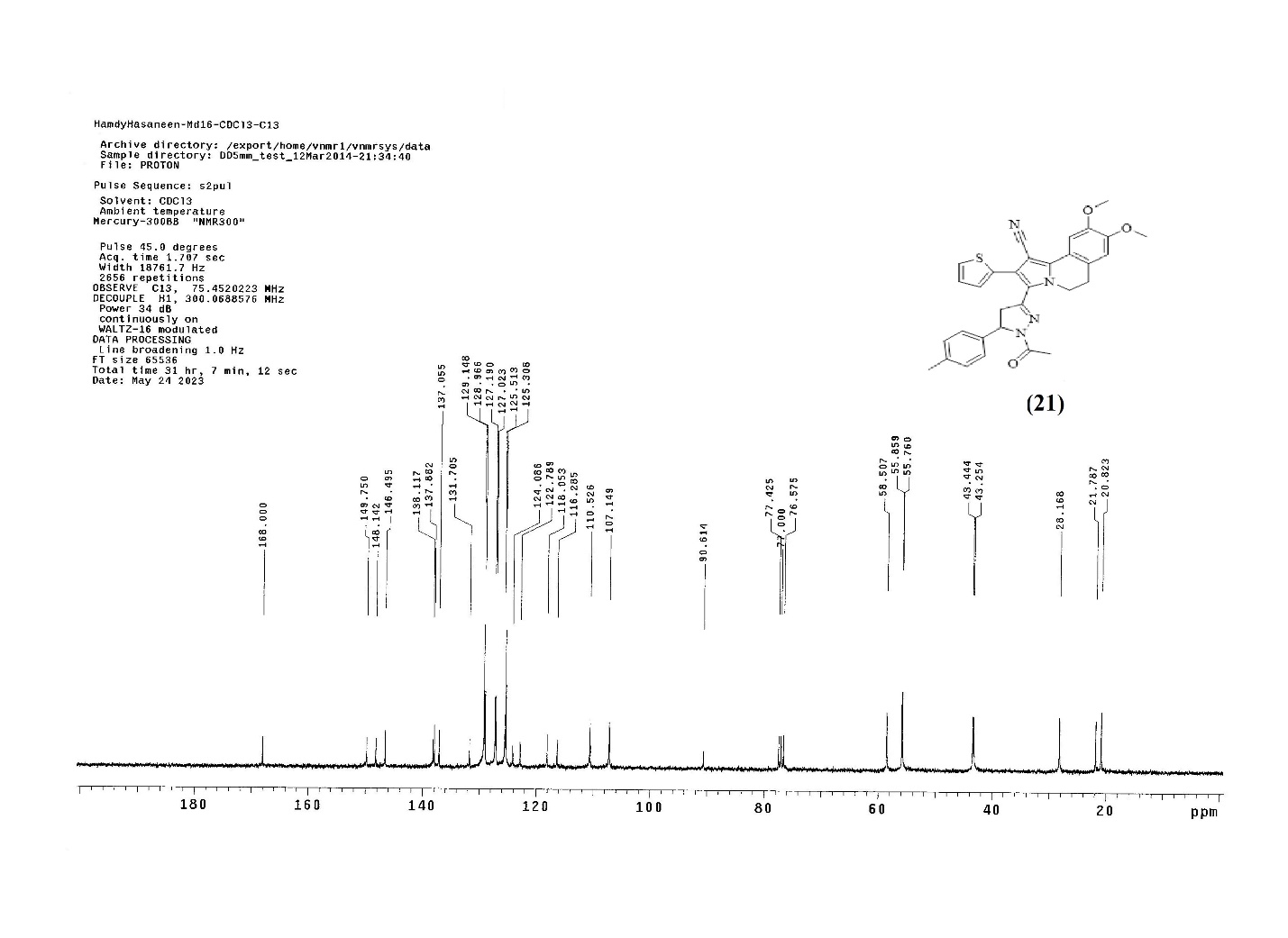

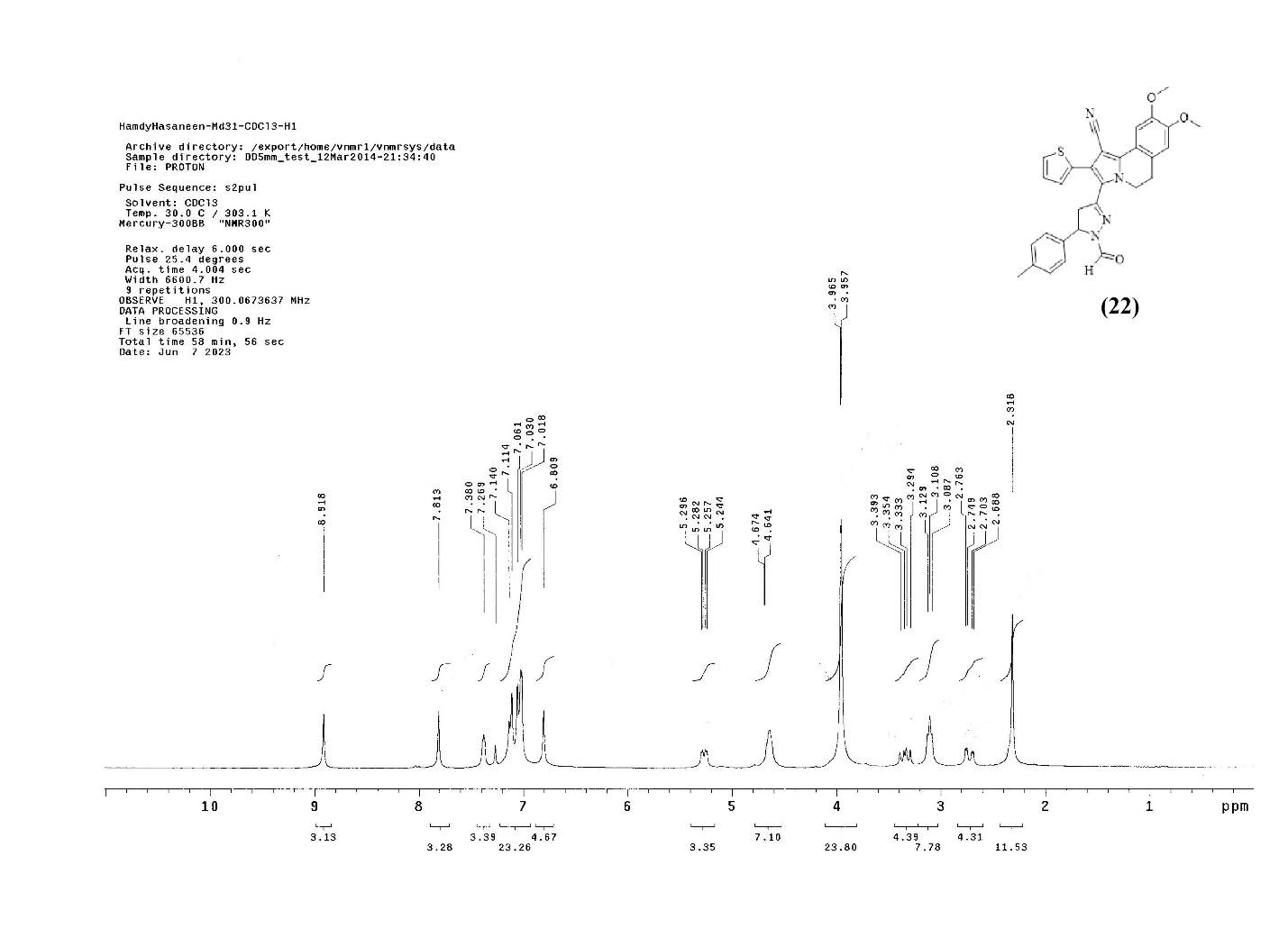

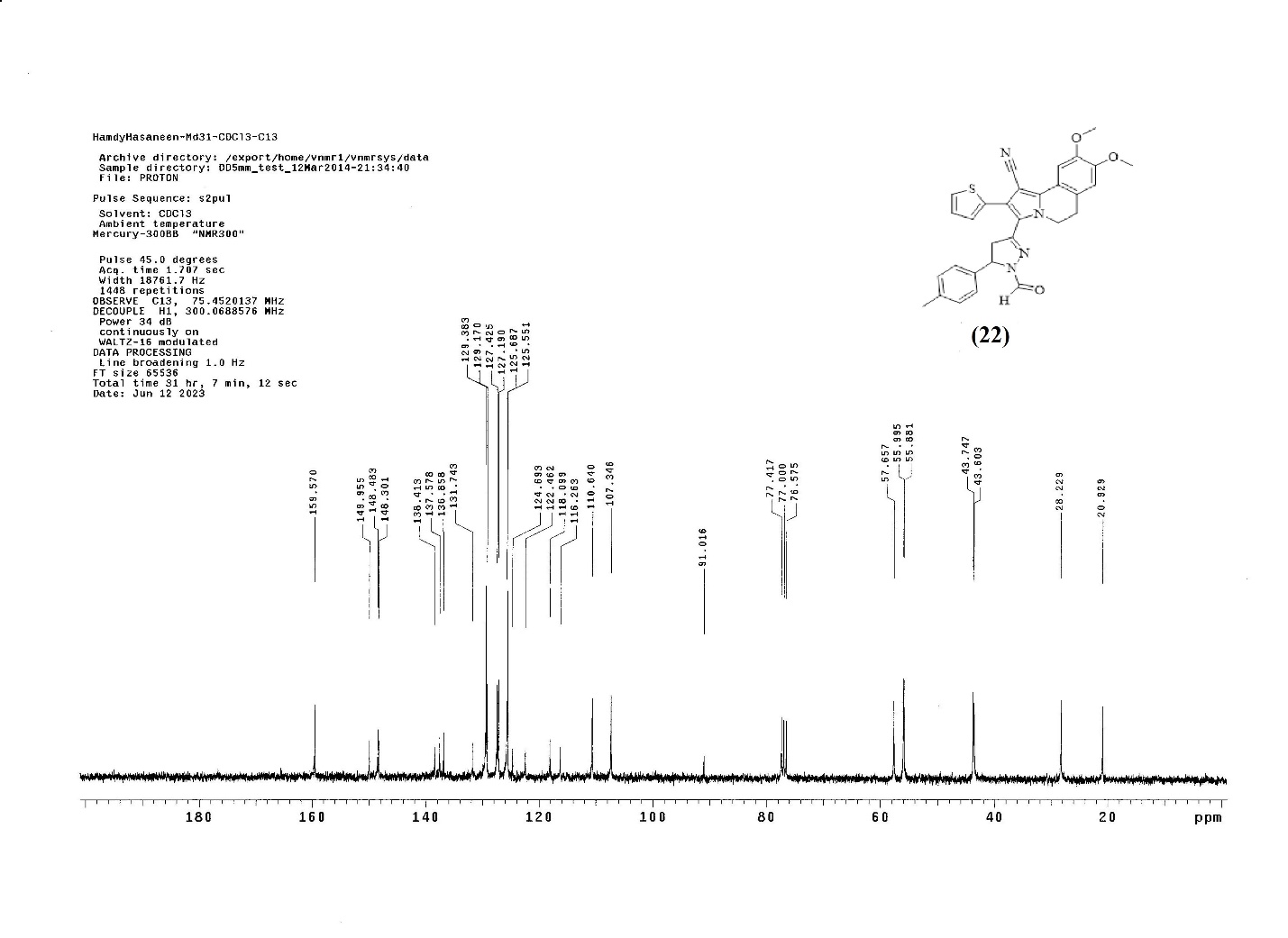

Supplement: Supplementary file 1 — Supplementary Material 1 [file 13065_2025_1557_MOESM1_ESM.docx]
